# Supplementary material for: Methods and results used in the development of a consensus-driven extension to the Consolidated Standards of Reporting Trials (CONSORT) statement for trials conducted using cohorts and routinely collected data (CONSORT-ROUTINE)
Source: BMJ Open. 2021 Apr 29;11(4):e049093. doi: 10.1136/bmjopen-2021-049093 (PMC8094349; doi:10.1136/bmjopen-2021-049093)
Supplement: Supplementary data [file bmjopen-2021-049093supp001.pdf]

## Supplementary File 1 – Electronic Search Strategies

Searches were run in both MEDLINE and Cochrane Methodology Register simultaneously. As an example, in the registries search, lines 1-11 are the MEDLINE search and lines 12-15 are tailored for the Cochrane Methodology Register. The final lines of each search isolate the records from each database, combine them so duplicate records can be removed, then isolate the remaining records so they can be downloaded and imported into Reference Manager using customized import filters.

**Searches for RCTs embedded in Registries**

1. ((registry or registries) adj5 randomi#ed).ab,kf,ti.
2. ((registry or registries) adj5 RCT*).ab,kf,ti.)
3. ((registry or registries) adj5 controlled trial*).ab,kf,ti.
4. ((registry or registries) adj5 (RRCT* or R RCT*)).ab,kf,ti.
5. or/1-4
6. (meta analy* or metaanaly* or metanaly* or systematic review*).af.
7. 5 not 6
8. Registries/
9. limit 8 to randomized controlled trial
10. 7 or 9
11. limit 10 to yr="2007 - 2018"
12. (registry or registries).ab,kf,ti.
13. (random* or RCT).ti,ab,kw.
14. 12 and 13
15. limit 14 to yr="2007 - 2018"
16. 11 use medall
17. 15 use clcmr
18. 16 or 17 (1240)
19. remove duplicates from 18
20. 19 use medall
21. 19 use clcmr

**Searches for RCTs embedded in Cohorts**

1. (cohort adj5 (randomi#ed adj5 trial*)).ab,kf,ti.
2. (cohort adj5 RCT*).ab,kf,ti.
3. (cohort adj5 controlled trial*).ab,kf,ti.
4. (cmRCT or Cohort Multiple Randomised Controlled Trial*).ab,kf,ti.
5. or/1-4
6. cohort.af.
7. (embed* adj8 randomi#ed).ab,kf,ti.
8. (embed* adj8 RCT*).ab,kf,ti.
9. (embed* adj8 controlled trial*).ab,kf,ti.
10. or/7-9
11. 6 and 10
12. (pragmatic adj5 RCT*).ab,kf,ti.
13. (pragmatic adj5 randomi#ed).ab,kf,ti.

14. (pragmatic adj5 controlled trial*).ab,kf,ti.
15. or/12-14
16. 6 and 15
17. 5 or 11 or 16
18. (meta analy* or metaanaly* or metanaly* or systematic review*).af.
19. 17 not 18
20. limit 19 to yr="2007 - 2018"
21. ((Cohort* and (random* or RCT)) or cmRCT).ti,ab,kw.
22. limit 21 to yr="2007 - 2018"
23. 20 use medall
24. 22 use clcmr
25. 23 or 24
26. remove duplicates from 25
27. 26 use medall
28. 26 use clcmr

**Searches for RCTs embedded in Electronic Health Records**

1. randomized controlled trial.pt.
2. controlled clinical trial.pt.
3. randomi?ed.ab.
4. placebo.ab.
5. randomly.ab.
6. clinical trials as topic.sh.
7. trial.ti.
8. or/1-7
9. exp animals/ not humans.sh.
10. 8 not 9
11. exp Electronic Health Records/
12. (EHR or electronic health record*).ab,kf,ti.
13. (EMR or electronic medical record*).ab,kf,ti.
14. (PHR or personal health record*).ab,kf,ti.
15. (EPR or electronic patient record*).ab,kf,ti.
16. exp Health Records, Personal/
17. or/11-16
18. 10 and 17
19. limit 18 to yr="2007 - 2018"
20. (Electronic health record or electronic health records or EHR).ti,ab,kw.
21. (Electronic medical record or electronic medical records or EMR).ti,ab,kw.
22. (Electronic patient record or electronic patient records or EPR).ti,ab,kw.
23. or/20-22
24. limit 23 to yr="2007 - 2018"
25. 19 use medall
26. 24 use clcmr
27. 25 or 26
28. remove duplicates from 27
29. 28 use medall
30. 28 use clcmr

**Searches for RCTs embedded in Administrative Databases**

1. randomized controlled trial.pt.
2. controlled clinical trial.pt.
3. randomi?ed.ab.
4. placebo.ab.
5. randomly.ab.
6. clinical trials as topic.sh.
7. trial.ti.
8. or/1-7
9. exp animals/ not humans.sh.
10. 8 not 9
11. administrative data*.ab,kf,ti.
12. healthcare data*.ab,kf,ti.
13. health care data*.ab,kf,ti.
14. or/11-13
15. 10 and 14
16. (administrative adj5 data*).ti,ab,kw.
17. health care data*.ti,ab,kw.
18. healthcare data*.ti,ab,kw.
19. or/16-18
20. (random* or RCT).ti,ab,kw.
21. 19 and 20
22. limit 15 to yr="2007 - 2018"
23. 22 use medall
24. limit 21 to yr="2007 - 2018"
25. 22 use clcmr
