# Supplementary material for: Methods and results used in the development of a consensus-driven extension to the Consolidated Standards of Reporting Trials (CONSORT) statement for trials conducted using cohorts and routinely collected data (CONSORT-ROUTINE)
Source: BMJ Open. 2021 Apr 29;11(4):e049093. doi: 10.1136/bmjopen-2021-049093 (PMC8094349; doi:10.1136/bmjopen-2021-049093)
Supplement: Author's manuscript [file bmjopen-2021-049093.draft_revisions.pdf]

BMJ Open is committed to open peer review. As part of this commitment we make the peer review history of every article we publish publicly available.

When an article is published we post the peer reviewers' comments and the authors' responses online. We also post the versions of the paper that were used during peer review. These are the versions that the peer review comments apply to.

The versions of the paper that follow are the versions that were submitted during the peer review process. They are not the versions of record or the final published versions. They should not be cited or distributed as the published version of this manuscript.

BMJ Open is an open access journal and the full, final, typeset and author-corrected version of record of the manuscript is available on our site with no access controls, subscription charges or pay-per-view fees (http://bmjopen.bmj.com).

If you have any questions on BMJ Open's open peer review process please email info.bmjopen@bmj.com

BMJ Open

Methods Used in the Development of a Consensus-driven Extension to the Consolidated Standards of Reporting Trials (CONSORT) Statement for Trials Conducted Using Cohorts and Routinely Collected Health Data

Journal:	*BMJ Open*
Manuscript ID	bmjopen-2021-049093
Article Type:	Original research
Date Submitted by the Author:	18-Jan-2021

Complete List of Authors:	Imran, Mahrukh; Lady Davis Institute for Medical Research, Psychiatry<br>Kwakkenbos, Linda; Lady Davis Institute for Medical Research, Psychiatry<br>McCall, Stephen; University of Oxford, National Perinatal Epidemiology Unit Clinical Trials Unit, Nuffield Department of Population Health; American University of Beirut, Center for Research on Population and Health, Faculty of Health Sciences<br>McCord, Kimberly; University of Basel, Basel Institute for Clinical Epidemiology and Biostatistics, Department of Clinical Research, University Hospital Basel<br>Frobert, Ole; Orebro University, Faculty of Health, Department of Cardiology<br>Hemkens, Lars; University of Basel, Basel Institute for Clinical Epidemiology and Biostatistics, Department of Clinical Research, University Hospital Basel<br>Zwarenstein, Merrick; Western University, Department of Family Medicine<br>Relton, Clare ; Queen Mary University of London, Institute of Population Health<br>Rice, Danielle; McGill University, ; Ottawa Hospital Research Institute,<br>Langan, Sinead; London School of Hygiene & Tropical Medicine, Faculty of Epidemiology and Population Health<br>Benchimol, Eric; Children's Hospital of Eastern Ontario, Gastroenterology, Hepatology and Nutrition<br>Thabane, Lehana ; McMaster University, Department of Health Research Methods, Evidence, and Impact<br>Campbell, Marion; University of Aberdeen, Health Services Research Unit<br>Sampson, Margaret; Children's Hospital of Eastern Ontario, Library Services<br>Erlinge, David; Lund University, Department of Cardiology, Clinical Sciences<br>Verkooijen, Helena; University of Utrecht Faculty of Medicine<br>Moher, David; Ottawa Hospital Research Institute, Ottawa Methods Centre<br>Boutron, Isabelle; INSERM<br>Ravaud, Philippe; INSERM<br>Nicholl, Jon; University of Sheffield, Medical Care Research Unit<br>Uher, Rudolf; Dalhousie University, Psychiatry

	Sauve, Maureen; Scleroderma Canada<br>Fletcher, John; BMJ<br>Torgerson, David; University of York, Health Sciences<br>Gale, Chris; Imperial College London, Medicine<br>Juszczak, Edmund; National Perinatal Epidemiology Unit Clinical Trials Unit, Nuffield Department of Population Health, University of Oxford<br>Thombs, Brett; Lady Davis Institute for Medical Research

Keywords:	STATISTICS & RESEARCH METHODS, GENERAL MEDICINE (see Internal Medicine), Clinical trials < THERAPEUTICS

SCHOLARONE™
Manuscripts

I, the Submitting Author has the right to grant and does grant on behalf of all authors of the Work (as defined in the below author licence), an exclusive licence and/or a non-exclusive licence for contributions from authors who are: i) UK Crown employees; ii) where BMJ has agreed a CC-BY licence shall apply, and/or iii) in accordance with the terms applicable for US Federal Government officers or employees acting as part of their official duties; on a worldwide, perpetual, irrevocable, royalty-free basis to BMJ Publishing Group Ltd ("BMJ") its licensees and where the relevant Journal is co-owned by BMJ to the co-owners of the Journal, to publish the Work in this journal and any other BMJ products and to exploit all rights, as set out in our licence.

The Submitting Author accepts and understands that any supply made under these terms is made by BMJ to the Submitting Author unless you are acting as an employee on behalf of your employer or a postgraduate student of an affiliated institution which is paying any applicable article publishing charge ("APC") for Open Access articles. Where the Submitting Author wishes to make the Work available on an Open Access basis (and intends to pay the relevant APC), the terms of reuse of such Open Access shall be governed by a Creative Commons licence – details of these licences and which Creative Commons licence will apply to this Work are set out in our licence referred to above.

Other than as permitted in any relevant BMJ Author's Self Archiving Policies, I confirm this Work has not been accepted for publication elsewhere, is not being considered for publication elsewhere and does not duplicate material already published. I confirm all authors consent to publication of this Work and authorise the granting of this licence.

For peer review only

Methods and Results Used in the Development of a Consensus-driven Extension to the Consolidated Standards of Reporting Trials (CONSORT) Statement for Trials Conducted Using Cohorts and Routinely Collected Data (CONSORT-ROUTINE)

Mahrukh Imran[1]; Linda Kwakkenbos[2]; Stephen McCall[3,4,5]; Kimberly A. Mc Cord[6]; Ole Fröbert[7]; Lars G. Hemkens[6]; Merrick Zwarenstein[8,9]; Clare Relton[10]; Danielle B. Rice[1,11]; Sinéad M. Langan[12]; Eric I. Benchimol[13,14,15]; Lehana Thabane[16]; Marion K. Campbell[17]; Margaret Sampson[18]; David Erlinge[19]; Helena M. Verkooijen[20,21]; David Moher[22]; Isabelle Boutron[23,24,25]; Philippe Ravaud[23,24,25]; Jon Nicholl[26]; Rudolf Uher[27]; Maureen Sauvé[28,29]; John Fletcher[,30]; David Torgerson[31]; Chris Gale[32]; Edmund Juszczak[3,33]; Brett D. Thombs[1,34]

[1]Lady Davis Institute for Medical Research, Jewish General Hospital, Montreal, Canada; [2]Behavioural Science Institute, Clinical Psychology, Radboud University, Nijmegen, the Netherlands; [3]National Perinatal Epidemiology Unit Clinical Trials Unit, Nuffield Department of Population Health, University of Oxford, Oxford, United Kingdom; [4]Center for Research on Population and Health, Faculty of Health Sciences, American University of Beirut, Ras Beirut, Lebanon; [5]Institute of Applied Health Sciences, School of Medicine, Medical Sciences and Nutrition, University of Aberdeen; [6]Basel Institute for Clinical Epidemiology and Biostatistics, Department of Clinical Research, University Hospital Basel, University of Basel, Basel, Switzerland; [7]Örebro University, Faculty of Health, Department of Cardiology, Örebro, Sweden; [8]Department of Family Medicine, Western University, London, Ontario, Canada; [9]IC/ES Western, London, Ontario, Canada; [10]Centre for Clinical Trials and Methodology, Barts Institute of Population Health Science, Queen Mary University, London, United Kingdom; [11]Department

For peer review only - http://bmjopen.bmj.com/site/about/guidelines.xhtml

of Psychology, McGill University, Montreal, Quebec, Canada; [12]Faculty of Epidemiology and Population Health, London School of Hygiene and Tropical Medicine, London, United Kingdom; [13]Department of Pediatrics and School of Epidemiology and Public Health, University of Ottawa, Ottawa, Canada; [14]ICES uOttawa, Ottawa, Ontario, Canada; [15]Children's Hospital of Eastern Ontario Research Institute, Ottawa, Ontario, Canada; [16]Department of Health Research Methods, Evidence, and Impact, McMaster University, Hamilton, Ontario, Canada; [17]Health Services Research Unit, University of Aberdeen, Aberdeen, United Kingdom; [18]Library Services, Children's Hospital of Eastern Ontario, Ottawa, Canada; [19]Department of Cardiology, Clinical Sciences, Lund University, Lund, Sweden; [20]University Medical Center Utrecht, Utrecht, the Netherlands; [21]University of Utrecht, Utrecht, the Netherlands; [22]Centre for Journalology, Clinical Epidemiology Program, Ottawa Hospital Research Institute, Ottawa, Ontario, Canada; [23]INSERM, Paris, France; [24]Centre d'Épidémiologie Clinique, Hôpital Hôtel Dieu, Assistance Publique–Hôpitaux de Paris, Paris, France; [25]Faculté de Médecine, Université Paris Descartes, Sorbonne Paris Cité, Paris, France; [26]School of Health and Related Research, University of Sheffield, Sheffield, United Kingdom; [27]Department of Psychiatry, Dalhousie University, Halifax, Canada; [28]Scleroderma Society of Ontario, Hamilton, Ontario, Canada; [29]Scleroderma Canada, Hamilton, Ontario, Canada; [30]British Medical Journal, London, United Kingdom; [31]York Trials Unit, Department of Health Sciences, University of York, York, United Kingdom; [32]Neonatal Medicine, School of Public Health, Faculty of Medicine, Imperial College London, Chelsea and Westminster campus, London, United Kingdom; [33]Nottingham Clinical Trials Unit, University of Nottingham, Nottingham, United Kingdom.

For peer review only

For peer review only - http://bmjopen.bmj.com/site/about/guidelines.xhtml

[34]Departments of Psychiatry; Epidemiology, Biostatistics and Occupational Health; Medicine; and Educational and Counselling Psychology; and Biomedical Ethics Unit, McGill University, Montreal, Quebec, Canada.

Address for Correspondence: Brett D. Thombs, PhD; Jewish General Hospital; 4333 Cote Ste Catherine Road; Montreal, Quebec H3T 1E4; Tel (514) 340-8222 ext. 25112; E-mail: brett.thombs@mcgill.ca

For peer review only

ABSTRACT

Objectives: Randomised controlled trials (RCTs) conducted using cohorts and routinely collected data, including registries, electronic health records and administrative databases, are increasingly used in health care intervention research. A Consolidated Standards of Reporting Trials (CONSORT) statement extension for trials conducted using cohorts and routinely collected data (CONSORT-ROUTINE) has been developed with the goal of improving reporting quality. This article describes the processes and methods used to develop the extension and decisions made to arrive at the final checklist.

Methods: The development process involved 5 stages: (1) identification of the need for a reporting guideline and project launch; (2) conduct of a scoping review to identify possible modifications to CONSORT 2010 checklist items and possible new extension items; (3) a 3-round modified Delphi Study involving key stakeholders to gather feedback on the checklist; (4) a consensus meeting to finalise items to be included in the extension, followed by stakeholder piloting of the checklist; and (5) publication, dissemination and implementation of the final checklist.

Results: 27 items were initially developed and rated in Delphi Round 1, 13 items were rated in Round 2 and 11 items were rated in Round 3. Response rates for the Delphi Study were 92 of 125 (74%) invited participants in Round 1, 77 of 92 (84%) Round 1 completers in Round 2, and 62 of 77 (81%) Round 2 completers in Round 3. Twenty-seven members of the project team representing a variety of stakeholder groups attended the in-person consensus meeting. The final checklist includes 5 new items and 8 modified items. The extension Explanation & Elaboration document further clarifies aspects that are important to report.

For peer review only - http://bmjopen.bmj.com/site/about/guidelines.xhtml

Conclusion: Uptake of CONSORT-ROUTINE and accompanying Explanation & Elaboration document will improve conduct of trials, as well as the transparency and completeness of reporting of trials conducted using cohorts and routinely collected data.

Keywords: administrative data, cohort, CONSORT, electronic health records, electronic medical records, registries, reporting guideline, routinely collected data

For peer review only

For peer review only - http://bmjopen.bmj.com/site/about/guidelines.xhtml

Strengths and Limitations of this study

- We followed a 5-step process to develop CONSORT-ROUTINE, consistent with EQUATOR guidance.
- Items were informed by reporting guidelines on similar research designs, a scoping review, a 3-round Delphi process, and expert members of the guideline development team.
- CONSORT-ROUTINE was reviewed and tested at various stages of the development by project team members and key stakeholders.
- The limited methodological literature on trials conducted using cohorts and routinely collected data was a limitation in developing the extension.
- Similar to other reporting guidelines, CONSORT-ROUTINE will require re-evaluation and revisions over time to ensure that it is kept up to date with evolving methodology and practice of trials using cohorts and routinely collected data.

For peer review only - http://bmjopen.bmj.com/site/about/guidelines.xhtml

BACKGROUND

The use of reporting guidelines, including the Consolidated Standards of Reporting Trials (CONSORT) statement, improves the transparency and completeness of reports of results from randomised controlled trials (RCTs).[1-4] The CONSORT statement helps to facilitate critical appraisal and interpretation of RCTs by providing guidance to authors on a minimal set of items that should be reported for all trials.[5] The CONSORT 2010 guideline aimed to improve the reporting of two-arm parallel group RCTs. Extensions of the CONSORT statement have been developed to encourage better reporting of other trial designs, including, for instance, multi-arm parallel group randomised trials, cluster trials, pilot and feasibility trials, and pragmatic trials.[6-9]

There is a growing interest in RCTs conducted using cohorts or routinely collected data, including registries, electronic health records (EHRs), and administrative databases.[10-14] In a cohort, a group of individuals is gathered for the purpose of conducting research, whereas routinely collected data refer to data initially collected for purposes other than research or without specific a priori research questions developed before collection.[15,16] Trials may use a cohort or routinely collected data for (1) identification of eligible participants, (2) outcome ascertainment, (3) to implement an intervention, or for a combination of these purposes. For example, in registry-based RCTs, a registry could be used to identify eligible participants for a trial, for the collection of participant baseline characteristics, and as the source of outcome data; some registries have used interactive technology to actively flag participants for RCT enrollment as patient data are entered into the registry.[12] In some EHR trials, the EHR itself is used to implement an intervention. For example, one RCT tested an intervention to reduce antibiotic prescribing by feeding back personalized antibiotic prescription data to primary care physicians.[17]

For peer review only - http://bmjopen.bmj.com/site/about/guidelines.xhtml

The use of cohorts and routinely collected data may make RCTs easier and more feasible to perform by reducing cost, time and other resources.[18,19] It may also facilitate the conduct of trials that more closely replicate real-world clinical practice. These trial designs, however, are relatively recent innovations, and published RCT reports may not describe important aspects of their methodology in a standardised way. Trials conducted using cohorts and routinely collected data share certain elements with conventional RCTs, but there are also distinctive elements to report which are not covered in the CONSORT 2010 statement. The REporting of studies Conducted using Observational Routinely-collected Data (RECORD) statement provides guidance on reporting of studies conducted using routinely collected data but does not address RCT-specific methodological and reporting considerations.[20] Research conducted using routinely collected data presents unique methodological challenges that are often insufficiently reported, but there is scant guidance on methods and reporting of trials conducted using routinely collected data or cohorts.[21,22]

An extension to the CONSORT statement for RCTs conducted using cohorts and routinely collected data was developed using methods recommended for developing reporting guidelines.[23] This article describes, in detail, the consensus-based development process. The main aims of this article are to: (1) describe the methods and processes used in the development of the CONSORT Extension for Trials Conducted Using Cohorts and Routinely Collected Data (CONSORT-ROUTINE; Kwakkenbos et al., under review) and (2) describe decisions made to arrive at the final checklist and the accompanying Explanation and Elaboration statement.

METHODS

The project was registered with the Enhancing the QUAlity and Transparency Of health Research (EQUATOR) network.[24] We followed the EQUATOR network's guidelines for

For peer review only - http://bmjopen.bmj.com/site/about/guidelines.xhtml

recommended methods and processes for developing, disseminating, and implementing health care reporting guidelines.[23] These methods have been used in the development of other similar EQUATOR guidelines. Figure 1 illustrates the 5 parts of the development process for this guideline:

Project Phase 1: Project Launch, Establishment of Team, and Funding

Need for the guideline and literature review: An initial informal review of reports of published protocols and reports of trials using cohorts and routinely collected data by BDT and LK suggested that there appeared to be deficiencies in reporting of such trials. For instance, many reports did not adequately describe the cohort or database from which trial participants were recruited, processes used to link participants across databases were not always provided, and it was sometimes unclear whether trial outcomes were assessed by the trialists or ascertained via existing databases used to conduct the trial. A review of the EQUATOR website and published literature indicated that there was no existing reporting guideline for these types of trials. The RECORD statement addresses reporting issues related to routinely collected data but does not include guidance on reporting of trials. Many trials conducted using routinely collected data are pragmatic or use cluster designs, for instance, but CONSORT extensions for those types of trials do not address issues germane to the use of cohorts or routinely collected data to conduct trials.[7,9]

Project launch and identification of CONSORT-ROUTINE project members: Initial discussions on developing a CONSORT extension for RCTs conducted using cohorts occurred in November 2016 at the Trials within Cohorts symposium in London, United Kingdom (LK, MZ, CR, BDT).[25] Discussions continued virtually and key people involved in cohort-embedded trials or the EQUATOR network were approached during December 2016 (HMV, DM, IB, PR, JN,

For peer review only - http://bmjopen.bmj.com/site/about/guidelines.xhtml

RU, DT). It was suggested that trials conducted in registries had many characteristics similar to those in cohorts, and there was agreement to include registry-based trials in the extension. People with expertise in registry-based trials were approached in March 2017 (OF, LT, MKC, DE), and an experienced librarian (MSampson) and patient representative familiar with trials conducted using cohorts (MSauvé) were also included in the group at that point.

The project was registered on the EQUATOR website in April 2017. During the preparatory phase, while developing searches and reviewing example publications, we became aware that trials conducted using EHRs and administrative databases also shared similar characteristics with trials in cohorts and registries, and it was decided to expand the scope to trials conducted using cohorts and routinely collected data. In July 2017, trialists, who were leading the development of a reporting guideline for EHRs, joined the project group (EJ, CG). Given the relevance of their previous work and their expertise (LGH, SML, DM, EIB) authors who had been involved in the development of the RECORD statement were invited to join the team.[20] Several doctoral students also joined the project team (SM, KAM, and DBR). A steering committee comprising of 10 members with key expertise for consultation was established. A research coordinator (MI) was hired in April 2018 to manage the project, and an experienced journal editor was invited to join (JF). The group communicated regularly throughout the process via a number of virtual meetings, using an online platform to conduct teleconferences, as well as through email discussions.

Rationale for developing one checklist versus 4 different checklists for trials conducted using cohorts, registries, EHRs, and administrative databases: Team members discussed the advantages and disadvantages of creating individual checklists for each of the 4 types of data versus a single checklist for all 4. It was determined that, although there are some differences in

For peer review only - http://bmjopen.bmj.com/site/about/guidelines.xhtml

the implementation of trials across the different types of data sources, the methodological principles are similar, and there is substantial overlap in the design, conduct and factors that may influence interpretability. Thus, the steering committee reached consensus to develop a single statement, addressing any differences by including "if applicable" to items in the checklist that may not apply to all trial designs, and to clarify differences in the Explanation & Elaboration publication as deemed necessary.

Funding: The project team obtained its main source of funding from a grant from the Canadian Institutes of Health Research Institutes (CIHR) to support the development of the guideline (BDT, OF, EJ, LK, CR; Grant #PJT-156172). EJ and CG also obtained funding from the United Kingdom National Institute of Health Research Clinical Trials Unit Support Funding - Supporting efficient / innovative delivery of NIHR research. In addition, funding to hold the face-to-face meeting was provided by a Planning and Dissemination Grant from CIHR (BDT, LK; Grant #PCS - 161863) and by contributions from Queen Mary University of London, the University of Sheffield, McGill University, and the Lady Davis Institute for Medical Research of the Jewish General Hospital in Montreal, Canada.

A project protocol was developed and published.[22]

Project Phase 2: Scoping Review

A preliminary "long list" of possible reporting items was formulated by LK and KAM based on review of the CONSORT 2010 statement items, the Strengthening the Reporting of Observational Studies in Epidemiology (STROBE)[26] and the RECORD statements,[20] as well as discussions with steering committee members. The STROBE and RECORD statements were considered the most relevant to this project because of their focus on reporting of observational studies and non-interventional studies using routinely collected data.

For peer review only - http://bmjopen.bmj.com/site/about/guidelines.xhtml

A scoping review was conducted to identify: (1) articles on the methodology or reporting of RCTs conducted using cohorts or routinely collected data that could inform the development of new items or modification of existing CONSORT items; (2) trial reports to identify aspects of reporting that need improvement and examples of good reporting of potential checklist items that could be used to support CONSORT-ROUTINE.[27] We searched for relevant articles on trials conducted using cohorts, registries, EHRs, and administrative databases from 2007 to 2018. After screening articles for inclusion and exclusion at the abstract and full-text level, 10 people from the team independently reviewed the included papers and provided suggestions for modifications or additional reporting guideline items until no new ideas emerged (saturation). Suggestions were added in a standardized, shared spreadsheet. At the same time, team members provided examples of good reporting for each proposed item or item modification. Additionally, the review helped us to create a list of authors with experience in these trial designs as potential participants for the Delphi study. Search terms used in the scoping review are shown in Supplementary File 1.

Project Phase 3: Delphi Study

The objectives of our Delphi study were (a) to obtain feedback on the importance of including each candidate item in CONSORT-ROUTINE; (b) to improve the wording of items considered important; and (c) to elicit suggestions for additional items not in the existing list. We aimed to engage key stakeholders across different sectors and backgrounds. There are not fixed guidelines on the sample size of Delphi studies, and the ideal number of participants may depend on the complexity of the topic, the likely heterogeneity of relevant experiences and viewpoints, and resources available to manage the data generated.[28-30] Many studies use small groups of experts (e.g., < 20), but we believed that a larger group with diverse expertise would best

For peer review only - http://bmjopen.bmj.com/site/about/guidelines.xhtml

complement the knowledge of the project team. Thus, we sent out an invitation to reporting guideline developers (including those involved in previous CONSORT extensions), funders, journal editors, patient representatives, trial methodologists, epidemiologists, meta-research authors, ethicists, biostatisticians and clinical trialists who were identified by members of the project team. We also encouraged recipients of the invitation to forward the invitation to other potentially interested stakeholders.

The Delphi surveys were built and hosted using an online survey platform in Qualtrics®. During registration, we gathered demographic and professional background characteristics of participants, including geographical location, self-identified stakeholder group (e.g., clinical trials user, clinical trialist, methodologist), employment sector, years of experience in trials research, and research experience in trials conducted using cohorts or routinely collected data.

Registered participants received a link to access each of the 3 rounds of the Delphi survey. In each round, we asked participants to rate their perceptions about the importance of each suggested reporting item by ranking items based on how essential they are for reporting on a 1-5 Likert scale (1 = not essential; 5 = essential). There is not consensus on the ideal number of Likert categories or groupings for decision-making, but it is common to use between 4-point and 7-point scales.[29]

Responses were categorized as follows:

1 to 2 = low score (item should not be part of CONSORT-ROUTINE checklist);

3 = moderate (item should be discussed);

4 to 5 = high score (item should be part of CONSORT-ROUTINE checklist).

For peer review only - http://bmjopen.bmj.com/site/about/guidelines.xhtml

Participants also had the option to select "Not my expertise" for items if they believed that they did not have the appropriate level of expertise to rate an item. Figure 2 shows a screenshot of an example proposed modification item from the survey:

Items from the CONSORT 2010 statement for which modifications were initially not proposed were also included in the survey so that participants could provide comments or make recommendations for modifications to these items. For all items (proposed modifications and CONSORT 2010 items), we provided participants with the opportunity to give open-ended feedback, using free-text boxes provided at the bottom of each survey page and at the end of the survey. At the end of the survey, participants were asked to provide any additional items that they believed would be important for reporting in trials conducted using cohorts and routinely collected data, but which had not been included in the proposed set of new and modified items.

We launched Round 1 of the survey on February 4, 2019 with 2 weeks to provide responses. Round 2 was launched on March 4, 2019, and Round 3 was launched on April 1, 2019. After each round, the Qualtrics built-in analysis software was used to generate a distribution of scores and to aggregate group results for each item (mean score, maximum and minimum score, standard deviation, variance, percentage ratings of 1-5 ranking for items) and summary statistics were circulated amongst all participants. Individual responses were not fed back. In addition, a bar chart with the ratings and counts for each item was created. Following each round of the survey, the CONSORT-ROUTINE steering committee members reviewed the survey results independently and then met via teleconference to discuss and analyze the results of the survey. During these meetings, decisions were made on how to address comments from

For peer review only - http://bmjopen.bmj.com/site/about/guidelines.xhtml

participants by modifying, adding, or combining items. Notes were also made on comments that reflected a need for explanation in the Explanation & Elaboration companion to the checklist.

We predefined consensus as at least 2/3 of responders rating the importance of an item as 'high' or 'very high'. Items that reached consensus for inclusion were not rated again in the next round. For some items that did not reach consensus, the wording of several items was revised based on participants' suggestions. Items that did not reach consensus were rated again in the next round in their original or revised form. Reports summarizing the Delphi results were circulated after each round including summary statistics such as counts, means, standard deviations and variances for the responses on each item. Reminder emails were sent one week prior to the deadline and extensions were provided if requested for all 3 rounds in order to maximize participation.

Since the Delphi Study was advisory, all items were reviewed and vetted again at the in-person consensus meeting, and comments provided by participants of the Delphi Study were taken into consideration while making decisions to include or exclude items.

Project Phase 4: In-person Consensus Meeting and Development of Checklist Publication

A two-day in-person consensus meeting was held on May 13-14, 2019 in London, United Kingdom. The purpose of the meeting was to discuss the Delphi results, make decisions on items to retain in the final checklist, make any necessary modifications to items, and suggest reporting aspects that should be addressed in the Explanation & Elaboration documentation supporting the checklist. The meeting was attended by 26 members of the CONSORT-ROUTINE Group.

We used approaches similar to those used in previous consensus meetings for other guidelines. Participants were provided with the results of the initial long-list generation and the Delphi study in advance of the meeting. At the meeting, steering committee members first

For peer review only - http://bmjopen.bmj.com/site/about/guidelines.xhtml

presented the background and an update on work done to date, in order to facilitate the discussions. Session chairs then separately presented items from the preliminary checklist, results of the Delphi study, and feedback from stakeholders, after which the group discussed in an open forum. Decisions were made on items to be modified or added based on the following criteria (1) whether they addressed elements unique to trials conducted using cohorts or routinely collected data versus elements applicable to any trial, and (2) whether they reflected information that should be included in a minimum reporting set of items. Notes were taken and the discussions were audio-recorded to ensure that the content was accurately captured.

Following the consensus meeting, refinement of the content and wording of the items was continued through online group discussions with CONSORT-ROUTINE project team members. The initial version of the checklist was pilot-tested by circulating it among stakeholders in order to assess its usability and to identify any challenges which might arise while applying the checklist. Pilot-testing the checklist also provided insight into issues that should be addressed in detail in the Explanation and Elaboration statement.

Project Phase 5: Publication, Dissemination and Implementation

As with several previous CONSORT extensions, it was decided to publish the reporting checklist with a detailed Explanation and Elaboration statement in the same document.[6-9] The Explanation and Elaboration statement is intended to provide an in-depth explanation of the scientific rationale for each recommendation, together with an example of clear reporting for each item.

In addition to publication of the reporting guideline checklist and Explanation & Elaboration material, to attempt to maximize uptake, we will undertake additional dissemination activities, including presentations and workshops at conferences and other venues. We also plan

For peer review only - http://bmjopen.bmj.com/site/about/guidelines.xhtml

to seek endorsement of the guideline by journal editors. Research has shown that formal endorsement and adoption of the CONSORT statement by journals is associated with improved quality of reporting.[2] Studies conducted by members of our team have benchmarked pre-extension reporting completeness in trials conducted in cohorts, registries, EHRs, and administrative databases.[31-33] There were not enough examples of completed cohort-embedded trials for benchmarking reporting.

The final CONSORT-ROUTINE checklist (Kwakkenbos et al., under review) has been published at: [Insert Link – SEE SUPPLEMENTAL MATERIAL]

Patient and public involvement: One of the members of our CONSORT-ROUTINE team, Maureen Sauvé, is a patient organisation leader. She has been involved in working with researchers to establish a cohort of patients living with the rare disease scleroderma, which supports RCTs of trials of online rehabilitation, self-management and psychological intervention programmes.

RESULTS

Stage 2: Scoping review and initial long list of potential items

The scoping review sought methods articles and reports of trials conducted using cohorts, registries, EHRs, or administrative databases.

Cohorts: The database search identified 1,185 publications, of which 1,062 were excluded after title and abstract screening and 37 after full-text review. A total of 86 studies were included in the scoping review, including 15 papers on methodological considerations of using cohorts for conducting RCTs. All trials used the cohort for both identification of patients and outcome ascertainment.

For peer review only - http://bmjopen.bmj.com/site/about/guidelines.xhtml

Registries: The search identified 234 publications, of which 143 received full-text review. A total of 106 publications were eligible, including 95 trial reports or protocols (both identification of patients and outcome ascertainment (n = 27); identification of patients only (n = 28); outcome ascertainment only (n= 40)) and 11 papers on methodological considerations.

EHRs: The search identified 2,085 citations, of which 548 studies were reviewed at the full-text level. A total of 289 eligible publications, including 263 trial protocols or reports (both identification of patients and outcome ascertainment (n= 169); identification of patients only (n = 38); outcome ascertainment only (n = 56)) and 26 articles that described methodological considerations.

Administrative databases: The search identified 663 citations, of which 151 full texts were reviewed. There were a total of 117 trial protocols or reports included (both identification of patients and outcome ascertainment (n = 57); identification of patients only (n= 1); outcome ascertainment only (n = 58)) and 1 paper on methodological considerations.

Delphi Study Results

Of 125 people invited to take part in the Delphi study, 115 people registered via an online survey, and 92 (74%) provided responses on the items in Round 1. Figures 3 and 4 present the types of stakeholder groups that completed Round 1 of the Delphi Study and the type of trials conducted using cohorts or routinely collected databases with which they had familiarity. Participants belonging to more than one category had the option of checking multiple options in the survey.

Round 1: Of the 92 participants who completed the Round 1 survey, out of which 90 provided valid ratings and 2 provided comments but not ratings. Of the 27 items rated in Round 1, 14 reached consensus to be included in discussions at the consensus meeting; the other 13 did

For peer review only - http://bmjopen.bmj.com/site/about/guidelines.xhtml

not reach consensus and were included in Round 2. Based on Round 1 feedback, a total of 11 items were modified for review in Round 2, including 2 items that were combined into one. No items were excluded from the checklist.

Round 2: Of the 92 participants who completed Round 1, 77 (84%) completed the Round 2 survey. Of the 13 items rated, 2 reached consensus for inclusion in consensus meeting discussions, and 11 did not reach consensus in Round 2. Based on Round 2 feedback, 8 items were modified prior to Round 3.

Round 3: Of the 77 people who completed Round 2, 62 (81%) completed Round 3. Of the 11 items in Round, 5 items reached consensus in Round 3. The remaining 6 items did not reach consensus after the 3 rounds.

There were several new items suggested via the Delphi process but not added to the potential item list. The main reasons why some items were suggested but not incorporated were:

(a) The suggestion was encapsulated in CONSORT 2010 items, was already captured by proposed new or modified items, or could be captured by further modifying new or modified items;

(b) The suggestion was not specific to trials conducted using cohorts and routinely collected data and, thus, was recommending a change to the CONSORT 2010 checklist, which was not the task of the CONSORT-ROUTINE group.

Summary results of the 3 rounds can be accessed at: https://osf.io/4zh6f/

In-person Consensus Meeting

Table 1 summarises the CONSORT-ROUTINE group's discussions and advisory decisions for each of the items that was discussed during the in-person meeting. If there were differing opinions on the inclusion or exclusion of items and consensus could not be reached, voting was

For peer review only - http://bmjopen.bmj.com/site/about/guidelines.xhtml

implemented by the session chair, with an 80% threshold for inclusion in the checklist as part of the minimal set of recommended reporting items. The key recommendations that emerged were as follows:

- Proposed modification to CONSORT 2010 items: It was recommended to retain proposed modifications to 7 CONSORT 2010 items. These modifications pertained to differences in mechanisms used to conduct trials using cohorts or routinely collected databases. As in previous CONSORT extensions, some of the recommended changes end with "if applicable" to show that some information which authors are being asked to report might not be relevant or applicable for their particular RCT, or the particular type of data that was used in the RCT.
- Proposed additional items: consensus was reached to include 6 additional items and to add a new subheading, "Cohort or routinely collected database" to the checklist.

A recurrent discussion point was the need to minimise adding new items to the abstract unless they are essential due to word limits imposed by journals. A suggestion was made to expand the explanatory text of the Explanation & Elaboration document for nine unchanged CONSORT 2010 items to clarify additional requirements for reporting aspects of the trial without modifying the item: item 1a (identification as a randomised trial in the title), item 4b (settings and location where the data were collected), item 5 (intervention), item 13b (losses and exclusions after randomisation), item 14a (dates of recruitment/follow-up), item 15 (baseline data), item 20 (limitations), item 21 (generalisability) and item 24 (study protocol). For the abstract, there was an agreement to include an additional item to the abstract for naming the cohort or routinely collected database (item 1c). This item was later merged with item 1b from

For peer review only - http://bmjopen.bmj.com/site/about/guidelines.xhtml

the CONSORT 2010 checklist after discussion with the project team (Table 1). Thus, the final extension checklist included 8 modified items and 5 new items.[23]

CONSORT-ROUTINE Pilot-test

The preliminary version of the checklist was pilot-tested by 17 people who had been previously involved in conducting trials using cohorts and routinely collected data. Based on feedback received from the pilot-test there were minor modifications made to the wording of 2 items for clarity (Item 1b and Item 9) in the final checklist.[23]

DISCUSSION

We have developed a consensus-driven extension to the CONSORT 2010 Statement for RCTs conducted using cohorts and routinely collected data (Kwakkenbos et al., under review). CONSORT-ROUTINE contains minimum reporting requirements with appropriate flexibility as described in the Explanation & Elaboration part of our checklist document. This article described how we reached the final checklist and Explanation & Elaboration text and provides information on the decision-making process. We anticipate this paper will help others who may learn from our experiences and may apply this to the development of future guidelines or extensions.

There were several important strengths to our approach. A consensus-driven Delphi methodology, which is recommended when developing health care reporting guidelines by the EQUATOR network, was used to develop the extension.[23] We engaged with key stakeholders in trials research and potential end-users of the resultant CONSORT-ROUTINE reporting guideline throughout the development process. The process involved participants from a wide range of scientific disciplines and with diverse experience in conducting trials using different cohorts and routinely collected databases. As with other CONSORT-related guidelines, the inclusion of CONSORT Group members (IB, DM, PR) was intended to ensure consistency in the use of

For peer review only - http://bmjopen.bmj.com/site/about/guidelines.xhtml

recommended methods in the development, dissemination, and implementation of the extension. We recorded high response rates of 74% (92 respondents), 84% (77 respondents) and 81% (62 respondents) in Delphi rounds 1, 2, and 3, respectively. In addition, the number of registered participants and responders is larger than in most Delphi surveys used to develop health care reporting guidelines.[8,34,35] Finally, we achieved a high degree of consensus that was consistent across Delphi survey rounds for the majority of the items.

There are also limitations to consider. One is that most participants were academic researchers with primary roles in trials research, and, despite our broad engagement efforts, the number of participants from some stakeholder groups was small. One patient was included as a member of the reporting guideline development team, but no patients participated in the Delphi exercise. It is possible that perceptions about the importance of items might have differed across different stakeholder groups which might have favoured the inclusion or exclusion of certain items. Nonetheless, our project group included people from diverse backgrounds with expertise in using different types of data sources, who oversaw the development process to ensure that the checklist was equally applicable to, and representative of, all 4 types of data sources. A second is that our scoping review was not designed to capture each and every trial conducted using routinely collected data. This was in part because of the lack of accepted specific Medical Subject Headings terms to identify these studies, or any research using routinely collected data, and the limited number of completed trials and methodological articles on these trial designs. For our purposes it was not necessary to capture all trials that had been conducted using cohorts or routinely collected data, and we believe that we were able to capture a significant number of important trial reports and methodology papers that served as a basis for the development of our extension. A third is that the CONSORT-ROUTINE group predominantly consisted of members

For peer review only - http://bmjopen.bmj.com/site/about/guidelines.xhtml

from high-income countries, which might have led to decreased applicability of the checklist for trials conducted in other settings. Finally, as with all reporting guidelines, ours will require re-evaluation and revisions over time to ensure that it is kept up to date with evolving research and knowledge on these trail designs.

CONCLUSION

CONSORT-ROUTINE has now been developed and can be used to support comprehensive reporting of RCTs conducted using cohorts or routinely collected data. The extension statement contains minimum requirements of reporting that we encourage researchers to report. A baseline assessment of the completeness and reporting of these trial designs is being conducted, and the impact of the extension will be assessed in the coming years. While we anticipate that CONSORT-ROUTINE may need to be updated with the evolution of research methods, we hope the guideline will improve the reporting of RCTs conducted using cohorts and routinely collected data, enhance their interpretability and credibility of their results, improve their reproducibility, indirectly facilitate their robust design and conduct and lead to improved patient care.

For peer review only

For peer review only - http://bmjopen.bmj.com/site/about/guidelines.xhtml

Contributors: MI, LK, OF, LGH, MZ, CR, SML, DM, MS, CG, EJ and BDT were involved in initial phases of study conception, design of the search strategy and development of conceptual frameworks. SM, KAMcC, DBR, EIB, LT, MKC, DE, HMV, IB, PR, JN, RU, MS, JF and DT provided regular feedback on each of these steps. MI wrote the first draft with LK and BDT. All authors made provided critical revisions to the development of this manuscript and approved the final version.

Funding: The development of CONSORT-ROUTINE was funded by an operating grant from CIHR (PI BDT, Co-PIs OF, EJ, LK, CR; Grant #PJT156172), a CIHR planning and dissemination grant (#PCS – 161863), and a grant from the United Kingdom National Institute of Health Research (NIHR) Clinical Trials Unit Support Funding - Supporting efficient / innovative delivery of NIHR research (PI Juszczak, Co-PI Gale). BDT is supported by a Fonds de recherche du Québec - Santé researcher salary award and a Tier 1 Canada Research Chair. CG is supported by the United Kingdom Medical Research Council through a Clinician Scientist Fellowship. SML is supported by a Wellcome Senior Clinical Fellowship in Science (205039/Z/16/Z). RU is supported by the Canada Research Chairs Program (Award #231397). EIB was supported by a New Investigator Award from CIHR, the Canadian Association of Gastroenterology and Crohn's and Colitis Canada, and the Career Enhancement Program of the Canadian Child Health Clinician Scientist Program. DBR is supported by a Vanier CIHR Graduate Scholarship.

Disclaimer: The views expressed are those of the authors and not necessarily those of the NHS, the NIHR or the Department of Health and Social Care.

Competing interests: The authors have read and understood the BMJ policy on declaration of interests and declare that they have no competing interests.

For peer review only - http://bmjopen.bmj.com/site/about/guidelines.xhtml

Patient consent: Not required.

Ethics approval: This study does not require ethics approval.

Data Sharing: No additional data were collected beyond those reported.

For peer review only

For peer review only - http://bmjopen.bmj.com/site/about/guidelines.xhtml

FIGURE LEGENDS

Figure 1: Development process of the CONSORT Extension for Trials Conducted Using Cohorts and Routinely Collected Data (CONSORT-ROUTINE)

Figure 2: Example of a round 1 Delphi survey item as presented in the online survey.

Figure 3: Professional roles reported by participants who completed Round 1 of the CONSORT-ROUTINE Delphi Study (%). Participants could report more than one role.

Figure 4: Participants of Round 1 of the CONSORT-ROUTINE Delphi Study by type of cohort or routinely collected database with which they had familiarity (%). Participants could report more than one.

For peer review only

For peer review only - http://bmjopen.bmj.com/site/about/guidelines.xhtml

REFERENCES

1. Altman DG. Transparent reporting of trials is essential. *Am J Gastroenterol*. 2013;108(8):1231-1235.

2. Turner L, Shamseer L, Altman DG, et al. Consolidated standards of reporting trials (CONSORT) and the completeness of reporting of randomised controlled trials (RCTs) published in medical journals. *Cochrane Database Syst Rev*. 2012;11:MR000030.

3. Cobo E, Cortés J, Ribera JM, et al. Effect of using reporting guidelines during peer review on quality of final manuscripts submitted to a biomedical journal: masked randomised trial. *BMJ*. 2011;343:d6783.

4. Moher D, Jones A, Lepage L; CONSORT Group (Consolidated Standards for Reporting of Trials). Use of the CONSORT statement and quality of reports of randomized trials: a comparative before-and-after evaluation. *JAMA*. 2001;285(15):1992-1995.

5. Schulz KF, Altman DG, Moher D; CONSORT Group. CONSORT 2010 statement: updated guidelines for reporting parallel group randomised trials. *BMJ*. 2010;340:c332.

6. Juszczak E, Altman DG, Hopewell S, Schulz K. Reporting of Multi-Arm Parallel-Group Randomized Trials: Extension of the CONSORT 2010 Statement. *JAMA*. 2019;321(16):1610-1620.

7. Campbell MK, Elbourne DR, Altman DG; CONSORT group. CONSORT statement: extension to cluster randomised trials. *BMJ*. 2004;328(7441):702-708.

8. Eldridge SM, Chan CL, Campbell MJ, et al. CONSORT 2010 statement: extension to randomised pilot and feasibility trials. *BMJ*. 2016;355:i5239.

9. Zwarenstein M, Treweek S, Gagnier JJ, et al. Improving the reporting of pragmatic trials: an extension of the CONSORT statement. *BMJ*. 2008;337:a2390.

For peer review only - http://bmjopen.bmj.com/site/about/guidelines.xhtml

10. Anderson GL, Burns CJ, Larsen J, Shaw PA. Use of administrative data to increase the practicality of clinical trials: Insights from the Women's Health Initiative. *Clin Trials*. 2016;13(5):519-526.

11. James S, Fröbert O, Lagerqvist B. Cardiovascular registries: a novel platform for randomised clinical trials. *Heart*. 2012;98(18):1329-1331.

12. James S, Rao SV, Granger CB. Registry-based randomized clinical trials--a new clinical trial paradigm. *Nat Rev Cardiol*. 2015;12(5):312-316.

13. Relton C, Torgerson D, O'Cathain A, Nicholl J. Rethinking pragmatic randomised controlled trials: introducing the "cohort multiple randomised controlled trial" design. *BMJ*. 2010;340:c1066.

14. van Staa TP, Dyson L, McCann G, et al. The opportunities and challenges of pragmatic point-of-care randomised trials using routinely collected electronic records: evaluations of two exemplar trials. *Health Technol Assess*. 2014;18(43):1-146.

15. Morrato EH, Elias M, Gericke CA. Using population-based routine data for evidence-based health policy decisions: lessons from three examples of setting and evaluating national health policy in Australia, the UK and the USA. *J Public Health*. 2007;29(4):463-471.

16. Moskowitz A, Chen KP, Cooper AZ, Chahin A, Ghassemi MM, Celi LA. Management of Atrial Fibrillation with Rapid Ventricular Response in the Intensive Care Unit: A Secondary Analysis of Electronic Health Record Data. *Shock*. 2017;48(4):436-440.

17. Hemkens LG, Saccilotto R, Reyes SL, et al. Personalized prescription feedback to reduce antibiotic overuse in primary care: rationale and design of a nationwide pragmatic randomized trial. *BMC Infect Dis*. 2016;16:421.

For peer review only - http://bmjopen.bmj.com/site/about/guidelines.xhtml

18. Mc Cord KA, Ewald H, Ladanie A, et al. Current use and costs of electronic health records for clinical trial research: a descriptive study. *CMAJ Open*. 2019;7(1):E23-E32.

19. Wachtell K, Lagerqvist B, Olivecrona GK, James SK, Fröbert O. Novel Trial Designs: Lessons Learned from Thrombus Aspiration During ST-Segment Elevation Myocardial Infarction in Scandinavia (TASTE) Trial. *Curr Cardiol Rep*. 2016;18(1):11.

20. Benchimol EI, Smeeth L, Guttmann A, et al. The REporting of studies Conducted using Observational Routinely-collected health Data (RECORD) statement. *PLoS Med*. 2015;12(10):e1001885.

21. Hemkens LG, Benchimol EI, Langan SM, et al. The reporting of studies using routinely collected health data was often insufficient. *J Clin Epidemiol*. 2016;79:104-111.

22. Kwakkenbos L, Juszczak E, Hemkens LG, et al. Protocol for the development of a CONSORT extension for RCTs using cohorts and routinely collected health data. *Res Integr Peer Rev*. 2018;3:9.

23. Moher D, Schulz KF, Simera I, Altman DG. Guidance for developers of health research reporting guidelines. *PLoS Med*. 2010;7(2):e1000217.

24. Equator Network. http://www.equator-network.org/library/reporting-guidelines-under-development/. Accessed November 2, 2020.

25. Relton, C, Burbach, M, Collett, C, at al. The ethics of 'Trials within Cohorts'(TwiCs): 2nd international symposium. *Trials*. 2017; 18(2):1-10.

26. von Elm E, Altman DG, Egger M, et al. The Strengthening the Reporting of Observational Studies in Epidemiology (STROBE) Statement: guidelines for reporting observational studies. *BMJ*. 2007;335(7624):806-808.

For peer review only - http://bmjopen.bmj.com/site/about/guidelines.xhtml

27. Kwakkenbos L, Imran M, Mc Cord KA, et al. Protocol for a scoping review to support development of a CONSORT extension for randomised controlled trials using cohorts and routinely collected health data. *BMJ Open*. 2018;8(8):e025266.

28. Trevelyan, EG, & Robinson, N. Delphi methodology in health research: how to do it?. *Eur J Integr Med.* 2015; 7(4): 423-428.

29. Okoli C, Pawlowski SD. The Delphi method as a research tool: an example, design considerations and applications. *Inform Manage*. 2004;42:15-29.

30. Hasson F, Keeney S, McKenna H. Research guidelines for the Delphi survey technique. *J Adv Nurs*. 2000;32:1008-15.

31. McCall SJ, Imran M, Hemkens LG, et al. A review of randomised controlled trials conducted using electronic health records with an assessment of completenessand transparency of reporting: study protocol. 2019. Available from: https://osf.io/p6wa4/. Accessed November 2, 2020.

32. Mc Cord KA., Imran M, McCall SJ, et al. Systematic analysis of the reporting transparency and completeness of randomized controlled trials using registries: a protocol. 2019. Available from: https://osf.io/x75gf/. Accessed November 2, 2020.

33. Imran M, Mc Cord KA, McCall SJ, et al. Protocol for a Completeness and Transparency of Reporting Study of Randomised Controlled Trials Conducted using Administrative Databases. 2019. Available from: https://osf.io/dp23x/. Accessed November 2, 2020.

34. Agha RA, Fowler AJ, Rajmohan S, Barai I, Orgill DP; PROCESS Group. Preferred reporting of case series in surgery; the PROCESS guidelines. *Int J Surg*. 2016;36(Pt A):319-323.

35. Vohra S, Shamseer L, Sampson M, et al. CONSORT extension for reporting N-of-1 trials (CENT) 2015 Statement. *BMJ*. 2015;350:h1738.

For peer review only - http://bmjopen.bmj.com/site/about/guidelines.xhtml

Table 1. Consensus meeting discussions and advisory decisions for the checklist items.

Section/Topic	CON-SORT 2010 Item	CON-SORT Ext. Item		CONSORT 2010 item	Suggested modified or additional extension items	Consensus Status (Delphi)	Summary of the discussion, decisions and suggestions made during the CONSORT-ROUTINE in-person consensus meeting	Final checklist item to be included in CONSORT-ROUTINE
**Title and abstract**								
	1a	1a		Identification as a randomised trial in the title	Identification as a randomised trial in the title, including that it was a trial conducted using a cohort or routinely collected source of data (Modified)	Not reached	Discussed the need for a modification to the original item. It was noted that multiple databases or types of databases could be used to conduct a trial and stating all would not be feasible as journals might have title length restrictions.<br><br>Decision: Do not include the modification and retain the CONSORT 2010 item; expand the E&E text for clarification	*Identification as a randomised trial in the title*
	1b	1b		Structured summary of trial design, methods, results, and conclusions (for specific guidance see CONSORT for abstracts)			No suggested modification.<br><br>Decision: Retain the CONSORT 2010 item.<br><br>Note: Additional Item 1c was later merged with this item (see below)	*Structured summary of trial design, methods, results, and conclusions (for specific guidance see CONSORT for abstracts). Specify that a cohort or routinely collected data were used to conduct the trial and, if applicable, provide the name of the cohort or routinely collected database(s)*
					The source(s) of data used to conduct the trial should be specified in the abstract (Additional)	Reached for inclusion	Noted the importance of stating the cohort or routinely collected database(s) used to conduct the trial in the abstract, if not in the title.<br><br>Decision: Include the suggested new item with revisions.<br><br>Note: The item was later merged with Item 1b from CONSORT 2010	
					If linkage between multiple sources of data was conducted for the study, this should be clearly stated in the abstract (Additional)	Not reached	Mixed views on the necessity of reporting the suggested new item in the abstract. Agreed that linkage is important to report in the body of the paper, but not necessarily the abstract.<br><br>Decision: Do not include the suggested new item.	
					The proportion of participants offered and the proportion that accepted the intervention should be reported (for trials conducted using the cohort multiple RCT design) (Additional)	Reached for inclusion	Mixed views on the necessity of reporting the suggested new item in the abstract. Agreement that the information is important to report but not essential for the abstract due to word count restrictions. In addition, this applies to one trial design used in cohorts, but not all cohort trials and not trials using other types of data. The item was merged with CONSORT 2010 item 13a (14a in the final extension checklist) pertaining to participant flow.<br><br>Decision: Do not include the suggested new item in the abstract but include in item 14a in the final extension checklist.	
**Introduction**								
Background and objectives	2a	2a		Scientific background and explanation of rationale			Discussed the importance of reporting the rationale for conducting the trial using a cohort or routinely collected database but decided against modifying original CONSORT 2010 item.<br><br>Decision: Retain the CONSORT 2010 item.	*Scientific background and explanation of rationale*
	2b	2b		Specific objectives or hypotheses			No suggested modification.<br><br>Decision: Retain the CONSORT 2010 item.	*Specific objectives or hypotheses*
**Methods**								
Trial design	3a	3a		Description of trial design (such as parallel, factorial) including allocation ratio	Description of trial design (such as parallel, factorial) including allocation ratio, the source(s) of data used to conduct the trial (such as cohort, registry) and how the data		Noted that key elements of the study design and cohort or database(s) used for the trial should be stated early in the methods section, as well as the extent to which the database was used in the trial.	*Description of trial design (such as parallel, factorial) including allocation ratio, that a cohort or routinely collected database(s) used to conduct the trial (such as electronic health record, registry) and how the data*

For peer review only - http://bmjopen.bmj.com/site/about/guidelines.xhtml

					are used within the trial (such as identification of eligible trial participants, trial outcomes) (Modified)		<u>Decision</u>: Include the modified item.	*were used within the trial (such as identification of eligible trial participants, trial outcomes)*
	3b	3b		Important changes to methods after trial commencement (such as eligibility criteria), with reasons			No suggested modification.<br><br><u>Decision</u>: Retain the CONSORT 2010 item.	*Important changes to methods after trial commencement (such as eligibility criteria), with reasons*
Cohort or routinely collected database (Additional header)		RCHD-1			Description of the source(s) of data used to conduct the trial, including the setting, locations, relevant dates, periods of recruitment, follow-up, and data collection (Additional)	Reached for inclusion	Agreed on the importance of reporting the item.<br><br><u>Decision</u>: Include the suggested new item.	*Name, if applicable, and description of the cohort or routinely collected database(s) used to conduct the trial, including information on the setting (such as primary care), locations, and dates, (such as periods of recruitment, follow-up, and data collection)*
					Describe indicators of the quality of the source(s) of data used to conduct the trial including what types of quality checks have been performed and the entity responsible for the data (Additional)	Reached for inclusion	Mixed views on the necessity of the suggested new item. There were concerns that "quality" is vague and the term "accuracy and completeness" may better clarify the intent of the item. It was acknowledged that the accuracy and completeness of the cohort or database is important to report while (i) selecting participants and (ii) ascertaining outcomes.<br><br><u>Decision</u>: Do not include the suggested new item as a stand-alone item. The item was merged with extension items 5a and 7b (pertaining to participant selection and outcome ascertainment) in the finalised checklist.	
					Describe modifications to the data collected in the source(s) of data used to conduct the trial, such as adding data items, if applicable (Additional)	Reached for inclusion	Agreed that the suggested item is not necessarily unique to trials conducted using cohorts and routinely collected data.<br><br><u>Decision</u>: Do not include the suggested new item; expand the E&E text for clarification.	
					Describe additional sources of data used to conduct the trial, if any (Additional)	Reached for inclusion	Mixed views on the necessity of the suggested new item as it is not unique to trials conducted using cohorts and routinely collected data.<br><br><u>Decision</u>: Do not include the suggested new item; expand the E&E text for clarification.	
		RCHD-2			Give the eligibility criteria, the sources and methods of selection of participants, and methods of follow-up (for trials conducted using cohorts or registries) (Additional)	Reached for inclusion	Discussed the importance of reporting the eligibility criteria for inclusion in the cohort or routinely collected database(s), but there was concern that elements related to follow-up are not specific to trials conducted using cohorts and routinely collected data.<br><br><u>Decision</u>: Include the suggested new item with revisions; expand the E&E text for clarification of other aspects.	*Eligibility criteria for participants in the cohort or routinely collected database(s)*
		RCHD-3			Detail any use of record linkage across sources of data, the methods of linkage and methods of quality evaluation, if applicable (Additional)	Reached for inclusion	Suggestion to integrate wording from RECORD checklist for clarity.<br><br><u>Decision</u>: Include the suggested new item adapted from RECORD.	*State whether the study included person-level, institutional-level, or other data linkage across two or more databases and, if so, linkage techniques and methods used to evaluate completeness and accuracy of linkage*
					Describe if (and how) participants were informed about the potential use of their data in randomised trials (Additional)	Not reached	Mixed views on the necessity of the item as some believed that ethics considerations are beyond the scope of CONSORT, and ethics does not appear in CONSORT 2010. The group agreed to include the item as consent is an important issue with unique aspects in these trials, but that this should be presented as part of trial participants section.<br><br><u>Decision</u>: Include the suggested new item with revisions and move to section "Trial participants" as Item 5c.	
Trial Participants (Modified header)	4a	4a		Eligibility criteria for participants	Eligibility criteria for trial participants (Modified)		Agreed to merge with suggested new item (see next row).<br><br><u>Decision</u>: Merge with suggested new item, "Provide details of how eligible clusters/participants were identified from the source(s) of data used to conduct the trial".	*Eligibility criteria for trial participants, including information on how to access the list of codes and algorithms used to identify eligible participants, information on accuracy and completeness of data used to ascertain eligibility, and methods used to validate accuracy and completeness (e.g., monitoring, adjudication), if applicable*
					Provide details of how eligible clusters/participants were identified from the	Reached for inclusion	Suggested merging with CONSORT 2010 Item 4a (5a in final checklist) and address accuracy and completeness of data.	

For peer review only

					source(s) of data used to conduct the trial (Additional)		Decision: Merge the suggested new item with CONOSRT 2010 item 4a (5a in the final checklist).	
	4b	4b		Settings and locations where the data were collected	Settings and locations where the trial data were collected (Modified)	Reached for inclusion	The word "trial" was dropped as the header "Trial participants" clarifies the intent of the item.<br><br>Decision: Retain the CONSORT 2010 item; expand the E&E text for clarification.	*Settings and locations where the data were collected*
		RCHD-4			Details of information provided to participants from the source(s) of data who are selected for recruitment or inclusion in the trial, including any differences in information provided across trial arms (Additional)	Not reached	Extended discussions on the importance of the item as it might only be applicable to cmRCTs. Agreement to formulate as a general item on consent as Item 5c.<br><br>Decision: Do not include the suggested new item. The consent item was simplified and moved to this section. Expand the E&E text for clarification of consent issues.	*Describe whether and how consent was obtained*
Interventions	5	5		The interventions for each group with sufficient details to allow replication, including how and when they were actually administered			No suggested modification.<br><br>Decision: Retain the CONSORT 2010 item; expand the E&E text for clarification.	*The interventions for each group with sufficient details to allow replication, including how and when they were actually administered*
					Describe how the source(s) of data was used to implement the intervention, if applicable (e.g., for trials conducted using electronic health records) (Additional)	Reached for inclusion	Debated the necessity of the new item as it is only applicable to trials conducted using electronic health records that may be used as intervention tools.<br><br>Decision: Do not include the suggested new item; expand the E&E text for clarification.	
Outcomes	6a	6a		Completely defined pre-specified primary and secondary outcome measures, including how and when they were assessed			Suggestion to merge with proposed new item, "Provide source(s) of data for each outcome" (see below).<br><br>Decision: Item merged with suggested new item and included in the final checklist.	*Completely defined pre-specified primary and secondary outcome measures, including how and when they were ascertained and the cohort or routinely collected database(s) used to ascertain each outcome*
					Provide source(s) of data for each outcome (Additional)	Reached for inclusion	Suggestion to merge with CONSORT 2010 item 6a.<br><br>Decision: Item merged with CONSORT 2010 item 6a (7a in the final checklist).	
		RCHD-5			Provide a list of codes and algorithms used to define (and/or derive) the outcomes as supplementary information, including validation, if applicable (Additional)	Not reached	Acknowledged the importance of reporting the list of codes and algorithms for ascertaining outcomes along with the accuracy and completeness of data and validation.<br><br>Decision: Include the suggested new item with revisions.	*Information on how to access the list of codes and algorithms used to define or derive the outcomes from the cohort or routinely collected database(s) used to conduct the trial, information on accuracy and completeness of outcome variables, and methods used to validate accuracy and completeness (e.g., monitoring, adjudication), if applicable*
					Detail any adjudication or external validation of data items from the source(s) of data used to conduct the trial, if applicable (Additional)	Reached for inclusion	Acknowledged the importance of reporting the item. There was agreement that validation should be reported while selecting participants and ascertaining outcomes and included as part of items 5a and 7b of extension checklist.<br><br>Decision: Address elements of proposed item as part of items 5a and 7b in the final checklist.	
	6b	6b		Any changes to trial outcomes after the trial commenced, with reasons			No suggested modification.<br><br>Decision: Retain the CONSORT 2010 item.	*Any changes to trial outcomes after the trial commenced, with reasons*
Sample size	7a	7a		How sample size was determined			No suggested modification.<br><br>Decision: Retain the CONSORT 2010 item.	*How sample size was determined*
	7b	7b		When applicable, explanation of any interim analyses and stopping guidelines			No suggested modification.<br><br>Decision: Retain the CONSORT 2010 item.	*When applicable, explanation of any interim analyses and stopping guidelines*
Randomisation:								
Sequence generation	8a	8a		Method used to generate the random allocation sequence			No suggested modification.<br><br>Decision: Retain the CONSORT 2010 item; expand the E&E text for clarification.	*Method used to generate the random allocation sequence*
	8b	8b		Type of randomisation; details of any restriction (such as blocking and block size)			No suggested modification.<br><br>Decision: Retain the CONSORT 2010 item.	*Type of randomisation; details of any restriction (such as blocking and block size)*

For peer review only

Allocation concealment mechanism	9	9		Mechanism used to implement the random allocation sequence (such as sequentially numbered containers), describing any steps taken to conceal the sequence until interventions were assigned	Mechanism used to implement the random allocation sequence, describing any steps taken to conceal the sequence until interventions were assigned, such as using automated random sequence generation concealed within source(s) of data (Modified)	Reached for inclusion	Discussion to clarify wording of the item.<br><br>Decision: Include the modified item with revisions.	*Mechanism used to implement the random allocation sequence (such as embedding an automated randomiser within the cohort or routinely collected database(s)), describing any steps taken to conceal the sequence until interventions were assigned*
Implementation	10	10		Who generated the random allocation sequence, who enrolled participants, and who assigned participants to interventions			No suggested modification.<br><br>Decision: Retain the CONSORT 2010 item.	*Who generated the random allocation sequence, who enrolled participants, and who assigned participants to interventions*
Blinding	11a	11a		If done, who was blinded after assignment to interventions (for example, participants, care providers, those assessing outcomes) and how			No suggested modification.<br><br>Decision: Retain the CONSORT 2010 item.	*If done, who was blinded after assignment to interventions (for example, participants, care providers, those assessing outcomes) and how*
	11b	11b		If relevant, description of the similarity of interventions			No suggested modification.<br><br>Decision: Retain the CONSORT 2010 item.	*If relevant, description of the similarity of interventions*
Statistical methods	12a	12a		Statistical methods used to compare groups for primary and secondary outcomes			No suggested modification.<br><br>Decision: Retain the CONSORT 2010 item.	*Statistical methods used to compare groups for primary and secondary outcomes*
	12b	12b		Methods for additional analyses, such as subgroup analyses and adjusted analyses			No suggested modification.<br><br>Decision: Retain the CONSORT 2010 item.	*Methods for additional analyses, such as subgroup analyses and adjusted analyses*
**Results**								
Participant flow (a diagram is strongly recommended)	13a	13a		For each group, the numbers of participants who were randomly assigned, received intended treatment, and were analysed for the primary outcome	Describe in detail the numbers of clusters/participants in the source(s) of data used to conduct the trial, number screened for eligibility, randomly assigned, offered and accepted interventions (e.g., cohort multiple RCTs), received intended treatment, and analysed for the primary outcome (Modified)	Reached for inclusion	Suggestion to form a committee to draft example flow diagram and oversee the E&E.<br><br>Decision: Include the modified item; committee to oversee the E&E development.	*For each group, the number of participants in the cohort or routinely collected database(s) used to conduct the trial and the numbers screened for eligibility, randomly assigned, offered and accepted interventions (e.g., cohort multiple RCTs), received intended treatment, and analysed for the primary outcome*
					Describe any linkage of multiple sources of data, including the number of clusters/participants successfully linked (Additional)	Reached for inclusion	Debated the necessity of the item as a stand-alone item as linkage was addressed in item 4c. Suggested to include the number of clusters/participants successfully linked as part of the flow diagram.<br><br>Decision: Do not include the suggested new item; expand the E&E text for clarification.	
	13b	13b		For each group, losses and exclusions after randomisation, together with reasons			No suggested modification. Discussed that the item should be tied to data accuracy and completeness, and linkage.<br><br>Decision: Retain the CONSORT 2010 item; expand the E&E text for clarification.	*For each group, losses and exclusions after randomisation, together with reasons*
Recruitment	14a	14a		Dates defining the periods of recruitment and follow-up			No suggested modification.<br><br>Decision: Retain the CONSORT 2010 item.	*Dates defining the periods of recruitment and follow-up*
	14b	14b		Why the trial ended or was stopped			No suggested modification.<br><br>Decision: Retain the CONSORT 2010 item.	*Why the trial ended or was stopped*
Baseline data	15	15		A table showing baseline demographic and clinical characteristics for each group			No suggested modification.<br><br>Decision: Retain the CONSORT 2010 item.	*A table showing baseline demographic and clinical characteristics for each group*
					A table showing baseline demographic and clinical characteristics for eligible participants who participated in the trial and those who did not (Additional)	Reached for inclusion	Agreement to not include the suggested new item as a stand-alone item. The information should be reported if possible, but not necessary, and implications should be addressed as part of "Generalisability" (Item 21).<br><br>Decision: Do not include the suggested new item.	
Numbers analysed	16	16		For each group, number of participants (denominator) included in each analysis and whether the analysis was by original assigned groups			No suggested modification.<br><br>Decision: Retain the CONSORT 2010 item.	*For each group, number of participants (denominator) included in each analysis and whether the analysis was by original assigned groups*
Outcomes and estimation	17a	17a		For each primary and secondary outcome, results for each group, and the estimated effect size and its precision (such as 95% confidence interval)			No suggested modification.<br><br>Decision: Retain the CONSORT 2010 item.	*For each primary and secondary outcome, results for each group, and the estimated effect size and its precision (such as 95% confidence interval)*
	17b	17b		For binary outcomes, presentation of both absolute and relative effect sizes is recommended			No suggested modification.<br><br>Decision: Retain the CONSORT 2010 item.	*For binary outcomes, presentation of both absolute and relative effect sizes is recommended*

For peer review only

Ancillary analyses	18	18		Results of any other analyses performed, including subgroup analyses and adjusted analyses, distinguishing pre-specified from exploratory			No suggested modification.<br><br>Decision: Retain the CONSORT 2010 item.	*Results of any other analyses performed, including subgroup analyses and adjusted analyses, distinguishing pre-specified from exploratory*
					If outcomes for eligible patients in the existing source(s) of data who were not included in the trial are known, they should be reported (Additional)	Not reached	Agreement to not include the suggested new item as a stand-alone item. The information should be reported if possible, but not necessary, and implications should be addressed as part of "Generalisability".<br><br>Decision: Do not include the suggested new item; expand the E&E text for clarification in the "Generalisability" section.	
Harms	19	19		All important harms or unintended effects in each group (for specific guidance see CONSORT for harms)			No suggested modification.<br><br>Decision: Retain the CONSORT 2010 item.	*All important harms or unintended effects in each group (for specific guidance see CONSORT for harms)*
**Discussion**								
Limitations	20	20		Trial limitations, addressing sources of potential bias, imprecision, and, if relevant, multiplicity of analyses			No suggested modification.<br><br>Decision: Retain the CONSORT 2010 item.	*Trial limitations, addressing sources of potential bias, imprecision, and, if relevant, multiplicity of analyses*
					Discuss the implications of using data that were not created or collected to answer the specific research question(s) (Additional)	Reached for inclusion	Discussed that using routinely collected data is not necessarily a limitation, and the content of this item should be addressed in the "Interpretation" section.<br><br>Decision: Do not include the suggested new item; merge with CONSORT 2010 item 22 (23 in the final checklist); expand the E&E text for clarification in the "Generalisability" section.	
Generalisability	21	21		Generalisability (external validity, applicability) of the trial findings			No suggested modification. Agreement to elaborate on the representativeness of the cohort or routinely collected database(s) used for the trial, including issues related to characteristics of eligible cohort or database participants who do not agree to participate in trial.<br><br>Decision: Retain the CONSORT 2010 item; expand the E&E text for clarification.	*Generalisability (external validity, applicability) of the trial findings*
Interpretation	22	22		Interpretation consistent with results, balancing benefits and harms, and considering other relevant evidence			Item merged with the proposed new item "Discuss the implications of using data that were not created or collected to answer the specific research question(s)".<br><br>Decision: Include the modified item.	*Interpretation consistent with results, balancing benefits and harms, and considering other relevant evidence, including the implications of using data that were not collected to answer the trial research questions*
**Other information**								
Registration	23	23		Registration number and name of trial registry			No suggested modification.<br><br>Decision: Retain the CONSORT 2010 item.	*Registration number and name of trial registry*
Protocol	24	24		Where the full trial protocol can be accessed, if available			No suggested modification.<br><br>Decision: Retain the CONSORT 2010 item; expand the E&E text for clarification.	*Where the full trial protocol can be accessed, if available*
Funding	25	25		Sources of funding and other support (such as supply of drugs), role of funders	Sources of funding and other support for the trial and the existing source(s) of data, role of funders (Modified)	Reached for inclusion	Suggested minor revision to the item.<br><br>Decision: Include the modified item with revision.	*Sources of funding and other support for both the trial and the cohort or routinely collected database(s), role of funders*

For peer review only

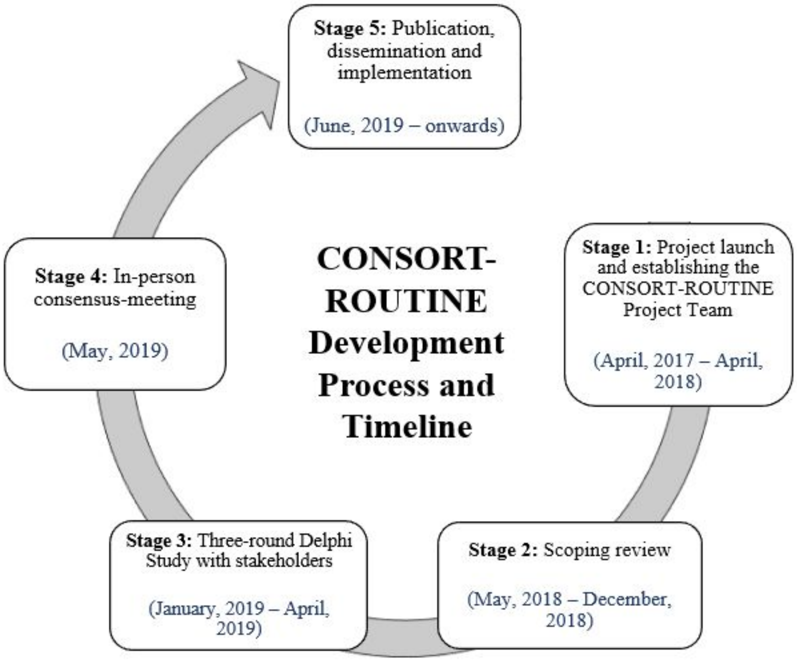

For peer review only - http://bmjopen.bmj.com/site/about/guidelines.xhtml

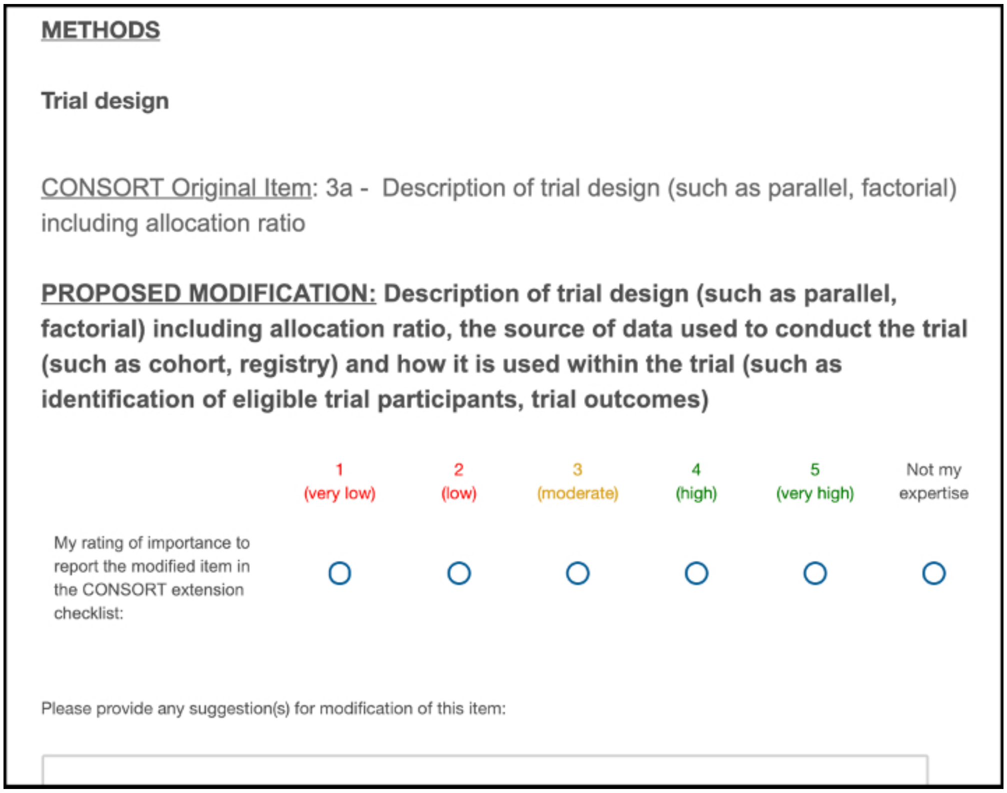

METHODS

Trial design

CONSORT Original Item: 3a - Description of trial design (such as parallel, factorial) including allocation ratio

PROPOSED MODIFICATION: Description of trial design (such as parallel, factorial) including allocation ratio, the source of data used to conduct the trial (such as cohort, registry) and how it is used within the trial (such as identification of eligible trial participants, trial outcomes)

	1 (very low)	2 (low)	3 (moderate)	4 (high)	5 (very high)	Not my expertise
My rating of importance to report the modified item in the CONSORT extension checklist:	○	○	○	○	○	○

Please provide any suggestion(s) for modification of this item:

Figure 2

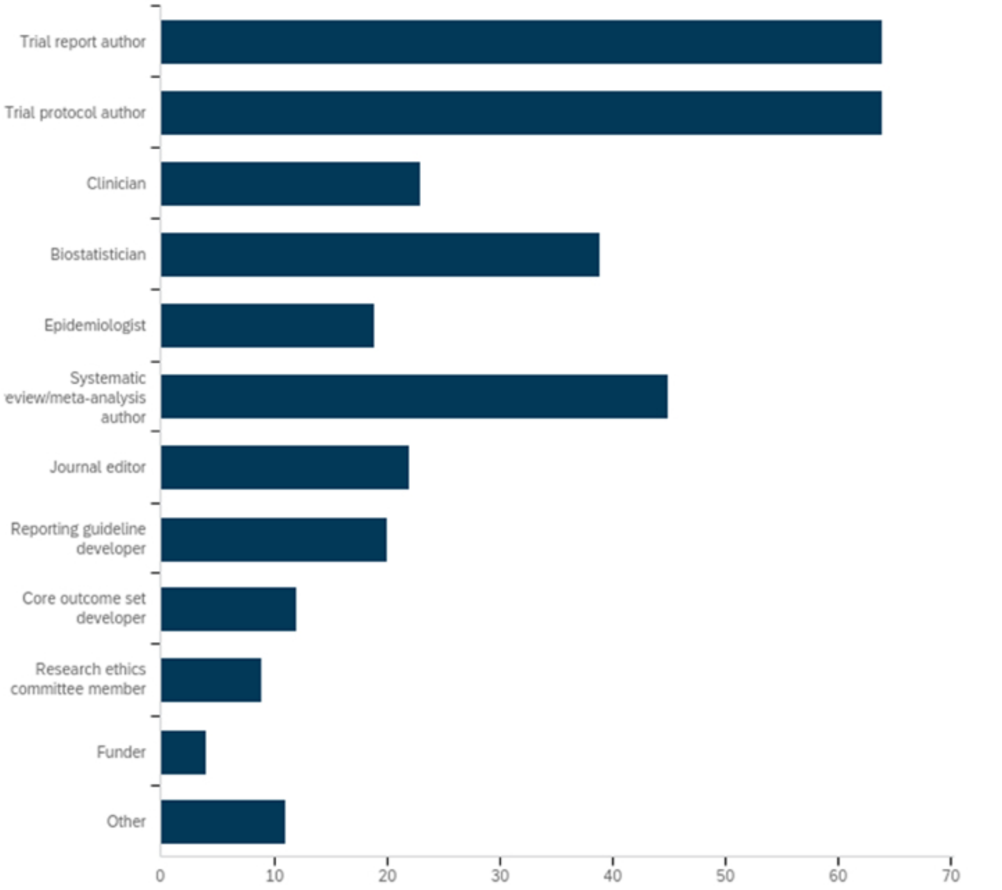
Trial report author
Trial protocol author
Clinician
Biostatistician
Epidemiologist
Systematic
review/meta-analysis
author
Journal editor
Reporting guideline
developer
Core outcome set
developer
Research ethics
committee member
Funder
Other
0
10
20
30
40
50
60
70

Figure 3

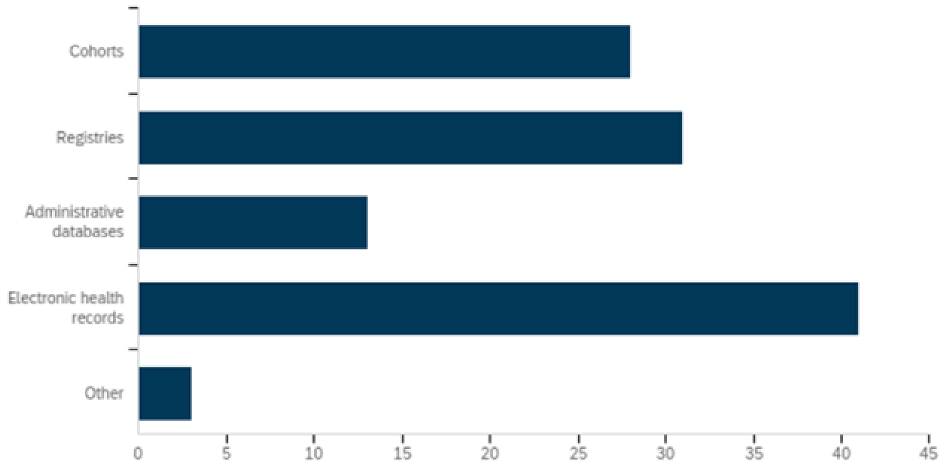
Cohorts
Registries
Administrative databases
Electronic health records
Other
0
5
10
15
20
25
30
35
40
45

Figure 4

Supplementary File 1 – Electronic Search Strategies

Searches were run in both MEDLINE and Cochrane Methodology Register simultaneously. As an example, in the registries search, lines 1-11 are the MEDLINE search and lines 12-15 are tailored for the Cochrane Methodology Register. The final lines of each search isolate the records from each database, combine them so duplicate records can be removed, then isolate the remaining records so they can be downloaded and imported into Reference Manager using customized import filters.

Searches for RCTs embedded in Registries

1. ((registry or registries) adj5 randomi#ed).ab,kf,ti.
2. ((registry or registries) adj5 RCT*).ab,kf,ti.)
3. ((registry or registries) adj5 controlled trial*).ab,kf,ti.
4. ((registry or registries) adj5 (RRCT* or R RCT*)).ab,kf,ti.
5. or/1-4
6. (meta analy* or metaanaly* or metanaly* or systematic review*).af.
7. 5 not 6
8. Registries/
9. limit 8 to randomized controlled trial
10. 7 or 9
11. limit 10 to yr="2007 - 2018"
12. (registry or registries).ab,kf,ti.
13. (random* or RCT).ti,ab,kw.
14. 12 and 13
15. limit 14 to yr="2007 - 2018"
16. 11 use medall
17. 15 use clcmr
18. 16 or 17 (1240)
19. remove duplicates from 18
20. 19 use medall
21. 19 use clcmr

Searches for RCTs embedded in Cohorts

1. (cohort adj5 (randomi#ed adj5 trial*)).ab,kf,ti.
2. (cohort adj5 RCT*).ab,kf,ti.
3. (cohort adj5 controlled trial*).ab,kf,ti.
4. (cmRCT or Cohort Multiple Randomised Controlled Trial*).ab,kf,ti.
5. or/1-4
6. cohort.af.
7. (embed* adj8 randomi#ed).ab,kf,ti.
8. (embed* adj8 RCT*).ab,kf,ti.
9. (embed* adj8 controlled trial*).ab,kf,ti.
10. or/7-9
11. 6 and 10
12. (pragmatic adj5 RCT*).ab,kf,ti.
13. (pragmatic adj5 randomi#ed).ab,kf,ti.

For peer review only

14. (pragmatic adj5 controlled trial*).ab,kf,ti.
15. or/12-14
16. 6 and 15
17. 5 or 11 or 16
18. (meta analy* or metaanaly* or metanaly* or systematic review*).af.
19. 17 not 18
20. limit 19 to yr="2007 - 2018"
21. ((Cohort* and (random* or RCT)) or cmRCT).ti,ab,kw.
22. limit 21 to yr="2007 - 2018"
23. 20 use medall
24. 22 use clcmr
25. 23 or 24
26. remove duplicates from 25
27. 26 use medall
28. 26 use clcmr

Searches for RCTs embedded in Electronic Health Records

1. randomized controlled trial.pt.
2. controlled clinical trial.pt.
3. randomi?ed.ab.
4. placebo.ab.
5. randomly.ab.
6. clinical trials as topic.sh.
7. trial.ti.
8. or/1-7
9. exp animals/ not humans.sh.
10. 8 not 9
11. exp Electronic Health Records/
12. (EHR or electronic health record*).ab,kf,ti.
13. (EMR or electronic medical record*).ab,kf,ti.
14. (PHR or personal health record*).ab,kf,ti.
15. (EPR or electronic patient record*).ab,kf,ti.
16. exp Health Records, Personal/
17. or/11-16
18. 10 and 17
19. limit 18 to yr="2007 - 2018"
20. (Electronic health record or electronic health records or EHR).ti,ab,kw.
21. (Electronic medical record or electronic medical records or EMR).ti,ab,kw.
22. (Electronic patient record or electronic patient records or EPR).ti,ab,kw.
23. or/20-22
24. limit 23 to yr="2007 - 2018"
25. 19 use medall
26. 24 use clcmr
27. 25 or 26
28. remove duplicates from 27
29. 28 use medall
30. 28 use clcmr

For peer review only

Searches for RCTs embedded in Administrative Databases

1. randomized controlled trial.pt.
2. controlled clinical trial.pt.
3. randomi?ed.ab.
4. placebo.ab.
5. randomly.ab.
6. clinical trials as topic.sh.
7. trial.ti.
8. or/1-7
9. exp animals/ not humans.sh.
10. 8 not 9
11. administrative data*.ab,kf,ti.
12. healthcare data*.ab,kf,ti.
13. health care data*.ab,kf,ti.
14. or/11-13
15. 10 and 14
16. (administrative adj5 data*).ti,ab,kw.
17. health care data*.ti,ab,kw.
18. healthcare data*.ti,ab,kw.
19. or/16-18
20. (random* or RCT).ti,ab,kw.
21. 19 and 20
22. limit 15 to yr="2007 - 2018"
23. 22 use medall
24. limit 21 to yr="2007 - 2018"
25. 22 use clcmr

For peer review only

CONSORT Extension for the Reporting of Randomised Controlled Trials Conducted Using Cohorts and Routinely Collected Health Data: Checklist with Explanation and Elaboration

Linda Kwakkenbos[1]; Mahrukh Imran[2]; Stephen J. McCall[3,4]; Kimberly A. Mc Cord[5]; Ole Fröbert[6]; Lars G. Hemkens[5,7,8,]; Merrick Zwarenstein[9,10]; Clare Relton[11]; Danielle B. Rice[2,12]; Sinéad M. Langan[13]; Eric I. Benchimol[10,14,15]; Lehana Thabane[16]; Marion K. Campbell[17]; Margaret Sampson[18]; David Erlinge[19]; Helena M. Verkooijen[20,21]; David Moher[22]; Isabelle Boutron[23,24]; Philippe Ravaud[23,24]; Jon Nicholl[25]; Rudolf Uher[26]; Maureen Sauvé[27,28]; John Fletcher[29]; David Torgerson[30]; Chris Gale[31]; Edmund Juszczak[3,32]; Brett D. Thombs[2,33]

[1]Behavioural Science Institute, Clinical Psychology, Radboud University, Nijmegen, the Netherlands; [2]Lady Davis Institute for Medical Research, Jewish General Hospital, Montreal, Canada; [3]National Perinatal Epidemiology Unit Clinical Trials Unit, Nuffield Department of Population Health, University of Oxford, Oxford, UK; [4]Center for Research on Population and Health, Faculty of Health Sciences, American University of Beirut, Ras Beirut, Lebanon; [5]Basel Institute for Clinical Epidemiology and Biostatistics, Department of Clinical Research, University Hospital Basel, University of Basel, Basel, Switzerland; [6]Örebro University, Faculty of Health, Department of Cardiology, Örebro, Sweden; [7]Meta-Research Innovation Center at Stanford (METRICS), Stanford University, Palo Alto, USA; [8]Meta-Research Innovation Center Berlin (METRIC-B), Berlin Institute of Health, Berlin, Germany; [9]Department of Family Medicine, Western University, London, Canada; [10]ICES, Toronto, Canada; [11]Centre for Clinical Trials and Methodology, Barts Institute of Population Health Science, Queen Mary University,

For peer review only - http://bmjopen.bmj.com/site/about/guidelines.xhtml

London, UK; [12]Department of Psychology, McGill University, Montréal, Québec, Canada; [13]Faculty of Epidemiology and Population Health, London School of Hygiene and Tropical Medicine, London, UK; [14]Department of Paediatrics, University of Toronto, Toronto, Canada; [15]Division of Gastroenterology, Hepatology and Nutrition and Child Health Evaluative Sciences, SickKids Research Institute, The Hospital for Sick Children, Toronto, Canada; [16]Department of Health Research Methods, Evidence, and Impact, McMaster University, Hamilton, Canada; [17]Health Services Research Unit, University of Aberdeen, Aberdeen, UK; [18]Library Services, Children's Hospital of Eastern Ontario, Ottawa, Canada; [19]Department of Cardiology, Clinical Sciences, Lund University, Lund, Sweden; [20]University Medical Center Utrecht, Utrecht, the Netherlands; [21]University of Utrecht, Utrecht, the Netherlands; [22]Centre for Journalology, Clinical Epidemiology Program, Ottawa Hospital Research Institute, Ottawa, Canada; [23]Université de Paris, Centre of Research Epidemiology and Statistics (CRESS), Inserm, INRA, F-75004, Paris, France; [24]Centre d'Épidémiologie Clinique, Assistance Publique–Hôpitaux de Paris (AP-HP), Hôpital Hôtel Dieu, F-75004, Paris, France; [25]School of Health and Related Research, University of Sheffield, Sheffield, UK; [26]Department of Psychiatry, Dalhousie University, Halifax, Canada; [27]Scleroderma Society of Ontario, Hamilton, Canada; [28]Scleroderma Canada, Hamilton, Canada; [29]British Medical Journal; [30]York Trials Unit, Department of Health Sciences, University of York, York, UK; [31]Neonatal Medicine, School of Public Health, Faculty of Medicine, Imperial College London, Chelsea and Westminster campus, London, UK; [32]Nottingham Clinical Trials Unit, University of Nottingham, University Park, Nottingham, United Kingdom; [33]Departments of Psychiatry; Epidemiology, Biostatistics and Occupational Health; Medicine; and Educational and Counselling Psychology; and Biomedical Ethics Unit, McGill University, Montreal, Canada.

For peer review only

For peer review only - http://bmjopen.bmj.com/site/about/guidelines.xhtml

Address for Correspondence: Brett D. Thombs, PhD; Jewish General Hospital; 4333 Côte-Sainte-Catherine Road; Montreal, Quebec, Canada; H3T 1E4; Telephone: (514) 340-8222 ext. 25112; Email: brett.thombs@mcgill.ca.

For peer review only

ABSTRACT

Randomised controlled trials (RCTs) are increasingly conducted using cohorts or routinely collected health data, including registries, electronic health records, and administrative databases, to assess participant eligibility and facilitate recruitment, deliver an embedded intervention, collect trial outcome data, or a combination of these purposes. This report presents the Consolidated Standards of Reporting Trials (CONSORT) Extension for RCTs Conducted Using Cohorts and Routinely Collected Health Data. The extension was developed to address unique characteristics of trials conducted using these types of data with the goal of improving long-term reporting quality by setting standards early in the process of uptake of these trial designs. The extension was developed using a sequential approach, including a Delphi survey, a consensus meeting, and piloting the checklist. The checklist was informed by the CONSORT 2010 statement and two reporting guidelines for observational studies, the Strengthening the Reporting of Observational Studies in Epidemiology (STROBE) statement and the REporting of studies Conducted using Observational Routinely collected Data (RECORD) statement. The extension includes 8 items modified from the CONSORT 2010 statement and 5 new items. Reporting items with explanations and examples are provided, including key aspects of trials conducted using cohorts or routinely collected health data that require specific reporting considerations.

For peer review only - http://bmjopen.bmj.com/site/about/guidelines.xhtml

INTRODUCTION

Well-designed and conducted randomised controlled trials (RCTs) are the 'gold standard' of health care intervention research.[1-3] Important limitations often exist, however, including challenges in recruiting large and representative samples, prohibitive costs, and potentially limited real-world generalizability.[4-12] In an attempt to address such challenges, RCTs are increasingly being conducted using cohorts [4] and routinely collected health data, defined as health-related data collected without specific *a priori* research questions [13,14] and including registries,[15] electronic health records (EHRs),[16] and administrative databases.[17]

The Consolidated Standards of Reporting Trials (CONSORT) 2010 statement [18,19] provides guidance for reporting individually randomised parallel-groups trials. RCTs using cohorts and routinely collected health data share elements with trials covered in the CONSORT 2010 statement, but there are also unique reporting considerations.[20] See Box 1. Because of the substantial overlap in the design, conduct, reporting and interpretation of trials conducted using cohorts and different types of routinely collected health data, we developed a single extension.

Development and Scope of the CONSORT Extension

The project was registered with the Enhancing the QUAlity and Transparency Of health Research (EQUATOR) Network,[21] and a protocol was published.[20] The extension was developed following a consensus-driven process [22] and included (1) substantiation of the need for a reporting guideline, (2) a scoping review to assess reporting quality and identify reporting considerations to include in a preliminary checklist version,[23] (3) a 3-round Delphi process to gather input on checklist items from stakeholders, (4) a consensus meeting to advise on items to

For peer review only - http://bmjopen.bmj.com/site/about/guidelines.xhtml

include and the checklist structure, and (5) publication, dissemination and implementation of the final checklist. Details on methods and results from each stage of the process are described elsewhere.[24] In brief, 27 items for consideration were initially developed by members of the CONSORT Extension Project Team based on review of items included in the CONSORT 2010 [18,19], Strengthening the Reporting of Observational Studies in Epidemiology (STROBE) [25], and REporting of studies Conducted using Observational Routinely-collected Data (RECORD) [26] statements, as well as discussions with steering committee members. All items were rated in Delphi Round 1. In Round 2, 13 items were rated, and 11 items were rated in Round 3. Response rates for the Delphi Study were 92 of 125 (74%) invited participants in Round 1, 77 of 92 (84%) Round 1 completers in Round 2, and 62 of 77 (81%) Round 2 completers in Round 3. Members of the Project Team attended an in-person consensus meeting, where Delphi results were considered, and a preliminary checklist was developed. The preliminary version of the checklist was pilot tested by 17 people with experience in trials conducted using cohorts and routinely collected health data. In all stages of development, key stakeholders in trials research and potential end-users of the CONSORT extension were involved, including participants from a wide range of scientific disciplines and with diverse experience in conducting trials using cohorts and different types of routinely collected health databases.

Consistent with other CONSORT statements, this extension describes a minimum set of information that should be reported and provides a checklist to facilitate compliance. The extension applies to RCTs conducted using one or more cohorts or routinely collected health databases to (1) identify, recruit, or consent eligible participants; (2) implement an intervention; (3) collect trial data including outcomes; or a combination of these purposes. For RCTs that use

For peer review only - http://bmjopen.bmj.com/site/about/guidelines.xhtml

cohorts or routinely collected health data for outcome assessment only, some extension items may not be relevant.

The extension includes 8 items from the CONSORT 2010 statement that were modified and 5 new items. No items were removed from the CONSORT 2010 checklist. Table 1 shows the extension items compared to the CONSORT 2010 checklist. Table 2 is the integrated extension checklist.

For each modified and new item, this document describes the item, identifies whether the item was modified or new, provides examples of good reporting, explains the rationale for including the item, and elaborates on reporting considerations. For items that were unmodified from the CONSORT 2010 statement, but for which there are reporting considerations for trials conducted using cohorts or routinely collected health data, we have also provided an example and explanation. Examples of good reporting were retrieved from primary and secondary trial reports and, in some cases, trial protocols. For all items, explanations provided supplement those in the CONSORT 2010 explanation and elaboration.[18,19]

EXPLANATION AND ELABORATION

Title and abstract

Item 1a (unmodified): Identification as a randomised trial in the title.

Examples:

1) "Bivalirudin versus heparin in non-ST and ST-segment elevation myocardial infarction-a registry-based randomized clinical trial in the SWEDEHEART registry (the VALIDATE-SWEDEHEART trial)."[27]

For peer review only

2) "Clinical effectiveness and cost-effectiveness of a multifaceted podiatry intervention for falls prevention in older people: a multicentre cohort randomised controlled trial (the REducing Falls with ORthoses and a Multifaceted podiatry intervention trial)."[28]

Explanation:

Item 1a is meant to aid in the indexing and identification of RCT reports in electronic databases. The title, at minimum, should contain recognisable terminology identifying the study as a randomised trial. If word count permits, the type of trial (e.g. cohort multiple RCT, registry-based RCT) or the cohort or routinely collected health database(s) used to conduct the trial (e.g. SWEDEHEART Registry) should be provided.

Item 1b (modified):

CONSORT 2010 item: Structured summary of trial design, methods, results, and conclusions (for specific guidance see CONSORT for abstracts).

Modified CONSORT extension item: Structured summary of trial design, methods, results, and conclusions (for specific guidance see CONSORT for abstracts). Specify that a cohort or routinely collected health data were used to conduct the trial and, if applicable, provide the name of the cohort or routinely collected health database(s).

Examples:

1) "The TIMING study is a national, investigator-led, registry-based, multicentre, open-label, randomised controlled study. The Swedish Stroke Register is used for enrolment, randomisation and follow-up…."[29]

2) "The Department of Veterans Affairs (VA) MI-Plus study was a cluster-randomized trial involving 168 community-based primary care clinics and 847 providers in 26 states, the

For peer review only

Virgin Islands, and Puerto Rico….with the clinic as the randomization unit. We collected administrative data for 15,847 post-MI [myocardial infarction] patients and medical record data for 10,452 of these."[30]

Explanation:

Abstracts are used for electronic database indexing and are the most commonly read section of articles.[19,31] They provide information on the trial methodology and main results and allow readers to evaluate if the study likely addresses their information needs. In addition to CONSORT 2010 abstract elements, abstracts of trials using cohorts or routinely collected health databases should clearly describe the type of cohort or routinely collected health database used (e.g., registry-based trial), as per the examples above. The name of the cohort or database(s) used should also be reported, if applicable. Some databases, such as EHRs, are typically unnamed, in which case stating that an EHR was used is enough. Ideally, the abstract will clarify the purpose for which the cohort or routinely collected health database was used (e.g., identification of eligible participants, outcome assessment). There may also be additional information related to using cohorts or routinely collected health data that should be reported, depending on the specific trial design. Whenever possible, authors should report their abstract in a structured format.[18,19]

Introduction

Background and objectives

Item 2a (unmodified): Scientific background and explanation of rationale.

See CONSORT 2010.[18,19]

For peer review only - http://bmjopen.bmj.com/site/about/guidelines.xhtml

<u>*Item 2b (unmodified): Specific objectives or hypotheses.*</u>

See CONSORT 2010.[18,19]

Methods

Trial Design

<u>*Item 3a (modified):*</u>

<u>*CONSORT 2010 item*</u>: *Description of trial design (such as parallel, factorial) including allocation ratio.*

<u>*Modified CONSORT extension item*</u>: *Description of trial design (such as parallel, factorial) including allocation ratio, that a cohort or routinely collected health database(s) was used to conduct the trial (such as electronic health record, registry) and how the data were used within the trial (such as identification of eligible trial participants, trial outcomes).*

Examples:

1) "The Determination of the Role of Oxygen in Suspected Acute Myocardial Infarction (DETO2X-AMI) trial was a multicenter, parallel-group, open-label, registry-based, randomized, controlled trial in which routine supplemental oxygen therapy was compared with ambient air in the treatment of patients with suspected myocardial infarction who did not have hypoxemia at baseline. The trial used the national comprehensive Swedish Web System for Enhancement and Development of Evidence-Based Care in Heart Disease Evaluated According to Recommended Therapies (SWEDEHEART)….for patient enrollment and data collection…."[32]
2) "PATIENT was a parallel arm, pragmatic clinical trial in which 21,752 adults were randomized to receive either UC [usual care] or 1 of 2 interventions designed to increase

For peer review only - http://bmjopen.bmj.com/site/about/guidelines.xhtml

adherence to statins, angiotensin-converting enzyme inhibitors (ACEIs), and angiotensin receptor blockers (ARBs)….Using each region's EMR [electronic medical record], we identified participants 40 years and older with diabetes mellitus and/or cardiovascular disease (CVD), suboptimally (<90%) adherent to a statin or ACEI/ARB during the previous 12 months, and due or overdue for a refill.…Within each region, we randomly assigned a sample of eligible members to the 3 primary study arms (usual care and 2 intervention arms) in a 1:1:1 ratio at the study outset.…We used the EMR to capture age, race, gender, healthcare utilization for diabetes and CVD, and BP [blood pressure] and lipid levels."[33]

Explanation:

Per CONSORT 2010, the authors should describe the trial design (e.g. parallel group, cluster randomised), conceptual framework (e.g., superiority, equivalence or noninferiority), and allocation ratio (e.g., 1:1, 2:1). Additionally, they should describe that one or more cohorts or routinely collected health databases were used, how they were used (e.g., identification of eligible participants, intervention delivery, data collection including outcome assessment), and whether their use influenced other methodological choices that might have implications for generalizability and interpretability of trial results. Examples include constraints on trial eligibility criteria; timing between eligibility assessment, intervention delivery, and outcome assessment; and outcomes available.

<u>*Item 3b (unmodified):*</u> *Important changes to methods after trial commencement (such as eligibility criteria), with reasons.*

See CONSORT 2010.[18,19]

For peer review only - http://bmjopen.bmj.com/site/about/guidelines.xhtml

Cohort or routinely collected health database (new section subheading)

Item RCHD-1 (new). Name, if applicable, and description of the cohort or routinely collected health database(s) used to conduct the trial, including information on the setting (such as primary care), locations, and dates (such as periods of recruitment, follow-up, and data collection).

Examples:

1) "Family practices in England, Scotland, or Wales were eligible for the study if they were contributing data to the Clinical Practice Research Datalink (CPRD). The CPRD is a large database that includes the EHRs [electronic health records] of ≈ 7% of all UK general practices from 1987 to the present."[34]
2) "The [Scleroderma Patient-centered Intervention Network] SPIN Cohort is a convenience sample. Eligible SPIN Cohort patients are recruited at SPIN sites (www.spinsclero.com/en/sites) during regular medical visits, and written informed consent is obtained. A medical data form is submitted online by the site to enrol participants. Cohort participants complete outcome measures via the internet upon enrolment and subsequently every 3 months. SPIN Cohort enrollment started in March 2014 and is ongoing."[35]

Explanation:

This additional section covers a wider description of the cohort or routinely collected health database, which is different from the description of how the cohort or database was used in the trial, which is covered in section 4 (*Trial Participants*). Providing the name of the cohort or routinely collected health database allows readers to identify other studies, including trials,

For peer review only - http://bmjopen.bmj.com/site/about/guidelines.xhtml

conducted using the same cohort or database and consider the generalizability of the results to their setting. A description of the cohort or routinely collected health database, including geographical locations and clinical settings, enables readers to assess characteristics relevant to understanding the sampling frame for participant recruitment to the trial and the potential validity of the data for the research question. The authors should provide references to any publications that have described the cohort or database methods or characteristics of included participants.

Characteristics that could influence data quality and should be reported, if applicable, include the reason for data collection (e.g., clinical care, administrative purposes), the time period and related procedures by which data are collected, amongst others. Information on surgical procedures, for example, may be complete and accurate for administrative data derived from physician billing, since reimbursement depends on its accuracy. Associated diagnostic codes, however, might be less reliable if these codes are not essential for billing. In data collected with EHRs in the UK, for example, data that relate to items detailed in the Quality Outcomes Framework are likely to be better quality if captured after 2004.[36]

<u>Item RCHD-2 (new).</u> *Eligibility criteria for participants in the cohort or routinely collected health database(s).*

Examples:

1) “Patients were eligible for inclusion in the cohort if they were 45 years or older; had a smoking history of at least 10 pack-years; had a clinical diagnosis of mild-to-severe COPD [chronic obstructive pulmonary disease], defined as a postbronchodilator forced expiratory volume in 1s (FEV1) to forced vital capacity ratio of 0.7 or lower and a

For peer review only - http://bmjopen.bmj.com/site/about/guidelines.xhtml

postbronchodilator FEV1 of at least 30%, according to Global Initiative of Chronic Obstructive Lung Disease (GOLD) and American Thoracic Society and European Respiratory Society criteria (GOLD stage 1–3); and had at least one documented or self-reported exacerbation during the past 3 years, with the restriction that the last exacerbation had ended at least 4 weeks before inclusion and symptoms had returned to patients' baseline levels. Exclusion criteria were poor mastery of the Dutch language, poor cognitive functioning, known allergy to doxycycline, pregnancy, and a life expectancy of shorter than 1 month."[37]

2) "Baseline characteristics and clinical outcomes will be extracted from routinely recorded clinical data held in the NNRD [National Neonatal Research Database]. The NNRD holds data from all infants admitted to National Health Service (NHS) neonatal units in England, Scotland and Wales (~90 000 infants annually). Contributing neonatal units are known as the UK Neonatal Collaborative. Data are extracted from point-of-care neonatal electronic health records completed by health professionals during routine clinical care. A defined data extract, the Neonatal Dataset of ~450 data items, is transmitted quarterly to the Neonatal Data Analysis Unit at Imperial College London and Chelsea and Westminster NHS Foundation Trust where patient episodes across different hospitals are linked and data are cleaned (queries about discrepancies and implausible data configurations are fed back to health professionals and rectified)."[38]

Explanation:

Since the cohort or routinely collected health database serves as the sampling frame for the trial, the representativeness of trial participants depends on its eligibility criteria, and a clear description of criteria for entry into the cohort or routinely collected health database should be

For peer review only - http://bmjopen.bmj.com/site/about/guidelines.xhtml

provided.[26] For example, in health administrative data, having insurance (e.g., Medicare in the USA) is a prerequisite for having a record in the database; an RCT with participants recruited from the database could only be representative of people with the insurance coverage.

When using a cohort or routinely collected health database in which eligibility fluctuates over time (e.g., health insurance data), researchers should clearly specify how eligibility was defined and how changes in eligibility over the study period were managed. Additionally, changes in variable coding over time could result in differences in characteristics of participants deemed eligible for RCT enrolment. Therefore, coding changes relevant to characterising cohort or database participants and RCT eligibility criteria and enrolment should be reported.

For peer review only

Item RCHD-3 (new). State whether the study included person-level, institutional-level, or other data linkage across two or more databases and, if so, linkage techniques and methods used to evaluate completeness and accuracy of linkage.

Examples:

1) "Individuals on the Oregon Experiment "reservation list" (N=100,407) were probabilistically matched to individual OCHIN [Oregon Community Health Information Network] patients (N=106,692), using Link Plus software and demographic variables common to both data sets. Two researchers independently performed a case-by-case review of uncertain matches using additional demographic variables. Appendix Table 1 provides more details.…

   Information in Appendix: "To identify individuals common to both the Medicaid reservation list and the OCHIN patient population, we used Link Plus software to probabilistically compare demographic variables contained in both datasets. Matching

For peer review only - http://bmjopen.bmj.com/site/about/guidelines.xhtml

variables included first and last name, date of birth, gender, street address, city, Oregon Medicaid identification number, and preferred language. The software generates a "match score" indicating each pair's likelihood of being a match. For pairs of uncertain match status based on match score, we conducted double clerical review by independent reviewers. We also completed several rounds of quality assurance analyses to verify the validity of our match results."[39]

Explanation:

When databases are linked, investigators need to select a set of variables to be used for linking, determine the best method for linking the databases and develop a linking algorithm, and evaluate the accuracy of linkages between the databases.[40] A description of linkage methods and the success of linkage is critical to permit the reader to assess the likelihood and potential impact of any linkage error and the possibility of related bias.[41] Linkage bias occurs when associations are present between the probability of linkage error (e.g., false and missing matches) and variables of interest. For example, linkage rates may vary by patient characteristics, such as health status or health services received. Even small errors in the linkage process can introduce bias and lead to results that can overestimate or underestimate the associations under study.[42]

Authors should describe if linkage of records across multiple databases was conducted, and if so, the methods of linkage (e.g., deterministic versus probabilistic, quality and type of variables used for linkage), how any linkage validation was done, and results of linkage validation with estimated rate of successful linkage. Details should be provided on blocking variables (variables used to form pairs for comparison only among those with the potential to be matches, such as the first 3 digits of a postal code) completeness of linkage variables, linkage rules, thresholds, and manual review of potential matches, if undertaken.[43,44] If linkage was

For peer review only - http://bmjopen.bmj.com/site/about/guidelines.xhtml
For peer review only

conducted prior to the trial for previous studies or general use or if linkage was undertaken by an external provider, such as a data linkage centre, then a reference describing the data resource and linkage methods should be provided. Authors should report linkage error using standard approaches including comparisons with gold standards or reference datasets, sensitivity analyses, and comparing characteristics of linked and unlinked data.[45]

Trial Participants (modified section subheading)

Item 4a (modified):

CONSORT 2010 item: Eligibility criteria for participants.

Modified CONSORT extension item: Eligibility criteria for trial participants, including information on how to access the list of codes and algorithms used to identify eligible participants, information on accuracy and completeness of data used to ascertain eligibility, and methods used to validate accuracy and completeness (e.g., monitoring, adjudication), if applicable.

Examples:

1) "Primary care physicians were eligible for the study if they practiced in a study clinic, provided care to at least 10 adults with type 2 diabetes, and provided written informed consent to participate. Patients were classified as having diabetes if they had 2 or more out-patient diabetes International Classification of Diseases, Ninth Revision (ICD-9) codes (250.xx) or used 1 or more diabetes-specific medications in the 1-year period before randomization. This diabetes identification method has estimated sensitivity of 0.91 and positive predictive value of 0.94."[46]

For peer review only - http://bmjopen.bmj.com/site/about/guidelines.xhtml

2) "An EHR-based algorithm to identify eligible patients was constructed based on International Classification of Diseases, 9th and 10th Revisions, Clinical Modification codes (67–69) (see Table E1 in the online supplement) that are present on admission. In addition, nurses complete a five-item electronic checklist during intake to denote the disease-specific eligibility criteria. To validate the algorithm, we reviewed 271 medical charts across the participating hospitals. The algorithm identified 171 of these patients as eligible and 100 as ineligible. Using manual chart review as the gold standard, the algorithm had a false-positive rate of 1% and a false-negative rate of 5%."[47]

Explanation:

This section relates to entry into the trial (rather than the cohort or routinely collected health database, which is covered in items RCHD-1 to RCHD-3). When eligible trial participants were identified from records in a cohort or routinely collected health database, authors should report information necessary to evaluate or replicate this process. This should include a clear and detailed description of all codes, algorithms, and free-text field entries or combinations thereof. Ideally, a link to all material (including statistical code) needed for replication should be provided through an appendix or posting to an accessible website.

Use of routinely collected health data may introduce some degree of misclassification bias, and information on the validity of participant classification must be specifically described, including reference to available validation studies and any methods used to directly assess the validity of data used for participant classification and the accuracy of classification. Potential changes that may affect different settings and timepoints should be considered. This could occur, for example, when coding standards or strategies that may affect the validity of the data are changed or when software or algorithms are updated.

For peer review only - http://bmjopen.bmj.com/site/about/guidelines.xhtml

To help readers assess the applicability of trial results, authors should clearly describe potential differences between the trial target population, persons included in the cohort or health database, and actual trial participants. Filtering effects may occur, for instance, when data are more often incomplete in special situations, such as emergency visits (versus routine visits) due to different processes for routine data collection, and if persons with incomplete data are not screened for trial eligibility.

<u>Item 4b (unmodified):</u> *Settings and locations where the data were collected.*

Examples:

1) "The trial was conducted in the area of the Lille-Douai Health Insurance district (Northern France) during the institutional seasonal influenza vaccination campaign of 2014–2015.…In the intervention group, 25 GPs received and were supposed to expose in their waiting rooms 135 pamphlets and one poster (added to the usual mandatory information) withdrawing all the other posters. In the control group, waiting rooms were kept in their usual state.…Data were extracted between October 15, 2014 and February 28, 2015 from the SIAM-ERASME claim database of the Lille-Douai district Health Insurance Fund on patient level."[48]
2) The present study is one of three trials that took place in the context of the PRO-AGE (PRevention in Older people-Assessment in GEneralists' practices) project in three locations. The present study was conducted in Hamburg, Germany, and was intended to test whether HRA-O [health risk appraisal for older persons], combined with personal reinforcement and supplemented.…In Hamburg, general practitioners (GPs) registered in the entire metropolitan area (~500 GPs) were informed via the newsletter of their regional

For peer review only - http://bmjopen.bmj.com/site/about/guidelines.xhtml
For peer review only

GP association (BDA-Landesverband Hamburg).…Survival, nursing home admission, and need for ambulatory nursing care as well as change of residence data were obtained from the GP records and completed with participant and proxy information. At year 1, the HRA-O questionnaire was used for collecting outcome information from all study participants. It was sent to surviving persons in combination with a short questionnaire on self-efficacy in the patient–physician interaction."[49]

Explanation:

Information on the settings and locations where the trial is conducted is key to judge trial applicability and generalizability.[18,19] In trials conducted using cohorts or routinely collected health data, authors should describe where the trial was implemented and specify if there were differences between centres where overall cohort or database data were collected (see item RCHD-2) and those involved in the trial. This may occur if only a subset of centres in the cohort or database are selected randomly or by characteristics, such as data quality reasons, location, health care delivery characteristics, or language. Additionally, centres in a cohort, for example, could be assigned to participate in different ongoing trials occurring simultaneously or in overlapping time periods using the cohort.

<u>*Item RCHD-4 (new):*</u> *Describe whether and how consent was obtained.*

Examples:

1) "At enrollment in the cohort, patients are asked to provide informed consent for prospective collection of clinical, survival and PROMs [patient-reported outcome measures] data.…we ask patients' consent to be randomly selected to receive offers on experimental interventions in the future and to use their data comparatively....Patients

For peer review only - http://bmjopen.bmj.com/site/about/guidelines.xhtml

within the cohort who meet the inclusion criteria form a subcohort of eligible patients….From among this subcohort, a random sample is selected….Randomly selected patients are offered the experimental intervention (boost prior to sCRT) by their treating physician. If they accept the offer, they will sign an additional informed consent to receive the boost. Patients who refuse the boost will receive care as usual (that is, sCRT). Patients in the subcohort who will not be randomly selected will not be informed about the boost intervention, nor will they be informed about their participation in the control arm of this study."[50]

Explanation:

In trials using cohorts and routinely collected health data, informed consent may be applied at different levels and multiple stages for an individual participant, as well as in different ways than in conventional RCT designs where consent is usually obtained just once for treatment, randomization and data use.[51] Reporting the information provided to potential participants and the consents sought will help readers understand what participants knew and what they expected or hoped might happen at each stage of the research, including the trial. Clearly describing this in the text and flow diagram will enable evaluation of applicability of trial results and facilitate replication.

Authors should describe the different types of consent sought and obtained for the cohort or routinely collected health database and the trial. These may include: (1) consent for use of health data for research via a cohort or routinely collected health database; (2) consent to be contacted for future research purposes; (3) prior consent to future randomisation without explicit notice, which often occurs in trials that use the cohort multiple RCT design [4,52]; (4) consent to receive a trial intervention; or (5) conventional consent to trial participation and randomisation.

For peer review only

For peer review only - http://bmjopen.bmj.com/site/about/guidelines.xhtml

Other types of consent may also be relevant, such as consent to no description of experimental intervention if allocated to control, or consent for linkage with other datasets. For each type of consent sought, authors should describe from whom consent was sought, whether consent was sought for all trial participants or only some (e.g., only those allocated to a trial intervention), and when each type of consent sought was sought.

Interventions

<u>*Item 5 (unmodified):*</u> *The interventions for each group with sufficient details to allow replication, including how and when they were actually administered.*

Examples:

1) "We developed a computer-based electronic alert system for identifying consecutive hospitalized OAC [oral-anticoagulation]-naïve patients with AF [arterial fibrillation] and tested the hypothesis that such an alert system would improve OAC prescription. The alert system automatically identified hospitalized patients with AF without an active OAC prescription in the electronic order entry system. The alert system was incorporated into the electronic medical chart and order entry system of the University Hospital in Bern, Switzerland. It recognized AF by permanently searching diagnosis lists and physician notes of the entire electronic patient chart database for free text entries of AF or its various abbreviations. Alerts were issued 24 hours after the onset of hospital stay if….4 criteria for an individual patient were present….Once the criteria were fulfilled, the alert was issued in the electronic patient chart. The alert was visible to physicians and nurses, but only physicians were enabled to respond to the alert…."[53]

For peer review only - http://bmjopen.bmj.com/site/about/guidelines.xhtml

2) “Intervention included a single real-time notification by letter to the patient and by electronic message within the KPSC [Kaiser Permanente Southern California] electronic medical record system to each patient’s primary care provider and asthma specialist (if the patient had previously seen one). The patient letters and physician messages noted excessive SABA [short-acting β2-agonist] dispensing, suggestions for management, and facilitated allergy referral recommendation for those patients without prior asthma specialist care….Controls received KPSC standard asthma care management without research contact….”[54]

Explanation:

Interventions are sometimes delivered via EHR systems or using an administrative database, for instance. Examples provided here describe a clinical decision support tool [53] and a drug alert system [54] embedded within EHRs. Other examples might include reminders or links to a clinical practice guideline when specific disease codes or other patient characteristics (e.g., age, sex) that indicate guideline relevance are entered into an EHR. Authors should report interventions triggered or delivered via an EHR, registry, or administrative database in enough detail for readers to be able to understand the intervention characteristics, to replicate them in other research, and for clinical implementation. The Template for Intervention Description and Replication (TIDieR) provides guidance for reporting of interventions.[55]

Outcomes

Item 6a (modified):

<u>CONSORT 2010 item:</u> Completely defined pre-specified primary and secondary outcome measures, including how and when they were assessed.

For peer review only - http://bmjopen.bmj.com/site/about/guidelines.xhtml

<u>Modified CONSORT extension item:</u> Completely defined pre-specified primary and secondary outcome measures, including how and when they were ascertained and the cohort or routinely collected health database(s) used to ascertain each outcome.

Examples:

1) "A hard CVD [cardiovascular disease] event, the primary outcome, was defined as the occurrence of any of the following events in the medical record or Medicare/Medicaid data between IMPACT [Improving Mood-Promoting Access to Collaborative Treatment] enrollment date and December 31, 2008: a) fatal MI [myocardial infarction] (International Classification of Diseases, 10th Revision codes I21-I22 the first-listed cause of death), b) laboratory evidence of acute MI (creatine kinase-myocardial band isoenzyme value 93.0 ng/ml or troponin value 90.3 K g/l), c) acute MI diagnosis (ICD-9 code 410), d) fatal stroke (International Classification of Diseases, 10th Revision codes I60-I64 the first-listed cause of death), or e) hemorrhagic (ICD-9 codes 430Y432) or nonhemorrhagic (ICD-9 codes 433.01, 433.11, 433.21, 433.31, 433.91, 434.01, 434.11, and 434.91) stroke diagnosis. Secondary outcomes were fatal/nonfatal MI (categories a-c), fatal/nonfatal MIYcardiac enzyme confirmed (categories a and b), fatal/nonfatal stroke (categories d and e), and all-cause mortality. Death dates were extracted from the Medicare data, and causes of death were obtained from death certificates provided by the Indiana State Department of Health….Patients were followed up for a maximum of 7.5 to 9.5 years (median = 8.1 years); however, for cause of death (categories a and d), patients were followed up for a maximum of 5.5 to 7.5 years (median = 6.2 years)."[56]
2) "The trial used the national comprehensive Swedish Web System for Enhancement and Development of Evidence-Based Care in Heart Disease Evaluated According to

For peer review only

Recommended Therapies (SWEDEHEART)….for patient enrollment and data collection….The primary end point was death from any cause within 365 days after randomization, assessed in the intention-to-treat population. Secondary end points included death from any cause within 30 days after randomization, rehospitalization with myocardial infarction, rehospitalization with heart failure, and cardiovascular death…as well as composites of these end points, assessed at 30 days and 365 days….Data on the end points of rehospitalization with heart failure and cardiovascular death are not available from SWEDEHEART and must be obtained from the Swedish National Inpatient and Outpatient Registries. Mortality data were obtained from the Swedish National Population Registry, which includes the vital status of all Swedish citizens. All other variables were obtained from SWEDEHEART, which is monitored on a regular basis. Diagnoses at discharge are listed according to codes from the International Classification of Diseases, 10th Revision (ICD-10). The end of follow-up was December 30, 2016, which was 365 days after the last patient underwent randomization. To allow for any lag in registry reporting, the final database was extracted from SWEDEHEART on February 28, 2017, including data on any linked deaths that occurred through December 30, 2016, and reported in the population registry as of February 14, 2017….No central adjudication or trial-specific patient follow-up was performed."[32]

Explanation:

All primary or secondary outcomes should be identified and defined including how and when measured and the cohort(s) or routinely collected health database(s) from which they were ascertained. Details on the accuracy and validity of outcome data should be described. If

For peer review only - http://bmjopen.bmj.com/site/about/guidelines.xhtml

different databases are used in some sites in the trial, authors should note if outcomes are ascertained differently in these different trial sites.

Since follow-up periods may be considerably longer than recruitment periods, sometimes lasting decades, special attention should be given to potential changes that occur over time that may affect data collection, quality, and completeness. Authors may consider using flow diagrams or special tables to describe these circumstances. A crucial aspect to be considered and carefully reported is any connection between collection of outcomes and trial arms (e.g., detection bias). For example, a comparison of surgery versus non-surgical care should consider that special diagnostic procedures that are routinely done in surgical follow-up visits may not be done in the control group.

<u>Item RCHD-5 (new)</u>: *Information on how to access the list of codes and algorithms used to define or derive the outcomes from the cohort or routinely collected health database(s) used to conduct the trial, information on accuracy and completeness of outcome variables, and methods used to validate accuracy and completeness (e.g., monitoring, adjudication), if applicable.*

Examples:

(a) Information on how to access the list of codes and algorithms used to define or derive the outcomes from the cohort or routinely collected health database(s) used to conduct the trial.

1) "The primary outcomes were whether or not the patient received preventive care services in the post-period: screenings for cervical, breast, and colorectal cancer (fecal occult blood testing and colonoscopy); screenings for diabetes (glucose and hemoglobin A1c [HbA1c]), hypertension, obesity, and smoking; lipid screening; chlamydia testing; and receipt of influenza vaccination. Codes were used based on EHR Meaningful Use Stage 1

For peer review only - http://bmjopen.bmj.com/site/about/guidelines.xhtml

measures. These included ICD-9-CM diagnosis and procedure codes, Current Procedural Terminology and Healthcare Common Procedure Coding System codes, Logical Observation Identifiers Names and Codes, and medication codes. The authors also used relevant code groupings and codes specific to the OCHIN [Oregon Community Health Information Network] EHR, used for Meaningful Use reporting and internal quality improvement initiatives. Appendix Table 2 provides detailed technical specifications and patient eligibility criteria for each measure. (see https://ars.els-cdn.com/content/image/1-s2.0-S0749379715004237-mmc1.pdf)."[39]

(b) information on accuracy and completeness of outcome variables, and methods used to validate accuracy and completeness (e.g., monitoring, adjudication).

1) "Uppsala Clinical Research Center provides manuals, education and technical advice, including a telephone help desk for all users of the registry. The system has error checking routines for range and consistency. Definitions are easily available when data are entered. To ensure the correctness of the data entered a monitor visits about 20 hospitals each year and compares data entered into the SWEDEHEART [Swedish Web System for Enhancement and Development of Evidence-Based Care in Heart Disease Evaluated According to Recommended Therapies] with the information in the patients' records from 30–40 randomly chosen patients in each hospital. When 637 randomly chosen computer forms from 21 hospitals containing 38 121 variables were reviewed in 2007, there was a 96.1% (range: 92.6%-97.4%) agreement."[57, supplement]
2) "If a patient was suspected to have had a clinical end-point event (i.e., death, myocardial infarction, bleeding, or stroke), the patient's health care records were subjected to central

For peer review only - http://bmjopen.bmj.com/site/about/guidelines.xhtml

blinded adjudication to determine the cause of the event according to prespecified criteria."[58]

Explanation:

Trials using cohorts or routinely collected health data may require specific codes or algorithms, such as diagnostic codes, to identify and define outcomes. An EHR query can be performed, for example, with a list of diagnostic codes to identify all patients who have experienced a specific adverse event. An algorithm, or sequence of steps necessary to score or grade an outcome, may also be used. To assess validity and to facilitate reproducibility, the list of codes and algorithms should be provided or linked to an external source within the text or in supplemental material, ideally with the computer code used to reproduce this step.

Cohorts and routinely collected health data are often collected and entered by personnel involved in routine patient care or by non-clinical personnel based on medical record documentation, and level of completeness varies. In addition, procedures for entering data for clinical care or billing may introduce certain biases, and concerns about data completeness and accuracy may arise.[59] Authors should describe data completeness in enough detail for others to be able to evaluate accuracy. Issues concerning misclassification, and any efforts to minimise misclassification, should be reported.

Outcome definitions may vary between cohorts and routinely collected health data and standards commonly used in clinical trials and data fields may be missing. The authors should describe any adjudication of outcomes, if adjudication was blinded to trial allocation, and which outcome definitions were used (e.g. by referring to a separate adjudication protocol).

<u>Item 6b (unmodified)</u>: *Any changes to trial outcomes after the trial commenced, with reasons.*

For peer review only - http://bmjopen.bmj.com/site/about/guidelines.xhtml

See CONSORT 2010.[18,19]

Sample size

Item 7a (unmodified): How sample size was determined.

See CONSORT 2010.[18,19]

Item 7b (unmodified): When applicable, explanation of any interim analyses and stopping guidelines.

See CONSORT 2010.[18,19]

Randomisation

Sequence generation

Item 8a (unmodified): Method used to generate the random allocation sequence.

See CONSORT 2010.[18,19]

Item 8b (unmodified): Type of randomisation; details of any restrictions (such as blocking and block size).

See CONSORT 2010.[18,19]

Allocation concealment mechanism

Item 9 (modified):

For peer review only

For peer review only - http://bmjopen.bmj.com/site/about/guidelines.xhtml

<u>*CONSORT 2010 item:*</u> *Mechanism used to implement the random allocation sequence (such as sequentially numbered containers), describing any steps taken to conceal the sequence until interventions were assigned.*

<u>*Modified CONSORT extension item:*</u> *Mechanism used to implement the random allocation sequence (such as embedding the random allocation sequence within the cohort or routinely collected health database(s)), describing any steps taken to conceal the sequence until interventions were assigned.*

Examples:

1) "The [WithHolding Enteral feeds Around packed red cell Transfusion] WHEAT trial is a randomised controlled, unblinded, multicentre, pilot trial comparing two care pathways.… Infants will be randomised with a 1:1 allocation ratio (using permuted blocks of variable size), stratified within neonatal unit by gestational age at birth and infant sex. Trial processes will be embedded within neonatal EPR [electronic patient record] systems and all outcome data will be extracted from data that are routinely recorded within the existing neonatal EPR systems (BadgerNet and BadgerEPR), and held in the NNRD [National Neonatal Research Database].... Infants will be randomised using an online secure central randomisation system which will be embedded into the existing neonatal EPR systems (BadgerNet and BadgerEPR). Randomisation will occur within the EPR to ensure allocation concealment."[38]

2) "Randomization to be offered versus not offered, the SPIN-HAND intervention will occur at the time of Cohort participants' regular SPIN Cohort assessments. Eligible Cohort participants, based on questionnaire responses, will be randomized automatically as they complete their regular SPIN Cohort assessments using a feature in the SPIN Cohort platform, which provides immediate centralized randomization and, thus, complete allocation sequence concealment."[35]

For peer review only

Explanation:

Using cohorts or routinely collected health data to conduct trials may provide opportunities to embed automated randomisation or selection and allocation algorithms into the cohort or database system to allocate participants to trial arms. This could occur by using an automated process or embedding software within the system that can communicate with an external randomisation system. If such processes are used, authors should provide enough details for the randomisation and allocation concealment processes to be fully understood by readers and to assess how they may influence internal validity.

Implementation

Item 10 (unmodified): Who generated the random allocation sequence, who enrolled participants, and who assigned participants to interventions.

See CONSORT 2010.[18,19]

Blinding

Item 11a (unmodified): If done, who was blinded after assignment to interventions (for example, participants, care providers, those assessing outcomes) and how.

See CONSORT 2010.[18,19]

Item 11b (unmodified): If relevant, description of the similarity of interventions.

See CONSORT 2010.[18,19]

Statistical methods

For peer review only

<u>Item 12a (unmodified):</u> Statistical methods to compare groups for primary and secondary outcomes.

See CONSORT 2010.[18,19]

<u>Item 12b (unmodified):</u> Methods for additional analyses, such as subgroup analyses and adjusted analyses.

See CONSORT 2010.[18,19]

For peer review only

Results

Participant flow (a diagram is strongly recommended)

Item 13a (modified):

<u>CONSORT 2010 item:</u> For each group, the numbers of participants who were randomly assigned, received intended treatment, and were analysed for the primary outcome.

<u>Modified CONSORT extension item:</u> For each group, the number of participants in the cohort or routinely collected health database(s) used to conduct the trial and the numbers screened for eligibility, randomly assigned, offered and accepted interventions (e.g., cohort multiple RCTs), received intended treatment, and analysed for the primary outcome.

Example:

1) "We identified the primary care physicians with the highest antibiotic prescription rates in Switzerland using routinely collected claims data of prescriptions of antibiotics and outpatient consultations collected by SASIS, a data warehouse company of an umbrella organization of Swiss statutory health insurers (Santésuisse). These data are collected by over 60 statutory health insurers covering 64% of the Swiss population (5.1 million

For peer review only - http://bmjopen.bmj.com/site/about/guidelines.xhtml

residents).We included among all board certified primary care physicians the 2900 top antibiotic prescribers (based on prescribed defined daily doses [DDD] per 100 consultations in the year prior to randomization…Of 2900 randomized physicians, all 1450 physicians in the intervention group received the evidence-based guidelines and first feedback information….Of the 1450 physicians, 211 (14.6%) opted out later. We used data from 2814 physicians for the intention-to-treat analysis….”[60] (see Figure 1A)

2) “Upon receiving permission to contact participants from their respective registry site, FHPP [Family Health Promotion Project] staff at the University of Colorado Cancer Center contacted participants to recruit them into the study (n=1,068). Of the 1,068 subjects contacted, 156 were deemed ineligible and 280 refused participation for an overall response rate of 69% (632 of 912 eligible…). The 632 consenting participants, representing 533 families, completed the baseline survey and were randomized to receive either the tailored telephone counseling intervention (N=322) or the general mailed intervention (N=310)…A total of 632 participants were enrolled in the FHPP trial. Of the 322 participants randomized to the telephone intervention, 306 (95%) received the intervention (16 participants could not be reached by phone within the allotted time frame per protocol), and 309 of 310 (>99%) participants in the mailed group received the mailed packet. Retention of participants over 24 months was greater than 90% overall: 87% in the telephone and 94% in the mailed intervention group.”[61] (See Figure 1B)

Explanation:

The number of participants in a cohort or routinely collected health database(s) and the numbers who were screened for eligibility, randomly assigned, offered and accepted interventions (e.g., cohort multiple RCTs), received the intended treatment, and analysed for the

For peer review only - http://bmjopen.bmj.com/site/about/guidelines.xhtml

primary and secondary outcomes should be described. When multiple sources of data were linked, potential exclusions due to data linkage should be specifically described. If persons in a cohort or routinely collected health database who are not included in the trial are observed and data reported, this should be clearly reported and included in the flow chart.

Figure 2 provides an example of a flow chart that might be used to describe the flow of participants into a cohort or routinely collected health database and then into the trial. Specific components to be included depend on the trial design and may include the number of participants in the cohort or routinely collected health database, the number who were not screened for trial eligibility because the recruitment target was met, due to data linkage problems, or because of a lack of consent to be contacted for research purposes, for example. There may also be elements related to intervention access or use. For example, in the cohort multiple RCT design, consent for the intervention is sought post-randomization, in which case the numbers of participants who gave this consent should be reported.

Item 13b (unmodified): *For each group, losses and exclusions after randomisation, together with reasons.*

See CONSORT 2010.[18,19]

Additionally, for trials using cohorts or routinely collected health data, losses and exclusions based on data quality or linkage problems should be specifically described.

Recruitment

Item 14a (unmodified): *Dates defining the periods of recruitment and follow-up.*

Example:

For peer review only - http://bmjopen.bmj.com/site/about/guidelines.xhtml

1) “A parallel group randomised controlled trial (RCT) with 878 participants in the intervention and 1,702 in the control group was performed between 2001-2002.…Briefly, 14 general practitioners with solo practices recruited participants for the RCT over a nine-month period starting in October 2000. Potential participants were identified using complete GP’s patient lists. At baseline (2000/2001), eligible study participants were at least 60 years old…. Eligible individuals received the study information letter from their GPs, the PRA questionnaire (Probability for Repeated Admission) measuring six items of baseline risk status for health service use, i.e., person’s age, gender, hospital admissions, visits to GP, health status (heart disease and diabetes status), and caregiver availability, one question on B-ADL and the informed consent form.”[62]

Explanation:

Participants in a cohort or routinely collected health database are typically followed for an extended period, and the starting date of trial recruitment will often differ from the start date of data collection in the cohort or database. Trials using these types of data may be uniquely positioned to obtain long-term follow-up data. The length of follow-up may be a fixed period after randomisation, but in RCTs in which the outcome is time to an event, follow-up of all participants ends on a specific date. Start and end dates for the trial should be given, and the minimum, maximum, and median duration of follow-up for trials for which the outcome is time to an event should be reported. If subsequent longer-term follow-up subsequent to a trial using an ongoing cohort or database is expected, this should be explained.

<u>Item 14b (unmodified):</u> Why the trial ended or stopped.

For peer review only - http://bmjopen.bmj.com/site/about/guidelines.xhtml

See CONSORT 2010.[18,19]

Baseline data

<u>*Item 15 (unmodified):*</u> *A table showing baseline demographic and clinical characteristics for each group.*

Example:

"During the study period, 11,709 patients with STEMI [ST-segment elevation myocardial infarction] in Sweden and Iceland underwent PCI and were registered in SCAAR [Swedish Coronary Angiography and Angioplasty Registry]. Of these, 7012 were enrolled in the trial. An additional 247 patients were enrolled from the center in Denmark, for a total of 7259 patients…. Fifteen erroneous enrollments (patients initially reported as having STEMI, for whom the diagnosis was changed by the operator and no PCI was performed) were excluded from the database, leaving 7244 patients who underwent randomization. The baseline clinical characteristics of all the patients who underwent randomization (including patients at all the centers) and all the patients who did not undergo randomization (including patients at all the centers except the center in Denmark) are listed in Table 1."[63]

Explanation:

A feature of RCTs using cohorts and routinely collected health data is that baseline data of participants not enrolled in the trial is usually more likely to be available. Figure 3 shows the table from the example. Baseline characteristics for eligible persons from the cohort or routinely collected health database who were not eligible for the trial due to missing data or other administrative reasons, or who declined participation should be reported in the same way, to the

For peer review only - http://bmjopen.bmj.com/site/about/guidelines.xhtml

extent possible, as for the randomised trial participants. Analyses that evaluate differences at trial entry between non-participants and those randomised can inform the representativeness of trial participants.

Numbers analysed

<u>*Item 16 (unmodified):*</u> *For each group, number of participants (denominator) included in each analysis and whether the analysis was by original assigned groups.*

See CONSORT 2010.[18,19]

Outcomes and estimation

<u>*Item 17a (unmodified):*</u> *For each primary and secondary outcome, results for each group, and the estimated effect size and its precision (such as 95% confidence interval).*

See CONSORT 2010.[18,19]

<u>*Item 17b (unmodified):*</u> *For binary outcomes, presentation of both absolute and relative effect sizes is recommended.*

See CONSORT 2010.[18,19]

Ancillary analyses

<u>*Item 18 (unmodified):*</u> *Results of any other analyses performed, including subgroup analyses and adjusted analyses, distinguishing pre-specified from exploratory.*

See CONSORT 2010.[18,19]

For peer review only

Harms

<u>*Item 19 (unmodified):*</u> *All important harms or unintended effects in each group (for specific guidance see CONSORT for harms).*

See CONSORT 2010.[18,19]

Discussion

Limitations

<u>*Item 20 (unmodified):*</u> *Trial limitations, addressing sources of potential bias, imprecision, and, if relevant, multiplicity of analyses.*

Examples:

1) "A number of limitations of the TASTE trial should be noted. First, the treating physician was aware of the group to which the patient had been assigned, and that physician entered the angiographic variables into the registry; therefore, these variables were susceptible to bias. Second, we did not adjudicate events and did not review angiograms in a blinded fashion. We used all-cause death as the primary end point as it is the most stringent end point and because of the completeness of the national death registries in each participating country. We chose not to perform separate adjudication of secondary end points both to limit expense and because of the high reliability of the SWEDEHEART [Swedish Web System for Enhancement and Development of Evidence-Based Care in Heart Disease Evaluated According to Recommended Therapies] registry…. A comparison of the clinical characteristics and outcomes between the patients who underwent randomization and those who did not indicates that the two cohorts differed significantly in a number of respects .... Even when a trial uses a population-based

For peer review only - http://bmjopen.bmj.com/site/about/guidelines.xhtml

registry for enrollment, the trial participants cannot be fully representative of the complete range of patients."[63]

2) "Awareness of the trial might have itself promoted better data recording [in the EHR]. Nevertheless, we observed several limitations of the data including, for example, a high proportion of patients with unspecified subtype of stroke and a smaller number with BP [blood pressure] values not recorded during the intervention period. From an explanatory perspective, these limitations of the data reduce the capacity of the study to provide an accurate assessment of intervention efficacy."[34]

Explanation:

As per the CONSORT 2010 statement, the identification and discussion of the potential limitations of a trial is crucial to appropriate interpretation of trial results, including issues such as potential bias, imprecision and multiplicity of comparisons. Unique characteristics of trials using cohorts or routinely collected health data may be linked to risk of bias and associated problems and, therefore, need specific attention in the discussion, including issues such as data availability, problems with data linkage, data validation, and data quality [51]. The Clinical Trials Transformation Initiative [64] has similarly identified that problems with the relevance, reliability or reproducibility of data within registries or with other routinely collected data can influence trial conduct and results.

Generalisability

<u>*Item 21 (unmodified):*</u> *Generalisability (external validity, applicability) of the trial findings.*

1) "A comparison of the clinical characteristics and outcomes between the patients who underwent randomization and those who did not indicates that the two cohorts differed

For peer review only

For peer review only - http://bmjopen.bmj.com/site/about/guidelines.xhtml

significantly in a number of respects, most notably in mortality at 30 days (2.9% among patients who underwent randomization vs. 10.6% among those who did not). In many cases, these differences reflect the exclusion from the trial of patients who were ineligible because they were unable to provide oral consent. Even when a trial uses a population-based registry for enrollment, the trial participants cannot be fully representative of the complete range of patients."[63]

Explanation:

Careful attention should be paid to how participants in an ongoing cohort or with records in a routinely collected health database may differ from the population targeted by the trial, and these differences and their relevance for the interpretation of the trial findings should be discussed. Additionally, any trial design decisions related to intervention delivery or outcome collection that were influenced by using a cohort or routinely collected health database should be considered. An advantage of many trials conducted in cohorts or using routinely collected health data is that information on participants not included in the trial is available. An assessment of the degree to which trial participants differ from non-participants by reason of non-participation can provide readers with insight on representativeness. Possible risks to generalizability that are identified, and their potential implications should be discussed.

Interpretation

Item 22 (modified):

CONSORT 2010 item: *Interpretation consistent with results, balancing benefits and harms, and considering other relevant evidence.*

For peer review only - http://bmjopen.bmj.com/site/about/guidelines.xhtml

Modified CONSORT extension item: Interpretation consistent with results, balancing benefits and harms, and considering other relevant evidence, including the implications of using data that were not collected to answer the specific research question.

Examples:

1) "Using the EHR as a sole source of patient data is a limitation. For example, the EHR did not capture the patient experience of the intervention, including its potential impact on pain control, function, and disability. Furthermore, EHR data do not provide accurate substance use and mental health diagnoses. We did not have prescription or visit data from outside health systems."[65]

Explanation:

Authors should report whether and how the use of cohort or routinely collected health data may be a limitation of the trial. These limitations could include, amongst others, the choice of outcome measures based on availability in the cohort or routinely collected health database and the quality and accuracy of outcome data. Where possible, results should be compared with evidence from similar RCTs using a conventional design and differences that may be related to the use of a cohort or routinely collected health data should be discussed.

Other information

Registration

Item 23 (unmodified): Registration number and name of trial registry.

Protocol

Item 24 (unmodified): Where the full trial protocol can be accessed, if available.

For peer review only - http://bmjopen.bmj.com/site/about/guidelines.xhtml

Examples:

1) "This trial used the platform of preexisting health care registries for enrollment, randomization, collection of data, and follow-up (for further details, see the Supplementary Appendix, available at NEJM.org)."[57]

Explanation:

As per CONSORT 2010, trials should be registered, and their protocol should be accessible. When a trial is being conducted using a cohort or routinely collected health database, in addition to the trial protocol, the authors should ideally provide a link to the protocol for the cohort or routinely collected health database, if separate. This allows interested readers to better understand the characteristics of cohort or database participants and data collection methods.

Funding

Item 25 (modified):

CONSORT 2010 item: Sources of funding and other support (such as supply of drugs), role of funders.

Modified CONSORT extension item: Sources of funding and other support for both the trial and the cohort or routinely collected health database(s), role of funders.

Example

1) "The registry is financed by the Swedish government and the Association of Local Authorities and Regions (the public health care provider), and is supported by the Swedish Heart Association, the National Board of Health and Welfare and the Swedish Heart and Lung Foundation. Participating hospitals are not reimbursed by the registry and

For peer review only

costs of local data entry are borne by their internal budget [32, supplement]. The trial sponsor was the Karolinska Institutet."[32]

Explanation:

In addition to providing the funding source for the trial, authors should also report any funding sources of the cohort or routinely collected health data and if they had any involvement in the use of the cohort or dataset in the trial or in the trial itself.

CONCLUSIONS

This extension of the CONSORT reporting guideline for trials using cohorts and routinely collected health data is a minimum set of items to inform readers about the trial design and its findings and to support informed decisions about the validity of trial results and applicability to readers' research questions. The extension addresses only aspects of trial reporting specific to trials using cohorts and routinely collected health data. When reporting a trial using cohorts or routinely collected health data, authors should address all items on the CONSORT checklist by using this document in conjunction with the main CONSORT 2010 guidelines. Authors should also consult other CONSORT extensions that are relevant to their trial design, such as extensions for cluster trials,[66] pragmatic trial designs,[67] or others. All are available online at www.consort-statement.org/extensions. Authors are also encouraged to report any additional information, specific to their trial, that would assist readers to more easily evaluate trial results or to replicate trial methods.

In addition to assisting authors of trial reports, this CONSORT extension aims to promote transparency and clarity and to reduce research waste due to poor reporting. We encourage journal editors to direct authors of trials using cohorts and routinely collected health data to use

For peer review only - http://bmjopen.bmj.com/site/about/guidelines.xhtml

this checklist and to document adherence to reporting recommendations as a condition of manuscript submission.

For peer review only

For peer review only - http://bmjopen.bmj.com/site/about/guidelines.xhtml

ACKNOWLEDGMENTS

Author Contributions:

LK, OF, LGH, MZ, CR, SML, MSampson, DM, CG, EJ, and BDT were responsible for the study conception and design. MI, SJM, KAMcC, DBR, EIB, LT, MKC, DE, HMV, IB, PR, JN, RU, MSauvé, JF and DT provided regular feedback on each step in the development process. LK, MI, SJM, OF, LGH, MZ, CR, DBR, SML, EIB, LT, MKC, DE, CG, EJ and BDT contributed to drafting the manuscript. All authors provided a critical review and approved the final manuscript. BDT and LK are the guarantors.

Funding: The development of the CONSORT extension for RCTs conducted using cohorts and routinely collected health data was funded by grants from the Canadian Institutes of Health Research (PI Thombs, Co-Is Frobert, Relton, Kwakkenbos, Juszczak, #PJT-156172; PI Thombs, Co-I Kwakkenbos, #PCS-161863) and from the United Kingdom National Institute of Health Research (NIHR) Clinical Trials Unit Support Funding (PI Juszczak, Co-PI Gale, supported salary of SM). The views expressed are those of the authors and not necessarily those of the NHS, the NIHR or the Department of Health and Social Care. Ms. Rice was supported by a Vanier CIHR Graduate Scholarship. Dr. Langan was supported by a Wellcome Senior Clinical Fellowship in Science (205039/Z/16/Z). Dr. Benchimol was supported by a New Investigator Award from the Canadian Institutes of Health Research, Canadian Association of Gastroenterology and Crohn's and Colitis Canada. He was also supported by the Career Enhancement Program of the Canadian Child Health Clinician Scientist Program. Dr. Moher was supported by a University Research Chair (uOttawa). Dr. Uher was supported by the Canada Research Chairs Program (Award #231397). Dr. Gale was supported by the United Kingdom

For peer review only - http://bmjopen.bmj.com/site/about/guidelines.xhtml

Medical Research Council through a Clinician Scientist Fellowship. Dr. Thombs was supported by a Tier 1 Canada Research Chair.

Competing interests: The authors have read and understood the BMJ policy on declaration of interests and declare that they have no competing interests.

Copyright for author: The Corresponding Author has the right to grant on behalf of all authors and does grant on behalf of all authors, a worldwide licence (http://www.bmj.com/sites/default/files/BMJ%20Author%20Licence%20March%202013.doc) to the Publishers and its licensees in perpetuity, in all forms, formats and media (whether known now or created in the future), to i) publish, reproduce, distribute, display and store the Contribution, ii) translate the Contribution into other languages, create adaptations, reprints, include within collections and create summaries, extracts and/or, abstracts of the Contribution and convert or allow conversion into any format including without limitation audio, iii) create any other derivative work(s) based in whole or part on the on the Contribution, iv) to exploit all subsidiary rights to exploit all subsidiary rights that currently exist or as may exist in the future in the Contribution, v) the inclusion of electronic links from the Contribution to third party material where-ever it may be located; and, vi) licence any third party to do any or all of the above. All research articles will be made available on an open access basis (with authors being asked to pay an open access fee—see http://www.bmj.com/about-bmj/resources-authors/forms-policies-and-checklists/copyright-open-access-and-permission-reuse). The terms of such open access shall be governed by a Creative Commons licence—details as to which Creative Commons licence will apply to the research article are set out in our worldwide licence referred to above.

For peer review only

Availability of data and materials: Additional data beyond that reported in the main and supplementary materials can be requested from the corresponding author.

For peer review only

For peer review only - http://bmjopen.bmj.com/site/about/guidelines.xhtml

REFERENCES

1. Torgerson D, Torgerson C. Designing randomised trials. Basingstoke: Palgrave; 2008.

2. Schwartz D, Lellouch J. Explanatory and pragmatic attitudes in therapeutical trials. *J Chron Dis* 1967;20:637–48.

3. Evans I, Thornton H, Chalmers I, et al. Testing Treatments: Better research for better healthcare. London: Pinter and Martin Ltd; 2011.

4. Relton C, Torgerson D, O'Cathain A, et al. Rethinking pragmatic randomised controlled trials: introducing the "cohort multiple randomised controlled trial" design. *BMJ* 2010;340:2.

5. Treweek S, Mitchell E, Pitkethly M, et al. Strategies to improve recruitment to randomised controlled trials. *Cochrane Database Syst Rev* 2010;4:MRMR000013.

6. Watson J, Torgerson D. Increasing recruitment to randomised trials: a review of randomised controlled trials. *BMC Med Res Methodol* 2006;6:34.

7. Campbell M, Snowdon C, Francis D, et al. Recruitment to randomised trials: strategies for trial enrolment and participation study: the STEPS study. *Health Technol Assess* 2007;11:48 iii, ix-105.

8. Treweek S, Lockhart P, Pitkethly M, et al. Methods to improve recruitment to randomised controlled trials: Cochrane systematic review and meta-analysis. *BMJ Open* 2013;3:e002360.

9. Sully BG, Julious SA, Nicholl J. A reinvestigation of recruitment to randomised, controlled, multicenter trials: a review of trials funded by two UK funding agencies. *Trials* 2013;14:166.

For peer review only

10. McDonald AM, Treweek S, Shakur H, et al. Using a business model approach and marketing techniques for recruitment to clinical trials. *Trials* 2011;12:74.

11. Donovan JL, Paramasivan S, De Salis I, et al. Clear obstacles and hidden challenges: understanding recruiter perspectives in six pragmatic randomised controlled trials. *Trials* 2014;15:5.

12. Paul SM, Mytelka DS, Dunwiddie CT, et al. How to improve R&D productivity: the pharmaceutical industry's grand challenge. *Nat Rev Drug Discov* 2010;9:203-14.

13. Spasoff RA. Epidemiologic Methods for Health Policy. New York: Oxford University Press, Inc.; 1999.

14. Sherman RE, Anderson SA, Dal Pan GJ, et al. Real-world evidence - what is it and what can it tell us? *N Engl J Med* 2016; 375:2293-7.

15. James S, Fröbert O, Lagerqvist B. Cardiovascular registries: a novel platform for randomised clinical trials. *Heart* 2012;98:1329-31.

16. van Staa TP, Dyson L, McCann G, et al. The opportunities and challenges of pragmatic point-of-care randomised trials using routinely collected electronic records: evaluations of two exemplar trials. *Health Technol Assess* 2014;18:1-146.

17. Anderson GL, Burns CJ, Larsen J, et al. Use of administrative data to increase the practicality of clinical trials: insights from the Women's Health Initiative. *Clin Trials* 2016;13:519-26.

18. Schulz KF, Altman DG, Moher D, et al. CONSORT 2010 statement: updated guidelines for reporting parallel group randomised trials. *BMJ* 2010 23;340:c332.

19. Moher D, Hopewell S, Schulz KF, et al. CONSORT 2010 explanation and elaboration: updated guidelines for reporting parallel group randomised trials. *BMJ* 2010;340:c869.

For peer review only

20. Kwakkenbos L, Juszczak E, Hemkens LG, et al. Protocol for the development of a CONSORT extension for RCTs using cohorts and routinely collected health data. *Res Integr Peer Rev* 2018;3:9.

21. Reporting guidelines under development: The EQUATOR Network. http://www.equator-network.org/library/reporting-guidelines-under-development/. Accessed November 2, 2020.

22. Moher D, Schulz KF, Simera I, et al. Guidance for developers of health research reporting guidelines. *PLoS Med* 2010;7:e1000217.

23. Kwakkenbos L, Imran M, McCord KA, et al. Protocol for a scoping review to support development of a CONSORT extension for randomised controlled trials using cohorts and routinely collected health data. *BMJ Open* 2018;8:e025266.

24. Imran M, Kwakkenbos L, McCall S, et al. Methods used in the development of a consensus-driven extension to the Consolidated Standards of Reporting Trials (CONSORT) Statement for Trials Conducted Using Cohorts and Routinely Collected Health Data. Under review by *BMJ*.

25. von Elm E, Altman DG, Egger M, et al. The Strengthening the Reporting of Observational Studies in Epidemiology (STROBE) Statement: guidelines for reporting observational studies. *BMJ*. 2007;335(7624):806-808.

26. Benchimol EI, Smeeth L, Guttmann A, et al. The REporting of studies Conducted using Observational Routinely-collected health Data (RECORD) Statement. *PLoS Med* 2015;12:e1001885.

27. Erlinge D, Koul S, Eriksson P, et al. Bivalirudin versus heparin in non-ST and ST-segment elevation myocardial infarction-a registry-based randomized clinical trial in the

For peer review only

For peer review only - http://bmjopen.bmj.com/site/about/guidelines.xhtml

SWEDEHEART registry (the VALIDATE-SWEDEHEART trial). *Am Heart J* 2016;175:36-46.

28. Cockayne S, Rodgers S, Green L, et al. Clinical effectiveness and cost-effectiveness of a multifaceted podiatry intervention for falls prevention in older people: a multicentre cohort randomised controlled trial (the REducing Falls with ORthoses and a Multifaceted podiatry intervention trial). *Health Technol Assess* 2017;21:1-198.

29. Åsberg S, Hijazi Z, Norrving B, et al. Timing of oral anticoagulant therapy in acute ischemic stroke with atrial fibrillation: study protocol for a registry-based randomised controlled trial. *Trials* 2017;18:581.

30. Levine DA, Funkhouser EM, Houston TK, et al. Improving care after myocardial infarction using a 2-year internet-delivered intervention: the Department of Veterans Affairs myocardial infarction-plus cluster-randomized trial. *Arch Intern Med* 2011;171:1910-7.

31. Hopewell S, Clarke M, Moher D, et al. CONSORT for reporting randomized controlled trials in journal and conference abstracts: explanation and elaboration. *PLoS Med*. 2008;5:e20.

32. Hofmann R, James SK, Jernberg T, et al. Oxygen Therapy in Suspected Acute Myocardial Infarction. *N Engl J Med* 2017;377:1240-9.

33. Vollmer WM, Owen-Smith AA, Tom JO, et al. Improving adherence to cardiovascular disease medications with information technology. *Am J Manag Care* 2014;20:SP502-SP510

34. Dregan A, van Staa TP, McDermott L, et al. Point-of-care cluster randomized trial in stroke secondary prevention using electronic health records. *Stroke* 2014;45:2066-71.

For peer review only

35. Carrier ME, Kwakkenbos L, Boutron I, et al. Randomized feasibility trial of the Scleroderma Patient-centered Intervention Network hand exercise program (SPIN-HAND): study protocol. *J Scleroderma Relat Disord* 2017;3:91-97.

36. Campbell S, Reeves D, Kontopantelis E, et al. Effects of pay for performance on the quality of primary care in England. *N Engl J Med* 2009;361:368-78.

37. van Velzen P, Ter Riet G, Bresser P, et al. Doxycycline for outpatient-treated acute exacerbations of COPD: a randomised double-blind placebo-controlled trial. *Lancet Respir Med* 2017;5:492-9.

38. Gale C, Modi N, Jawad S, et al. The WHEAT pilot trial—WithHolding Enteral feeds Around packed red cell Transfusion to prevent necrotising enterocolitis in preterm neonates: a multicentre, electronic patient record (EPR), randomised controlled point-of-care pilot trial. *BMJ Open* 2019;9:e033543.

39. Marino M, Bailey SR, Gold R, et al. Receipt of Preventive Services After Oregon's Randomized Medicaid Experiment. *Am J Prev Med* 2016;50:161-70.

40. Bradley CJ, Penberthy L, Devers KJ, et al. Health services research and data linkages: issues, methods, and directions for the future. *Health Serv Res* 2010;45:1468-88.

41. Bohensky MA, Jolley D, Sundararajan V, et al. Data linkage: a powerful research tool with potential problems. *BMC Health Serv Res* 2010;10:346.

42. Harron K, Wade A, Muller-Pebody B, et al. Opening the black box of record linkage. *J Epidemiol Community Health* 2012;66:1198.

43. Harron K, Goldstein H, Wade A, et al. Linkage, evaluation and analysis of national electronic healthcare data: application to providing enhanced blood-stream infection surveillance in paediatric intensive care. *PloS one* 2013;8:e85278.

For peer review only

44. Shlomo N. Overview of Data Linkage Methods for Policy Design and Evaluations. In: Crato N., Paruolo P. (eds) Data-Driven Policy Impact Evaluation. Springer, Cham.; 2019.

45. Lariscy JT. Differential record linkage by Hispanic ethnicity and age in linked mortality studies: implications for the epidemiologic paradox. *J Aging Health* 2011;23:1263-84.

46. O'Connor PJ, Sperl-Hillen JM, Rush WA, et al. Impact of electronic health record clinical decision support on diabetes care: a randomized trial. *Ann Fam Med* 2011;9:12-21.

47. Courtright KR, Madden V, Gabler NB, et al. Rationale and Design of the Randomized Evaluation of Default Access to Palliative Services (REDAPS) Trial. *Ann Am Thorac Soc* 2016;13:1629-39.

48. Berkhout C, Willefert-Bouche A, Chazard E, et al. Randomized controlled trial on promoting influenza vaccination in general practice waiting rooms. *PloS one* 2018;13: e0192155.

49. Dapp U, Anders JA, von Renteln-Kruse W, et al. A randomized trial of effects of health risk appraisal combined with group sessions or home visits on preventive behaviors in older adults. *J Gerontol A Biol Sci Med Sci* 2011;66:591-8.

50. Burbach JP, Verkooijen HM, Intven M, et al. RandomizEd controlled trial for pre-operAtive dose-escaLation BOOST in locally advanced rectal cancer (RECTAL BOOST study): study protocol for a randomized controlled trial. *Trials* 2015;16:58.

51. Mc Cord K, Al-Shahi Salman R, Treweek S, et al. Routinely collected data for randomized trials: promises, barriers, and implications. *Trials* 2018;19:29.

52. Young-Afat DA, Verkooijen HA, van Gils CH, et al. Staged-informed Consent in the Cohort Multiple Randomized Controlled Trial Design. *Epidemiology* 2016;27:389-92.

For peer review only

53. Silbernagel G, Spirk D, Hager A, et al. Electronic Alert System for Improving Stroke Prevention Among Hospitalized Oral-Anticoagulation-Naïve Patients With Atrial Fibrillation: A Randomized Trial. *J Am Heart Assoc* 2016;5:e003776.

54. Zeiger RS, Schatz M, Li Q, et al. Real-time asthma outreach reduces excessive short-acting β2-agonist use: a randomized study. *J Allergy Clin Immunol Pract* 2014;2:445-6.

55. Hoffmann TC, Glasziou PP, Boutron I, et al. Better reporting of interventions: template for intervention description and replication (TIDieR) checklist and guide. *BMJ* 2014;348:g1687.

56. Stewart JC, Perkins AJ, Callahan CM. Effect of collaborative care for depression on risk of cardiovascular events: data from the IMPACT randomized controlled trial. *Psychosom Med* 2014;76:29-37.

57. Erlinge D, Omerovic E, Fröbert O, et al. Bivalirudin versus Heparin Monotherapy in Myocardial Infarction. *N Engl J Med* 2017;377:1132-42.

58. Lagerqvist B, Fröbert O, Olivecrona GK, et al. Outcomes 1 year after thrombus aspiration for myocardial infarction. *N Engl J Med* 2014;371:1111-20.

59. Funk MJ, Landi SN. Misclassification in administrative claims data: quantifying the impact on treatment effect estimates. *Curr Epidemiol Rep* 2014;1:175-85.

60. Hemkens LG, Saccilotto R, Reyes SL, et al. Personalized Prescription Feedback Using Routinely Collected Data to Reduce Antibiotic Use in Primary Care: A Randomized Clinical Trial. *JAMA Intern Med* 2017;177:176-83.

61. Lowery JT, Horick N, Kinney AY, et al. A randomized trial to increase colonoscopy screening in members of high-risk families in the colorectal cancer family registry and cancer genetics network. *Cancer Epidemiol Biomarkers Prev* 2014;23:601-10.

For peer review only

62. Neumann L, Dapp U, von Renteln-Kruse W, et al. Health Promotion and Preventive Care Intervention for Older Community-Dwelling People: Long-Term Effects of a Randomised Controlled Trial (RCT) within the LUCAS Cohort. *J Nutr Health Aging* 2017;21:1016-23.

63. Fröbert O, Lagerqvist B, Olivecrona GK, et al. Thrombus aspiration during ST-segment elevation myocardial infarction. *N Engl J Med* 2013;369:1587-97.

64. Clinical Trials Transformation Initiative. CTTI Recommendations: Registry trials. https://www.ctti-clinicaltrials.org/files/recommendations/registrytrials-recs.pdf. Accessed November 2, 2020.

65. Liebschutz JM, Xuan Z, Shanahan CW, et al. Improving Adherence to Long-term Opioid Therapy Guidelines to Reduce Opioid Misuse in Primary Care: A Cluster-Randomized Clinical Trial. *JAMA Intern Med* 2017;177:1265-72.

66. Campbell MK, Piaggio G, Elbourne DR, et al. CONSORT 2010 statement: extension to cluster randomised trials. *BMJ* 2012;345:e5661.

67. Zwarenstein M, Treweek S, Gagnier JJ, et al. Improving the reporting of pragmatic trials: an extension of the CONSORT statement. *BMJ* 2008;337:a2390.

For peer review only

Table 1: Checklist for Reporting of Trials Conducted using Cohorts or Routinely Collected Health Data: Comparison of the Extension to the CONSORT 2010 Statement

Section/Topic<br><br>(New Section Headings and Modifications to Headings are in Red)	Item No.	CONSORT 2010 Checklist Item	Extension for Trials Conducted using Cohorts or Routinely Collected Health Data
Title and abstract			
	1a	Identification as a randomised trial in the title	
	1b	Structured summary of trial design, methods, results, and conclusions (for specific guidance see CONSORT for abstracts)	Structured summary of trial design, methods, results, and conclusions (for specific guidance see CONSORT for abstracts). Specify that a cohort or routinely collected health data were used to conduct the trial and, if applicable, provide the name of the cohort or routinely collected health database(s) (Modified)
Introduction			
Background and objectives	2a	Scientific background and explanation of rationale	
	2b	Specific objectives or hypotheses	
Methods			
Trial design	3a	Description of trial design (such as parallel, factorial) including allocation ratio	Description of trial design (such as parallel, factorial) including allocation ratio, that a cohort or routinely collected health database(s) was used to conduct the trial (such as electronic health record, registry) and how the data were used within the trial (such as identification of eligible trial participants, trial outcomes) (Modified)
	3b	Important changes to methods after trial commencement (such as eligibility criteria), with reasons	

For peer review only

Cohort or routinely collected health database	RCHD-1		Name, if applicable, and description of the cohort or routinely collected health database(s) used to conduct the trial, including information on the setting (such as primary care), locations, and dates, (such as periods of recruitment, follow-up, and data collection) (New)
	RCHD-2		Eligibility criteria for participants in the cohort or routinely collected health database(s) (New)
	RCHD-3		State whether the study included person-level, institutional-level, or other data linkage across two or more databases and, if so, linkage techniques and methods used to evaluate completeness and accuracy of linkage (New)
Trial participants	4a	Eligibility criteria for participants	Eligibility criteria for trial participants, including information on how to access the list of codes and algorithms used to identify eligible participants, information on accuracy and completeness of data used to ascertain eligibility, and methods used to validate accuracy and completeness (e.g., monitoring, adjudication), if applicable (Modified)
	4b	Settings and locations where the data were collected	
	RCHD-4		Describe whether and how consent was obtained (New)
Interventions	5	The interventions for each group with sufficient details to allow replication, including how and when they were actually administered	
Outcomes	6a	Completely defined pre-specified primary and secondary outcome measures, including how and when they were assessed	Completely defined pre-specified primary and secondary outcome measures, including how and when they were ascertained and the cohort or routinely collected health database(s) used to ascertain each outcome (Modified)
	RCDH-5		Information on how to access the list of

For peer review only - http://bmjopen.bmj.com/site/about/guidelines.xhtml

			codes and algorithms used to define or derive the outcomes from the cohort or routinely collected health database(s) used to conduct the trial, information on accuracy and completeness of outcome variables, and methods used to validate accuracy and completeness (e.g., monitoring, adjudication), if applicable (New)
	6b	Any changes to trial outcomes after the trial commenced, with reasons	
Sample size	7a	How sample size was determined	
	7b	When applicable, explanation of any interim analyses and stopping guidelines	
Randomisation:			
Sequence generation	8a	Method used to generate the random allocation sequence	
	8b	Type of randomisation; details of any restriction (such as blocking and block size)	
Allocation concealment mechanism	9	Mechanism used to implement the random allocation sequence (such as sequentially numbered containers), describing any steps taken to conceal the sequence until interventions were assigned	Mechanism used to implement the random allocation sequence (such as embedding an automated randomiser within the cohort or routinely collected health database(s)), describing any steps taken to conceal the sequence until interventions were assigned (Modified)
Implementation	10	Who generated the random allocation sequence, who enrolled participants, and who assigned participants to interventions	
Blinding	11a	If done, who was blinded after assignment to interventions (for example, participants, care providers, those assessing outcomes) and how	
	11b	If relevant, description of the similarity of interventions	
Statistical methods	12a	Statistical methods used to compare groups for primary and secondary outcomes	

For peer review only

	12b	Methods for additional analyses, such as subgroup analyses and adjusted analyses	
Results			
Participant flow (a diagram is strongly recommended)	13a	For each group, the numbers of participants who were randomly assigned, received intended treatment, and were analysed for the primary outcome	For each group, the number of participants in the cohort or routinely collected health database(s) used to conduct the trial and the numbers screened for eligibility, randomly assigned, offered and accepted interventions (e.g., cohort multiple RCTs), received intended treatment, and analysed for the primary outcome (Modified)
	13b	For each group, losses and exclusions after randomisation, together with reasons	
Recruitment	14a	Dates defining the periods of recruitment and follow-up	
	14b	Why the trial ended or was stopped	
Baseline data	15	A table showing baseline demographic and clinical characteristics for each group	
Numbers analysed	16	For each group, number of participants (denominator) included in each analysis and whether the analysis was by original assigned groups	
Outcomes and estimation	17a	For each primary and secondary outcome, results for each group, and the estimated effect size and its precision (such as 95% confidence interval)	
	17b	For binary outcomes, presentation of both absolute and relative effect sizes is recommended	
Ancillary analyses	18	Results of any other analyses performed, including subgroup analyses and adjusted analyses, distinguishing pre-specified from exploratory	

For peer review only

For peer review only - http://bmjopen.bmj.com/site/about/guidelines.xhtml

Harms	19	All important harms or unintended effects in each group (for specific guidance see CONSORT for harms)	
Discussion			
Limitations	20	Trial limitations, addressing sources of potential bias, imprecision, and, if relevant, multiplicity of analyses	
Generalisability	21	Generalisability (external validity, applicability) of the trial findings	
Interpretation	22	Interpretation consistent with results, balancing benefits and harms, and considering other relevant evidence	Interpretation consistent with results, balancing benefits and harms, and considering other relevant evidence, including the implications of using data that were not collected to answer the trial research questions (Modified)
Other information			
Registration	23	Registration number and name of trial registry	
Protocol	24	Where the full trial protocol can be accessed, if available	
Funding	25	Sources of funding and other support (such as supply of drugs), role of funders	Sources of funding and other support for both the trial and the cohort or routinely collected health database(s), role of funders (Modified)

**Section/Topic**	**Item No.**	**CONSORT 2010 Checklist Item**	**Extension for Trials Conducted using Cohorts or Routinely Collected Health Data**
**(New Section Headings and Modifications to Headings are in Red)**			
Title and abstract			
	1a	Identification as a randomised trial in the title	
	1b	Structured summary of trial design, methods, results, and conclusions (for specific guidance see CONSORT for	Structured summary of trial design, methods, results, and conclusions (for specific guidance see CONSORT for

For peer review only

		abstracts)	abstracts). Specify that a cohort or routinely collected health data were used to conduct the trial and, if applicable, provide the name of the cohort or routinely collected health database(s) (Modified)
Introduction			
Background and objectives	2a	Scientific background and explanation of rationale	
	2b	Specific objectives or hypotheses	
Methods			
Trial design	3a	Description of trial design (such as parallel, factorial) including allocation ratio	Description of trial design (such as parallel, factorial) including allocation ratio, that a cohort or routinely collected health database(s) was used to conduct the trial (such as electronic health record, registry) and how the data were used within the trial (such as identification of eligible trial participants, trial outcomes) (Modified)
	3b	Important changes to methods after trial commencement (such as eligibility criteria), with reasons	
Cohort or routinely collected health database	RCHD-1		Name, if applicable, and description of the cohort or routinely collected health database(s) used to conduct the trial, including information on the setting (such as primary care), locations, and dates, (such as periods of recruitment, follow-up, and data collection) (New)
	RCHD-2		Eligibility criteria for participants in the cohort or routinely collected health database(s) (New)
	RCHD-3		State whether the study included person-level, institutional-level, or other data linkage across two or more databases and, if so, linkage techniques and methods used to evaluate completeness and accuracy of linkage (New)
Trial participants	4a	Eligibility criteria for participants	Eligibility criteria for trial participants, including information on how to access

For peer review only

			the list of codes and algorithms used to identify eligible participants, information on accuracy and completeness of data used to ascertain eligibility, and methods used to validate accuracy and completeness (e.g., monitoring, adjudication), if applicable (Modified)
	4b	Settings and locations where the data were collected	
	RCHD-4		Describe whether and how consent was obtained (New)
Interventions	5	The interventions for each group with sufficient details to allow replication, including how and when they were actually administered	
Outcomes	6a	Completely defined pre-specified primary and secondary outcome measures, including how and when they were assessed	Completely defined pre-specified primary and secondary outcome measures, including how and when they were ascertained and the cohort or routinely collected health database(s) used to ascertain each outcome (Modified)
	RCDH-5		Information on how to access the list of codes and algorithms used to define or derive the outcomes from the cohort or routinely collected health database(s) used to conduct the trial, information on accuracy and completeness of outcome variables, and methods used to validate accuracy and completeness (e.g., monitoring, adjudication), if applicable (New)
	6b	Any changes to trial outcomes after the trial commenced, with reasons	
Sample size	7a	How sample size was determined	
	7b	When applicable, explanation of any interim analyses and stopping guidelines	
Randomisation:			

For peer review only

Sequence generation	8a	Method used to generate the random allocation sequence	
	8b	Type of randomisation; details of any restriction (such as blocking and block size)	
Allocation concealment mechanism	9	Mechanism used to implement the random allocation sequence (such as sequentially numbered containers), describing any steps taken to conceal the sequence until interventions were assigned	Mechanism used to implement the random allocation sequence (such as embedding an automated randomiser within the cohort or routinely collected health database(s)), describing any steps taken to conceal the sequence until interventions were assigned (Modified)
Implementation	10	Who generated the random allocation sequence, who enrolled participants, and who assigned participants to interventions	
Blinding	11a	If done, who was blinded after assignment to interventions (for example, participants, care providers, those assessing outcomes) and how	
	11b	If relevant, description of the similarity of interventions	
Statistical methods	12a	Statistical methods used to compare groups for primary and secondary outcomes	
	12b	Methods for additional analyses, such as subgroup analyses and adjusted analyses	
Results			
Participant flow (a diagram is strongly recommended)	13a	For each group, the numbers of participants who were randomly assigned, received intended treatment, and were analysed for the primary outcome	For each group, the number of participants in the cohort or routinely collected health database(s) used to conduct the trial and the numbers screened for eligibility, randomly assigned, offered and accepted interventions (e.g., cohort multiple RCTs), received intended treatment, and analysed for the primary outcome (Modified)

For peer review only - http://bmjopen.bmj.com/site/about/guidelines.xhtml

	13b	For each group, losses and exclusions after randomisation, together with reasons	
Recruitment	14a	Dates defining the periods of recruitment and follow-up	
	14b	Why the trial ended or was stopped	
Baseline data	15	A table showing baseline demographic and clinical characteristics for each group	
Numbers analysed	16	For each group, number of participants (denominator) included in each analysis and whether the analysis was by original assigned groups	
Outcomes and estimation	17a	For each primary and secondary outcome, results for each group, and the estimated effect size and its precision (such as 95% confidence interval)	
	17b	For binary outcomes, presentation of both absolute and relative effect sizes is recommended	
Ancillary analyses	18	Results of any other analyses performed, including subgroup analyses and adjusted analyses, distinguishing pre-specified from exploratory	
Harms	19	All important harms or unintended effects in each group (for specific guidance see CONSORT for harms)	
Discussion			
Limitations	20	Trial limitations, addressing sources of potential bias, imprecision, and, if relevant, multiplicity of analyses	
Generalisability	21	Generalisability (external validity, applicability) of the trial findings	
Interpretation	22	Interpretation consistent with results, balancing benefits and harms, and considering other relevant evidence	Interpretation consistent with results, balancing benefits and harms, and considering other relevant evidence, including the implications of using data

For peer review only

For peer review only - http://bmjopen.bmj.com/site/about/guidelines.xhtml

			that were not collected to answer the trial research questions (Modified)
Other information			
Registration	23	Registration number and name of trial registry	
Protocol	24	Where the full trial protocol can be accessed, if available	
Funding	25	Sources of funding and other support (such as supply of drugs), role of funders	Sources of funding and other support for both the trial and the cohort or routinely collected health database(s), role of funders (Modified)

For peer review only

Table 2: Checklist for Reporting of Trials Conducted using Cohorts or Routinely Collected Health Data

Section/Topic	Item No.	CONSORT Extension for Trials Conducted using Cohorts or Routinely Collected Health Data Item	Reported on page #
Title and abstract			
	1a	Identification as a randomised trial in the title	
	1b	Structured summary of trial design, methods, results, and conclusions (for specific guidance see CONSORT for abstracts). Specify that a cohort or routinely collected health data were used to conduct the trial and, if applicable, provide the name of the cohort or routinely collected health database(s)	
Introduction			
Background and objectives	2a	Scientific background and explanation of rationale	
	2b	Specific objectives or hypotheses	
Methods			
Trial design	3a	Description of trial design (such as parallel, factorial) including allocation ratio, that a cohort or routinely collected health database(s) was used to conduct the trial (such as electronic health record, registry) and how the data were used within the trial (such as identification of eligible trial participants, trial outcomes)	
	3b	Important changes to methods after trial commencement (such as eligibility criteria), with reasons	
Cohort or routinely collected health database	RCHD-1	Name, if applicable, and description of the cohort or routinely collected health database(s) used to conduct the trial, including information on the setting (such as primary care), locations, and dates, (such as periods of recruitment, follow-up, and data collection)	
	RCHD-2	Eligibility criteria for participants in the cohort or routinely collected health database(s)	
	RCHD-3	State whether the study included person-level, institutional-level, or other data linkage across two or more databases and, if so, linkage techniques and methods used to evaluate completeness and accuracy of linkage	
Trial participants	4a	Eligibility criteria for trial participants, including information on how to access the list of codes and algorithms used to identify eligible participants, information on accuracy and completeness of data used to ascertain eligibility, and methods used to validate accuracy and completeness (e.g., monitoring,	

For peer review only

		adjudication), if applicable	
	4b	Settings and locations where the data were collected	
	RCHD-4	Describe whether and how consent was obtained	
Interventions	5	The interventions for each group with sufficient details to allow replication, including how and when they were actually administered	
Outcomes	6a	Completely defined pre-specified primary and secondary outcome measures, including how and when they were ascertained and the cohort or routinely collected health database(s) used to ascertain each outcome	
	RCDH-5	Information on how to access the list of codes and algorithms used to define or derive the outcomes from the cohort or routinely collected health database(s) used to conduct the trial, information on accuracy and completeness of outcome variables, and methods used to validate accuracy and completeness (e.g., monitoring, adjudication), if applicable	
	6b	Any changes to trial outcomes after the trial commenced, with reasons	
Sample size	7a	How sample size was determined	
	7b	When applicable, explanation of any interim analyses and stopping guidelines	
Randomisation:			
Sequence generation	8a	Method used to generate the random allocation sequence	
	8b	Type of randomisation; details of any restriction (such as blocking and block size)	
Allocation concealment mechanism	9	Mechanism used to implement the random allocation sequence (such as embedding an automated randomiser within the cohort or routinely collected health database(s)), describing any steps taken to conceal the sequence until interventions were assigned	
Implementation	10	Who generated the random allocation sequence, who enrolled participants, and who assigned participants to interventions	
Blinding	11a	If done, who was blinded after assignment to interventions (for example, participants, care providers, those assessing outcomes) and how	

For peer review only

	11b	If relevant, description of the similarity of interventions	
Statistical methods	12a	Statistical methods used to compare groups for primary and secondary outcomes	
	12b	Methods for additional analyses, such as subgroup analyses and adjusted analyses	
Results			
Participant flow (a diagram is strongly recommended)	13a	For each group, the number of participants in the cohort or routinely collected health database(s) used to conduct the trial and the numbers screened for eligibility, randomly assigned, offered and accepted interventions (e.g., cohort multiple RCTs), received intended treatment, and analysed for the primary outcome	
	13b	For each group, losses and exclusions after randomisation, together with reasons	
Recruitment	14a	Dates defining the periods of recruitment and follow-up	
	14b	Why the trial ended or was stopped	
Baseline data	15	A table showing baseline demographic and clinical characteristics for each group	
Numbers analysed	16	For each group, number of participants (denominator) included in each analysis and whether the analysis was by original assigned groups	
Outcomes and estimation	17a	For each primary and secondary outcome, results for each group, and the estimated effect size and its precision (such as 95% confidence interval)	
	17b	For binary outcomes, presentation of both absolute and relative effect sizes is recommended	
Ancillary analyses	18	Results of any other analyses performed, including subgroup analyses and adjusted analyses, distinguishing pre-specified from exploratory	
Harms	19	All important harms or unintended effects in each group (for specific guidance see CONSORT for harms)	
Discussion			
Limitations	20	Trial limitations, addressing sources of potential bias, imprecision, and, if relevant, multiplicity of analyses	
Generalisability	21	Generalisability (external validity, applicability) of the trial findings	

For peer review only

Interpretation	22	Interpretation consistent with results, balancing benefits and harms, and considering other relevant evidence, including the implications of using data that were not collected to answer the trial research questions	
Other information			
Registration	23	Registration number and name of trial registry	
Protocol	24	Where the full trial protocol can be accessed, if available	
Funding	25	Sources of funding and other support for both the trial and the cohort or routinely collected health database(s), role of funders	

For peer review only

For peer review only - http://bmjopen.bmj.com/site/about/guidelines.xhtml

Box 1: Key methodological issues and considerations in trials conducted using cohorts and routinely collected health data

Design

- Trials conducted using cohorts or routinely collected health databases may differ from conventional trial designs by using these sources of data for identification of eligible participants; automated randomisation, intervention delivery; data collection including outcome assessment, or a combination of these functions.
- Some trials may use a hybrid approach that integrates use of the source of data and trial-specific methods for functions such as intervention delivery and outcome assessment.
- Cohorts and routinely collected health databases can vary substantially in the degree to which they represent complete, random, or convenience samples. Since the cohort or routinely collected health database may serve as the sampling frame for the trial, the representativeness of trial participants may depend on database characteristics.
- The comprehensiveness, collection procedures, and type of demographic or outcome data available in a cohort or routinely collected health database may influence design of the trial, including the research question, trial eligibility criteria and the choice of outcomes.
- The timing between eligibility assessment, intervention delivery, and outcome assessment may be governed by the frequency of data collection in a cohort or routinely collected health database and less under the control of trial investigators than in conventional trials.
- In trials using cohorts and routinely collected health data, informed consent may be applied at different levels and ways compared to conventional trial designs. Consent may be sought and obtained to use the cohort or routinely collected health database and for the

w only

trial, and consent that would typically be expected to occur in conventional trials may not be done due to features of the integrated cohort or database and trial design.

Conduct

- Since cohorts, registries, electronic health records, and administrative databases vary in the degree to which they are set up for research, clinical care, or financial and administrative purposes, the completeness and accuracy of data may vary substantially, both between different databases and between variables within a single database.
- There may be challenges in linking routinely collected health data to other sources of data, including linkage errors when records cannot be linked or are linked incorrectly.

Analysis

- A unique feature of trials using cohorts and routinely collected health data is that investigators can often access information on participants not enrolled in the trial. Differences in baseline characteristics of eligible persons from the cohort or routinely collected health database who do not participate in the trial can often be compared to trial participants to inform judgements on representativeness of trial participants and generalizability of results.

Interpretation

- Applicability of trial results depends on potential differences between the trial target population, persons included in the cohort or routinely collected health database, and trial participants, and this should be considered when interpreting the findings.
- Although there are advantages to using a cohort or routinely collected health data for a trial, there are also limitations, such as constraints on available outcome measures and

For peer review only

issues with data linkage, data validation, and data quality that could influence trial eligibility and outcome assessments.

For peer review only

FIGURE LEGENDS

Figure 1A. Example of participant flowchart for item 13a [60]

Figure 1B. Example of participant flowchart for item 13a [61]

Figure 2. Example flow diagram for trials conducted using cohorts or routinely collected health data

Figure 3. Example of table comparing baseline characteristics of participants in the trial and those who were not randomised for item 15 [63]

For peer review only

BMJ Open

Methods and Results Used in the Development of a Consensus-driven Extension to the Consolidated Standards of Reporting Trials (CONSORT) Statement for Trials Conducted Using Cohorts and Routinely Collected Data (CONSORT-ROUTINE)

Journal:	*BMJ Open*
Manuscript ID	bmjopen-2021-049093.R1
Article Type:	Original research
Date Submitted by the Author:	16-Mar-2021

Complete List of Authors:	Imran, Mahrukh; Lady Davis Institute for Medical Research, Psychiatry<br>Kwakkenbos, Linda; Lady Davis Institute for Medical Research, Psychiatry<br>McCall, Stephen; University of Oxford, National Perinatal Epidemiology Unit Clinical Trials Unit, Nuffield Department of Population Health; American University of Beirut, Center for Research on Population and Health, Faculty of Health Sciences<br>McCord, Kimberly; University of Basel, Basel Institute for Clinical Epidemiology and Biostatistics, Department of Clinical Research, University Hospital Basel<br>Frobert, Ole; Orebro University, Faculty of Health, Department of Cardiology<br>Hemkens, Lars; University of Basel, Basel Institute for Clinical Epidemiology and Biostatistics, Department of Clinical Research, University Hospital Basel<br>Zwarenstein, Merrick; Western University, Department of Family Medicine<br>Relton, Clare ; Queen Mary University of London, Institute of Population Health<br>Rice, Danielle; McGill University, ; Ottawa Hospital Research Institute,<br>Langan, Sinead; London School of Hygiene & Tropical Medicine, Faculty of Epidemiology and Population Health<br>Benchimol, Eric; Children's Hospital of Eastern Ontario, Gastroenterology, Hepatology and Nutrition<br>Thabane, Lehana ; McMaster University, Department of Health Research Methods, Evidence, and Impact<br>Campbell, Marion; University of Aberdeen, Health Services Research Unit<br>Sampson, Margaret; Children's Hospital of Eastern Ontario, Library Services<br>Erlinge, David; Lund University, Department of Cardiology, Clinical Sciences<br>Verkooijen, Helena; University of Utrecht Faculty of Medicine<br>Moher, David; Ottawa Hospital Research Institute, Ottawa Methods Centre<br>Boutron, Isabelle; INSERM<br>Ravaud, Philippe; INSERM<br>Nicholl, Jon; University of Sheffield, Medical Care Research Unit

	Uher, Rudolf; Dalhousie University, Psychiatry<br>Sauve, Maureen; Scleroderma Canada<br>Fletcher, John; BMJ<br>Torgerson, David; University of York, Health Sciences<br>Gale, Chris; Imperial College London, Medicine<br>Juszczak, Edmund; National Perinatal Epidemiology Unit Clinical Trials Unit, Nuffield Department of Population Health, University of Oxford<br>Thombs, Brett; Lady Davis Institute for Medical Research

**Primary Subject Heading**:	Medical publishing and peer review
Secondary Subject Heading:	Evidence based practice, Epidemiology
Keywords:	STATISTICS & RESEARCH METHODS, GENERAL MEDICINE (see Internal Medicine), Clinical trials < THERAPEUTICS

SCHOLARONE™
Manuscripts

I, the Submitting Author has the right to grant and does grant on behalf of all authors of the Work (as defined in the below author licence), an exclusive licence and/or a non-exclusive licence for contributions from authors who are: i) UK Crown employees; ii) where BMJ has agreed a CC-BY licence shall apply, and/or iii) in accordance with the terms applicable for US Federal Government officers or employees acting as part of their official duties; on a worldwide, perpetual, irrevocable, royalty-free basis to BMJ Publishing Group Ltd ("BMJ") its licensees and where the relevant Journal is co-owned by BMJ to the co-owners of the Journal, to publish the Work in this journal and any other BMJ products and to exploit all rights, as set out in our licence.

The Submitting Author accepts and understands that any supply made under these terms is made by BMJ to the Submitting Author unless you are acting as an employee on behalf of your employer or a postgraduate student of an affiliated institution which is paying any applicable article publishing charge ("APC") for Open Access articles. Where the Submitting Author wishes to make the Work available on an Open Access basis (and intends to pay the relevant APC), the terms of reuse of such Open Access shall be governed by a Creative Commons licence – details of these licences and which Creative Commons licence will apply to this Work are set out in our licence referred to above.

Other than as permitted in any relevant BMJ Author's Self Archiving Policies, I confirm this Work has not been accepted for publication elsewhere, is not being considered for publication elsewhere and does not duplicate material already published. I confirm all authors consent to publication of this Work and authorise the granting of this licence.

For peer review only

Methods and Results Used in the Development of a Consensus-driven Extension to the Consolidated Standards of Reporting Trials (CONSORT) Statement for Trials Conducted Using Cohorts and Routinely Collected Data (CONSORT-ROUTINE)

Mahrukh Imran[1]; Linda Kwakkenbos[2]; Stephen McCall[3,4,5]; Kimberly A. Mc Cord[6]; Ole Fröbert[7]; Lars G. Hemkens[6]; Merrick Zwarenstein[8,9]; Clare Relton[10]; Danielle B. Rice[1,11]; Sinéad M. Langan[12]; Eric I. Benchimol[13,14,15]; Lehana Thabane[16]; Marion K. Campbell[17]; Margaret Sampson[18]; David Erlinge[19]; Helena M. Verkooijen[20,21]; David Moher[22]; Isabelle Boutron[23,24,25]; Philippe Ravaud[23,24,25]; Jon Nicholl[26]; Rudolf Uher[27]; Maureen Sauvé[28,29]; John Fletcher[,30]; David Torgerson[31]; Chris Gale[32]; Edmund Juszczak[3,33]; Brett D. Thombs[1,34]

[1]Lady Davis Institute for Medical Research, Jewish General Hospital, Montreal, Canada; [2]Behavioural Science Institute, Clinical Psychology, Radboud University, Nijmegen, the Netherlands; [3]National Perinatal Epidemiology Unit Clinical Trials Unit, Nuffield Department of Population Health, University of Oxford, Oxford, United Kingdom; [4]Center for Research on Population and Health, Faculty of Health Sciences, American University of Beirut, Ras Beirut, Lebanon; [5]Institute of Applied Health Sciences, School of Medicine, Medical Sciences and Nutrition, University of Aberdeen; [6]Basel Institute for Clinical Epidemiology and Biostatistics, Department of Clinical Research, University Hospital Basel, University of Basel, Basel, Switzerland; [7]Örebro University, Faculty of Health, Department of Cardiology, Örebro, Sweden; [8]Department of Family Medicine, Western University, London, Ontario, Canada; [9]IC/ES Western, London, Ontario, Canada; [10]Centre for Clinical Trials and Methodology, Barts Institute of Population Health Science, Queen Mary University, London, United Kingdom; [11]Department

For peer review only - http://bmjopen.bmj.com/site/about/guidelines.xhtml

of Psychology, McGill University, Montreal, Quebec, Canada; [12]Faculty of Epidemiology and Population Health, London School of Hygiene and Tropical Medicine, London, United Kingdom; [13]Department of Pediatrics and School of Epidemiology and Public Health, University of Ottawa, Ottawa, Canada; [14]ICES uOttawa, Ottawa, Ontario, Canada; [15]Children's Hospital of Eastern Ontario Research Institute, Ottawa, Ontario, Canada; [16]Department of Health Research Methods, Evidence, and Impact, McMaster University, Hamilton, Ontario, Canada; [17]Health Services Research Unit, University of Aberdeen, Aberdeen, United Kingdom; [18]Library Services, Children's Hospital of Eastern Ontario, Ottawa, Canada; [19]Department of Cardiology, Clinical Sciences, Lund University, Lund, Sweden; [20]University Medical Center Utrecht, Utrecht, the Netherlands; [21]University of Utrecht, Utrecht, the Netherlands; [22]Centre for Journalology, Clinical Epidemiology Program, Ottawa Hospital Research Institute, Ottawa, Ontario, Canada; [23]INSERM, Paris, France; [24]Centre d'Épidémiologie Clinique, Hôpital Hôtel Dieu, Assistance Publique–Hôpitaux de Paris, Paris, France; [25]Faculté de Médecine, Université Paris Descartes, Sorbonne Paris Cité, Paris, France; [26]School of Health and Related Research, University of Sheffield, Sheffield, United Kingdom; [27]Department of Psychiatry, Dalhousie University, Halifax, Canada; [28]Scleroderma Society of Ontario, Hamilton, Ontario, Canada; [29]Scleroderma Canada, Hamilton, Ontario, Canada; [30]British Medical Journal, London, United Kingdom; [31]York Trials Unit, Department of Health Sciences, University of York, York, United Kingdom; [32]Neonatal Medicine, School of Public Health, Faculty of Medicine, Imperial College London, Chelsea and Westminster campus, London, United Kingdom; [33]Nottingham Clinical Trials Unit, University of Nottingham, Nottingham, United Kingdom.

For peer review only

For peer review only - http://bmjopen.bmj.com/site/about/guidelines.xhtml

[34]Departments of Psychiatry; Epidemiology, Biostatistics and Occupational Health; Medicine; and Educational and Counselling Psychology; and Biomedical Ethics Unit, McGill University, Montreal, Quebec, Canada.

Address for Correspondence: Brett D. Thombs, PhD; Jewish General Hospital; 4333 Cote Ste Catherine Road; Montreal, Quebec H3T 1E4; Tel (514) 340-8222 ext. 25112; E-mail: brett.thombs@mcgill.ca

For peer review only

ABSTRACT

Objectives: Randomised controlled trials (RCTs) conducted using cohorts and routinely collected data, including registries, electronic health records and administrative databases, are increasingly used in health care intervention research. A Consolidated Standards of Reporting Trials (CONSORT) statement extension for trials conducted using cohorts and routinely collected data (CONSORT-ROUTINE) has been developed with the goal of improving reporting quality. This article describes the processes and methods used to develop the extension and decisions made to arrive at the final checklist.

Methods: The development process involved 5 stages: (1) identification of the need for a reporting guideline and project launch; (2) conduct of a scoping review to identify possible modifications to CONSORT 2010 checklist items and possible new extension items; (3) a 3-round modified Delphi Study involving key stakeholders to gather feedback on the checklist; (4) a consensus meeting to finalise items to be included in the extension, followed by stakeholder piloting of the checklist; and (5) publication, dissemination and implementation of the final checklist.

Results: 27 items were initially developed and rated in Delphi Round 1, 13 items were rated in Round 2 and 11 items were rated in Round 3. Response rates for the Delphi Study were 92 of 125 (74%) invited participants in Round 1, 77 of 92 (84%) Round 1 completers in Round 2, and 62 of 77 (81%) Round 2 completers in Round 3. Twenty-seven members of the project team representing a variety of stakeholder groups attended the in-person consensus meeting. The final checklist includes 5 new items and 8 modified items. The extension Explanation & Elaboration document further clarifies aspects that are important to report.

For peer review only - http://bmjopen.bmj.com/site/about/guidelines.xhtml

Conclusion: Uptake of CONSORT-ROUTINE and accompanying Explanation & Elaboration document will improve conduct of trials, as well as the transparency and completeness of reporting of trials conducted using cohorts and routinely collected data.

Keywords: administrative data, cohort, CONSORT, electronic health records, electronic medical records, registries, reporting guideline, routinely collected data

For peer review only

Strengths and Limitations of this study

- We followed a 5-step process to develop CONSORT-ROUTINE, consistent with EQUATOR guidance.
- Items were informed by reporting guidelines on similar research designs, a scoping review, a 3-round Delphi process, and expert members of the guideline development team.
- CONSORT-ROUTINE was reviewed and tested at various stages of the development by project team members and key stakeholders.
- The limited methodological literature on trials conducted using cohorts and routinely collected data was a limitation in developing the extension.
- Similar to other reporting guidelines, CONSORT-ROUTINE will require re-evaluation and revisions over time to ensure that it is kept up to date with evolving methodology and practice of trials using cohorts and routinely collected data.

For peer review only - http://bmjopen.bmj.com/site/about/guidelines.xhtml

For peer review only

BACKGROUND

The use of reporting guidelines, including the Consolidated Standards of Reporting Trials (CONSORT) statement, improves the transparency and completeness of reports of results from randomised controlled trials (RCTs).[1-4] The CONSORT statement helps to facilitate critical appraisal and interpretation of RCTs by providing guidance to authors on a minimal set of items that should be reported for all trials.[5] The CONSORT 2010 guideline aimed to improve the reporting of two-arm parallel group RCTs. Extensions of the CONSORT statement have been developed to encourage better reporting of other trial designs, including, for instance, multi-arm parallel group randomised trials, cluster trials, pilot and feasibility trials, and pragmatic trials.[6-9]

There is a growing interest in RCTs conducted using cohorts or routinely collected data, including registries, electronic health records (EHRs), and administrative databases.[10-14] In a cohort, a group of individuals is gathered for the purpose of conducting research, whereas routinely collected data refer to data initially collected for purposes other than research or without specific a priori research questions developed before collection.[15,16] Trials may use a cohort or routinely collected data for (1) identification of eligible participants, (2) outcome ascertainment, (3) to implement an intervention, or for a combination of these purposes. For example, in registry-based RCTs, a registry could be used to identify eligible participants for a trial, for the collection of participant baseline characteristics, and as the source of outcome data; some registries have used interactive technology to actively flag participants for RCT enrollment as patient data are entered into the registry.[12] In some EHR trials, the EHR itself is used to implement an intervention. For example, one RCT tested an intervention to reduce antibiotic prescribing by feeding back personalized antibiotic prescription data to primary care physicians.[17]

For peer review only - http://bmjopen.bmj.com/site/about/guidelines.xhtml

The use of cohorts and routinely collected data may make RCTs easier and more feasible to perform by reducing cost, time and other resources.[18,19] It may also facilitate the conduct of trials that more closely replicate real-world clinical practice. These trial designs, however, are relatively recent innovations, and published RCT reports may not describe important aspects of their methodology in a standardised way. Trials conducted using cohorts and routinely collected data share certain elements with conventional RCTs, but there are also distinctive elements to report which are not covered in the CONSORT 2010 statement. The REporting of studies Conducted using Observational Routinely-collected Data (RECORD) statement provides guidance on reporting of studies conducted using routinely collected data but does not address RCT-specific methodological and reporting considerations.[20] Research conducted using routinely collected data presents unique methodological challenges that are often insufficiently reported, but there is scant guidance on methods and reporting of trials conducted using routinely collected data or cohorts.[21,22]

An extension to the CONSORT statement for RCTs conducted using cohorts and routinely collected data was developed using methods recommended for developing reporting guidelines.[23] This article describes, in detail, the consensus-based development process. The main aims of this article are to: (1) describe the methods and processes used in the development of the CONSORT Extension for Trials Conducted Using Cohorts and Routinely Collected Data (CONSORT-ROUTINE; Kwakkenbos et al., under review) and (2) describe decisions made to arrive at the final checklist and the accompanying Explanation and Elaboration statement.

METHODS

The project was registered with the Enhancing the QUAlity and Transparency Of health Research (EQUATOR) network.[24] We followed the EQUATOR network's guidelines for

For peer review only - http://bmjopen.bmj.com/site/about/guidelines.xhtml

recommended methods and processes for developing, disseminating, and implementing health care reporting guidelines.[23] These methods have been used in the development of other similar EQUATOR guidelines. Figure 1 illustrates the 5 parts of the development process for this guideline:

Project Phase 1: Project Launch, Establishment of Team, and Funding

Need for the guideline and literature review: An initial informal review of reports of published protocols and reports of trials using cohorts and routinely collected data by BDT and LK suggested that there appeared to be deficiencies in reporting of such trials. For instance, many reports did not adequately describe the cohort or database from which trial participants were recruited, processes used to link participants across databases were not always provided, and it was sometimes unclear whether trial outcomes were assessed by the trialists or ascertained via existing databases used to conduct the trial. A review of the EQUATOR website and published literature indicated that there was no existing reporting guideline for these types of trials. The RECORD statement addresses reporting issues related to routinely collected data but does not include guidance on reporting of trials. Many trials conducted using routinely collected data are pragmatic or use cluster designs, for instance, but CONSORT extensions for those types of trials do not address issues germane to the use of cohorts or routinely collected data to conduct trials.[7,9]

Project launch and identification of CONSORT-ROUTINE project members: Initial discussions on developing a CONSORT extension for RCTs conducted using cohorts occurred in November 2016 at the Trials within Cohorts symposium in London, United Kingdom (LK, MZ, CR, BDT).[25] Discussions continued virtually and key people involved in cohort-embedded trials or the EQUATOR network were approached during December 2016 (HMV, DM, IB, PR, JN,

For peer review only - http://bmjopen.bmj.com/site/about/guidelines.xhtml

RU, DT). It was suggested that trials conducted in registries had many characteristics similar to those in cohorts, and there was agreement to include registry-based trials in the extension. People with expertise in registry-based trials were approached in March 2017 (OF, LT, MKC, DE), and an experienced librarian (MSampson) and patient representative familiar with trials conducted using cohorts (MSauvé) were also included in the group at that point.

The project was registered on the EQUATOR website in April 2017. During the preparatory phase, while developing searches and reviewing example publications, we became aware that trials conducted using EHRs and administrative databases also shared similar characteristics with trials in cohorts and registries, and it was decided to expand the scope to trials conducted using cohorts and routinely collected data. In July 2017, trialists, who were leading the development of a reporting guideline for EHRs, joined the project group (EJ, CG). Given the relevance of their previous work and their expertise (LGH, SML, DM, EIB) authors who had been involved in the development of the RECORD statement were invited to join the team.[20] Several doctoral students also joined the project team (SM, KAM, and DBR). A steering committee comprising of 10 members with key expertise for consultation was established. A research coordinator (MI) was hired in April 2018 to manage the project, and an experienced journal editor was invited to join (JF). The group communicated regularly throughout the process via a number of virtual meetings, using an online platform to conduct teleconferences, as well as through email discussions.

Rationale for developing one checklist versus 4 different checklists for trials conducted using cohorts, registries, EHRs, and administrative databases: Team members discussed the advantages and disadvantages of creating individual checklists for each of the 4 types of data versus a single checklist for all 4. It was determined that, although there are some differences in

For peer review only - http://bmjopen.bmj.com/site/about/guidelines.xhtml

the implementation of trials across the different types of data sources, the methodological principles are similar, and there is substantial overlap in the design, conduct and factors that may influence interpretability. Thus, the steering committee reached consensus to develop a single statement, addressing any differences by including "if applicable" to items in the checklist that may not apply to all trial designs, and to clarify differences in the Explanation & Elaboration publication as deemed necessary.

Funding: The project team obtained its main source of funding from a grant from the Canadian Institutes of Health Research Institutes (CIHR) to support the development of the guideline (BDT, OF, EJ, LK, CR; Grant #PJT-156172). EJ and CG also obtained funding from the United Kingdom National Institute of Health Research Clinical Trials Unit Support Funding - Supporting efficient / innovative delivery of NIHR research. In addition, funding to hold the face-to-face meeting was provided by a Planning and Dissemination Grant from CIHR (BDT, LK; Grant #PCS - 161863) and by contributions from Queen Mary University of London, the University of Sheffield, McGill University, and the Lady Davis Institute for Medical Research of the Jewish General Hospital in Montreal, Canada.

A project protocol was developed and published.[22]

Project Phase 2: Scoping Review

A preliminary "long list" of possible reporting items was formulated by LK and KAM based on review of the CONSORT 2010 statement items, the Strengthening the Reporting of Observational Studies in Epidemiology (STROBE)[26] and the RECORD statements,[20] as well as discussions with steering committee members. The STROBE and RECORD statements were considered the most relevant to this project because of their focus on reporting of observational studies and non-interventional studies using routinely collected data.

For peer review only

A scoping review was conducted to identify: (1) articles on the methodology or reporting of RCTs conducted using cohorts or routinely collected data that could inform the development of new items or modification of existing CONSORT items; (2) trial reports to identify aspects of reporting that need improvement and examples of good reporting of potential checklist items that could be used to support CONSORT-ROUTINE.[27] We searched for relevant articles on trials conducted using cohorts, registries, EHRs, and administrative databases from 2007 to 2018. After screening articles for inclusion and exclusion at the abstract and full-text level, 10 people from the team independently reviewed the included papers and provided suggestions for modifications or additional reporting guideline items until no new ideas emerged (saturation). Suggestions were added in a standardized, shared spreadsheet. At the same time, team members provided examples of good reporting for each proposed item or item modification. Additionally, the review helped us to create a list of authors with experience in these trial designs as potential participants for the Delphi study. Search terms used in the scoping review are shown in Supplementary File 1.

Project Phase 3: Delphi Study

The objectives of our Delphi study were (a) to obtain feedback on the importance of including each candidate item in CONSORT-ROUTINE; (b) to improve the wording of items considered important; and (c) to elicit suggestions for additional items not in the existing list. We aimed to engage key stakeholders across different sectors and backgrounds. There are not fixed guidelines on the sample size of Delphi studies, and the ideal number of participants may depend on the complexity of the topic, the likely heterogeneity of relevant experiences and viewpoints, and resources available to manage the data generated.[28-30] Many studies use small groups of experts (e.g., < 20), but we believed that a larger group with diverse expertise would best

For peer review only - http://bmjopen.bmj.com/site/about/guidelines.xhtml

complement the knowledge of the project team. Thus, we sent out an invitation to reporting guideline developers (including those involved in previous CONSORT extensions), funders, journal editors, patient representatives, trial methodologists, epidemiologists, meta-research authors, ethicists, biostatisticians and clinical trialists who were identified by members of the project team. We also encouraged recipients of the invitation to forward the invitation to other potentially interested stakeholders.

The Delphi surveys were built and hosted using an online survey platform in Qualtrics®. During registration, we gathered demographic and professional background characteristics of participants, including geographical location, self-identified stakeholder group (e.g., clinical trials user, clinical trialist, methodologist), employment sector, years of experience in trials research, and research experience in trials conducted using cohorts or routinely collected data.

Registered participants received a link to access each of the 3 rounds of the Delphi survey. In each round, we asked participants to rate their perceptions about the importance of each suggested reporting item by ranking items based on how essential they are for reporting on a 1-5 Likert scale (1 = not essential; 5 = essential). There is not consensus on the ideal number of Likert categories or groupings for decision-making, but it is common to use between 4-point and 7-point scales.[29]

Responses were categorized as follows:

1 to 2 = low score (item should not be part of CONSORT-ROUTINE checklist);

3 = moderate (item should be discussed);

4 to 5 = high score (item should be part of CONSORT-ROUTINE checklist).

For peer review only - http://bmjopen.bmj.com/site/about/guidelines.xhtml

Participants also had the option to select "Not my expertise" for items if they believed that they did not have the appropriate level of expertise to rate an item. Figure 2 shows a screenshot of an example proposed modification item from the survey:

Items from the CONSORT 2010 statement for which modifications were initially not proposed were also included in the survey so that participants could provide comments or make recommendations for modifications to these items. For all items (proposed modifications and CONSORT 2010 items), we provided participants with the opportunity to give open-ended feedback, using free-text boxes provided at the bottom of each survey page and at the end of the survey. At the end of the survey, participants were asked to provide any additional items that they believed would be important for reporting in trials conducted using cohorts and routinely collected data, but which had not been included in the proposed set of new and modified items.

We launched Round 1 of the survey on February 4, 2019 with 2 weeks to provide responses. Round 2 was launched on March 4, 2019, and Round 3 was launched on April 1, 2019. After each round, the Qualtrics built-in analysis software was used to generate a distribution of scores and to aggregate group results for each item (mean score, maximum and minimum score, standard deviation, variance, percentage ratings of 1-5 ranking for items) and summary statistics were circulated amongst all participants. Individual responses were not fed back. In addition, a bar chart with the ratings and counts for each item was created. Following each round of the survey, the CONSORT-ROUTINE steering committee members reviewed the survey results independently and then met via teleconference to discuss and analyze the results of the survey. During these meetings, decisions were made on how to address comments from

For peer review only - http://bmjopen.bmj.com/site/about/guidelines.xhtml

participants by modifying, adding, or combining items. Notes were also made on comments that reflected a need for explanation in the Explanation & Elaboration companion to the checklist.

We predefined consensus as at least 2/3 of responders rating the importance of an item as 'high' or 'very high'. Items that reached consensus for inclusion were not rated again in the next round. For some items that did not reach consensus, the wording of several items was revised based on participants' suggestions. Items that did not reach consensus were rated again in the next round in their original or revised form. Reports summarizing the Delphi results were circulated after each round including summary statistics such as counts, means, standard deviations and variances for the responses on each item. Reminder emails were sent one week prior to the deadline and extensions were provided if requested for all 3 rounds in order to maximize participation.

Since the Delphi Study was advisory, all items were reviewed and vetted again at the in-person consensus meeting, and comments provided by participants of the Delphi Study were taken into consideration while making decisions to include or exclude items.

Project Phase 4: In-person Consensus Meeting and Development of Checklist Publication

A two-day in-person consensus meeting was held on May 13-14, 2019 in London, United Kingdom. The purpose of the meeting was to discuss the Delphi results, make decisions on items to retain in the final checklist, make any necessary modifications to items, and suggest reporting aspects that should be addressed in the Explanation & Elaboration documentation supporting the checklist. The meeting was attended by 26 members of the CONSORT-ROUTINE Group.

We used approaches similar to those used in previous consensus meetings for other guidelines. Participants were provided with the results of the initial long-list generation and the Delphi study in advance of the meeting. At the meeting, steering committee members first

For peer review only - http://bmjopen.bmj.com/site/about/guidelines.xhtml

presented the background and an update on work done to date, in order to facilitate the discussions. Session chairs then separately presented items from the preliminary checklist, results of the Delphi study, and feedback from stakeholders, after which the group discussed in an open forum. Decisions were made on items to be modified or added based on the following criteria (1) whether they addressed elements unique to trials conducted using cohorts or routinely collected data versus elements applicable to any trial, and (2) whether they reflected information that should be included in a minimum reporting set of items. Notes were taken and the discussions were audio-recorded to ensure that the content was accurately captured.

Following the consensus meeting, refinement of the content and wording of the items was continued through online group discussions with CONSORT-ROUTINE project team members. The initial version of the checklist was pilot-tested by circulating it among stakeholders in order to assess its usability and to identify any challenges which might arise while applying the checklist. Pilot-testing the checklist also provided insight into issues that should be addressed in detail in the Explanation and Elaboration statement.

Project Phase 5: Publication, Dissemination and Implementation

As with several previous CONSORT extensions, it was decided to publish the reporting checklist with a detailed Explanation and Elaboration statement in the same document.[6-9] The Explanation and Elaboration statement is intended to provide an in-depth explanation of the scientific rationale for each recommendation, together with an example of clear reporting for each item.

In addition to publication of the reporting guideline checklist and Explanation & Elaboration material, to attempt to maximize uptake, we will undertake additional dissemination activities, including presentations and workshops at conferences and other venues. We also plan

For peer review only - http://bmjopen.bmj.com/site/about/guidelines.xhtml

to seek endorsement of the guideline by journal editors. Research has shown that formal endorsement and adoption of the CONSORT statement by journals is associated with improved quality of reporting.[2] Studies conducted by members of our team have benchmarked pre-extension reporting completeness in trials conducted in cohorts, registries, EHRs, and administrative databases.[31-33] There were not enough examples of completed cohort-embedded trials for benchmarking reporting.

The final CONSORT-ROUTINE checklist (Kwakkenbos et al., under review) has been published at: [Insert Link – SEE SUPPLEMENTAL MATERIAL]

Patient and public involvement: One of the members of our CONSORT-ROUTINE team, Maureen Sauvé, is a patient organisation leader. She has been involved in working with researchers to establish a cohort of patients living with the rare disease scleroderma, which supports RCTs of trials of online rehabilitation, self-management and psychological intervention programmes.

RESULTS

Stage 2: Scoping review and initial long list of potential items

The scoping review sought methods articles and reports of trials conducted using cohorts, registries, EHRs, or administrative databases.

Cohorts: The database search identified 1,185 publications, of which 1,062 were excluded after title and abstract screening and 37 after full-text review. A total of 86 studies were included in the scoping review, including 15 papers on methodological considerations of using cohorts for conducting RCTs. All trials used the cohort for both identification of patients and outcome ascertainment.

For peer review only - http://bmjopen.bmj.com/site/about/guidelines.xhtml

Registries: The search identified 234 publications, of which 143 received full-text review. A total of 106 publications were eligible, including 95 trial reports or protocols (both identification of patients and outcome ascertainment (n = 27); identification of patients only (n = 28); outcome ascertainment only (n= 40)) and 11 papers on methodological considerations.

EHRs: The search identified 2,085 citations, of which 548 studies were reviewed at the full-text level. A total of 289 eligible publications, including 263 trial protocols or reports (both identification of patients and outcome ascertainment (n= 169); identification of patients only (n = 38); outcome ascertainment only (n = 56)) and 26 articles that described methodological considerations.

Administrative databases: The search identified 663 citations, of which 151 full texts were reviewed. There were a total of 117 trial protocols or reports included (both identification of patients and outcome ascertainment (n = 57); identification of patients only (n= 1); outcome ascertainment only (n = 58)) and 1 paper on methodological considerations.

Delphi Study Results

Of 125 people invited to take part in the Delphi study, 115 people registered via an online survey, and 92 (74%) provided responses on the items in Round 1. Figures 3 and 4 present the types of stakeholder groups that completed Round 1 of the Delphi Study and the type of trials conducted using cohorts or routinely collected databases with which they had familiarity. Participants belonging to more than one category had the option of checking multiple options in the survey.

Round 1: Of the 92 participants who completed the Round 1 survey, out of which 90 provided valid ratings and 2 provided comments but not ratings. Of the 27 items rated in Round 1, 14 reached consensus to be included in discussions at the consensus meeting; the other 13 did

For peer review only - http://bmjopen.bmj.com/site/about/guidelines.xhtml

not reach consensus and were included in Round 2. Based on Round 1 feedback, a total of 11 items were modified for review in Round 2, including 2 items that were combined into one. No items were excluded from the checklist.

Round 2: Of the 92 participants who completed Round 1, 77 (84%) completed the Round 2 survey. Of the 13 items rated, 2 reached consensus for inclusion in consensus meeting discussions, and 11 did not reach consensus in Round 2. Based on Round 2 feedback, 8 items were modified prior to Round 3.

Round 3: Of the 77 people who completed Round 2, 62 (81%) completed Round 3. Of the 11 items in Round, 5 items reached consensus in Round 3. The remaining 6 items did not reach consensus after the 3 rounds.

There were several new items suggested via the Delphi process but not added to the potential item list. The main reasons why some items were suggested but not incorporated were:

(a) The suggestion was encapsulated in CONSORT 2010 items, was already captured by proposed new or modified items, or could be captured by further modifying new or modified items;

(b) The suggestion was not specific to trials conducted using cohorts and routinely collected data and, thus, was recommending a change to the CONSORT 2010 checklist, which was not the task of the CONSORT-ROUTINE group.

Summary results of the 3 rounds can be accessed at: https://osf.io/4zh6f/

In-person Consensus Meeting

Table 1 summarises the CONSORT-ROUTINE group's discussions and advisory decisions for each of the items that was discussed during the in-person meeting. If there were differing opinions on the inclusion or exclusion of items and consensus could not be reached, voting was

For peer review only - http://bmjopen.bmj.com/site/about/guidelines.xhtml

implemented by the session chair, with an 80% threshold for inclusion in the checklist as part of the minimal set of recommended reporting items. The key recommendations that emerged were as follows:

- Proposed modification to CONSORT 2010 items: It was recommended to retain proposed modifications to 7 CONSORT 2010 items. These modifications pertained to differences in mechanisms used to conduct trials using cohorts or routinely collected databases. As in previous CONSORT extensions, some of the recommended changes end with "if applicable" to show that some information which authors are being asked to report might not be relevant or applicable for their particular RCT, or the particular type of data that was used in the RCT.
- Proposed additional items: consensus was reached to include 6 additional items and to add a new subheading, "Cohort or routinely collected database" to the checklist.

A recurrent discussion point was the need to minimise adding new items to the abstract unless they are essential due to word limits imposed by journals. A suggestion was made to expand the explanatory text of the Explanation & Elaboration document for nine unchanged CONSORT 2010 items to clarify additional requirements for reporting aspects of the trial without modifying the item: item 1a (identification as a randomised trial in the title), item 4b (settings and location where the data were collected), item 5 (intervention), item 13b (losses and exclusions after randomisation), item 14a (dates of recruitment/follow-up), item 15 (baseline data), item 20 (limitations), item 21 (generalisability) and item 24 (study protocol). For the abstract, there was an agreement to include an additional item to the abstract for naming the cohort or routinely collected database (item 1c). This item was later merged with item 1b from

For peer review only - http://bmjopen.bmj.com/site/about/guidelines.xhtml

the CONSORT 2010 checklist after discussion with the project team (Table 1). Thus, the final extension checklist included 8 modified items and 5 new items.[23]

CONSORT-ROUTINE Pilot-test

The preliminary version of the checklist was pilot-tested by 17 people who had been previously involved in conducting trials using cohorts and routinely collected data. Based on feedback received from the pilot-test there were minor modifications made to the wording of 2 items for clarity (Item 1b and Item 9) in the final checklist.[23]

DISCUSSION

We have developed a consensus-driven extension to the CONSORT 2010 Statement for RCTs conducted using cohorts and routinely collected data (Kwakkenbos et al., under review). CONSORT-ROUTINE contains minimum reporting requirements with appropriate flexibility as described in the Explanation & Elaboration part of our checklist document. This article described how we reached the final checklist and Explanation & Elaboration text and provides information on the decision-making process. We anticipate this paper will help others who may learn from our experiences and may apply this to the development of future guidelines or extensions.

There were several important strengths to our approach. A consensus-driven Delphi methodology, which is recommended when developing health care reporting guidelines by the EQUATOR network, was used to develop the extension.[23] We engaged with key stakeholders in trials research and potential end-users of the resultant CONSORT-ROUTINE reporting guideline throughout the development process. The process involved participants from a wide range of scientific disciplines and with diverse experience in conducting trials using different cohorts and routinely collected databases. As with other CONSORT-related guidelines, the inclusion of CONSORT Group members (IB, DM, PR) was intended to ensure consistency in the use of

For peer review only - http://bmjopen.bmj.com/site/about/guidelines.xhtml

recommended methods in the development, dissemination, and implementation of the extension. We recorded high response rates of 74% (92 respondents), 84% (77 respondents) and 81% (62 respondents) in Delphi rounds 1, 2, and 3, respectively. In addition, the number of registered participants and responders is larger than in most Delphi surveys used to develop health care reporting guidelines.[8,34,35] Finally, we achieved a high degree of consensus that was consistent across Delphi survey rounds for the majority of the items.

There are also limitations to consider. One is that most participants were academic researchers with primary roles in trials research, and, despite our broad engagement efforts, the number of participants from some stakeholder groups was small. One patient was included as a member of the reporting guideline development team, but no patients participated in the Delphi exercise. It is possible that perceptions about the importance of items might have differed across different stakeholder groups which might have favoured the inclusion or exclusion of certain items. Nonetheless, our project group included people from diverse backgrounds with expertise in using different types of data sources, who oversaw the development process to ensure that the checklist was equally applicable to, and representative of, all 4 types of data sources. A second is that our scoping review was not designed to capture each and every trial conducted using routinely collected data. This was in part because of the lack of accepted specific Medical Subject Headings terms to identify these studies, or any research using routinely collected data, and the limited number of completed trials and methodological articles on these trial designs. For our purposes it was not necessary to capture all trials that had been conducted using cohorts or routinely collected data, and we believe that we were able to capture a significant number of important trial reports and methodology papers that served as a basis for the development of our extension. A third is that the CONSORT-ROUTINE group predominantly consisted of members

For peer review only - http://bmjopen.bmj.com/site/about/guidelines.xhtml

from high-income countries, which might have led to decreased applicability of the checklist for trials conducted in other settings. Finally, as with all reporting guidelines, ours will require re-evaluation and revisions over time to ensure that it is kept up to date with evolving research and knowledge on these trail designs.

CONCLUSION

CONSORT-ROUTINE has now been developed and can be used to support comprehensive reporting of RCTs conducted using cohorts or routinely collected data. The extension statement contains minimum requirements of reporting that we encourage researchers to report. A baseline assessment of the completeness and reporting of these trial designs is being conducted, and the impact of the extension will be assessed in the coming years. While we anticipate that CONSORT-ROUTINE may need to be updated with the evolution of research methods, we hope the guideline will improve the reporting of RCTs conducted using cohorts and routinely collected data, enhance their interpretability and credibility of their results, improve their reproducibility, indirectly facilitate their robust design and conduct and lead to improved patient care.

For peer review only

For peer review only - http://bmjopen.bmj.com/site/about/guidelines.xhtml

Contributors: MI, LK, OF, LGH, MZ, CR, SML, DM, MS, CG, EJ and BDT were involved in initial phases of study conception, design of the search strategy and development of conceptual frameworks. SM, KAMcC, DBR, EIB, LT, MKC, DE, HMV, IB, PR, JN, RU, MS, JF and DT provided regular feedback on each of these steps. MI wrote the first draft with LK and BDT. All authors made provided critical revisions to the development of this manuscript and approved the final version.

Funding: The development of CONSORT-ROUTINE was supported by the Canadian Institutes of Health Research (CIHR; PJT156172; PCS-161863), and the United Kingdom National Institute of Health Research (NIHR) Clinical Trials Unit Support Funding - Supporting efficient / innovative delivery of NIHR research (PI Juszczak, Co-PI Gale). DBR was supported by a Vanier CIHR Graduate Scholarship, SML was supported by a Wellcome Senior Clinical Fellowship in Science (205039/Z/16/Z), EIB was supported by a New Investigator Award from Canadian Institutes of Health Research (CIHR), the Canadian Association of Gastroenterology and Crohn’s and Colitis Canada, and the Career Enhancement Program of the Canadian Child Health Clinician Scientist Program, RU was supported by the Canada Research Chairs Program (Award #231397), CG was supported by the United Kingdom Medical Research Council through a Clinician Scientist Fellowship, and BDT was supported by a Tier 1 Canada Research Chair, all outside of the present work.

Disclaimer: The views expressed are those of the authors and not necessarily those of the NHS, the NIHR or the Department of Health and Social Care.

For peer review only - http://bmjopen.bmj.com/site/about/guidelines.xhtml

Competing interests: The authors have read and understood the BMJ policy on declaration of interests and declare that they have no competing interests.

Patient consent: Not required.

Ethics approval: This study does not require ethics approval.

Data Sharing: All data relevant to the study are included in the article or uploaded as supplementary information

For peer review only

For peer review only - http://bmjopen.bmj.com/site/about/guidelines.xhtml

FIGURE LEGENDS

Figure 1: Development process of the CONSORT Extension for Trials Conducted Using Cohorts and Routinely Collected Data (CONSORT-ROUTINE)

Figure 2: Example of a round 1 Delphi survey item as presented in the online survey.

Figure 3: Professional roles reported by participants who completed Round 1 of the CONSORT-ROUTINE Delphi Study (%). Participants could report more than one role.

Figure 4: Participants of Round 1 of the CONSORT-ROUTINE Delphi Study by type of cohort or routinely collected database with which they had familiarity (%). Participants could report more than one.

For peer review only

For peer review only - http://bmjopen.bmj.com/site/about/guidelines.xhtml

REFERENCES

1. Altman DG. Transparent reporting of trials is essential. *Am J Gastroenterol*. 2013;108(8):1231-1235.

2. Turner L, Shamseer L, Altman DG, et al. Consolidated standards of reporting trials (CONSORT) and the completeness of reporting of randomised controlled trials (RCTs) published in medical journals. *Cochrane Database Syst Rev*. 2012;11:MR000030.

3. Cobo E, Cortés J, Ribera JM, et al. Effect of using reporting guidelines during peer review on quality of final manuscripts submitted to a biomedical journal: masked randomised trial. *BMJ*. 2011;343:d6783.

4. Moher D, Jones A, Lepage L; CONSORT Group (Consolidated Standards for Reporting of Trials). Use of the CONSORT statement and quality of reports of randomized trials: a comparative before-and-after evaluation. *JAMA*. 2001;285(15):1992-1995.

5. Schulz KF, Altman DG, Moher D; CONSORT Group. CONSORT 2010 statement: updated guidelines for reporting parallel group randomised trials. *BMJ*. 2010;340:c332.

6. Juszczak E, Altman DG, Hopewell S, Schulz K. Reporting of Multi-Arm Parallel-Group Randomized Trials: Extension of the CONSORT 2010 Statement. *JAMA*. 2019;321(16):1610-1620.

7. Campbell MK, Elbourne DR, Altman DG; CONSORT group. CONSORT statement: extension to cluster randomised trials. *BMJ*. 2004;328(7441):702-708.

8. Eldridge SM, Chan CL, Campbell MJ, et al. CONSORT 2010 statement: extension to randomised pilot and feasibility trials. *BMJ*. 2016;355:i5239.

9. Zwarenstein M, Treweek S, Gagnier JJ, et al. Improving the reporting of pragmatic trials: an extension of the CONSORT statement. *BMJ*. 2008;337:a2390.

For peer review only - http://bmjopen.bmj.com/site/about/guidelines.xhtml

10. Anderson GL, Burns CJ, Larsen J, Shaw PA. Use of administrative data to increase the practicality of clinical trials: Insights from the Women's Health Initiative. *Clin Trials*. 2016;13(5):519-526.

11. James S, Fröbert O, Lagerqvist B. Cardiovascular registries: a novel platform for randomised clinical trials. *Heart*. 2012;98(18):1329-1331.

12. James S, Rao SV, Granger CB. Registry-based randomized clinical trials--a new clinical trial paradigm. *Nat Rev Cardiol*. 2015;12(5):312-316.

13. Relton C, Torgerson D, O'Cathain A, Nicholl J. Rethinking pragmatic randomised controlled trials: introducing the "cohort multiple randomised controlled trial" design. *BMJ*. 2010;340:c1066.

14. van Staa TP, Dyson L, McCann G, et al. The opportunities and challenges of pragmatic point-of-care randomised trials using routinely collected electronic records: evaluations of two exemplar trials. *Health Technol Assess*. 2014;18(43):1-146.

15. Morrato EH, Elias M, Gericke CA. Using population-based routine data for evidence-based health policy decisions: lessons from three examples of setting and evaluating national health policy in Australia, the UK and the USA. *J Public Health*. 2007;29(4):463-471.

16. Moskowitz A, Chen KP, Cooper AZ, Chahin A, Ghassemi MM, Celi LA. Management of Atrial Fibrillation with Rapid Ventricular Response in the Intensive Care Unit: A Secondary Analysis of Electronic Health Record Data. *Shock*. 2017;48(4):436-440.

17. Hemkens LG, Saccilotto R, Reyes SL, et al. Personalized prescription feedback to reduce antibiotic overuse in primary care: rationale and design of a nationwide pragmatic randomized trial. *BMC Infect Dis*. 2016;16:421.

For peer review only

For peer review only - http://bmjopen.bmj.com/site/about/guidelines.xhtml

18. Mc Cord KA, Ewald H, Ladanie A, et al. Current use and costs of electronic health records for clinical trial research: a descriptive study. *CMAJ Open*. 2019;7(1):E23-E32.

19. Wachtell K, Lagerqvist B, Olivecrona GK, James SK, Fröbert O. Novel Trial Designs: Lessons Learned from Thrombus Aspiration During ST-Segment Elevation Myocardial Infarction in Scandinavia (TASTE) Trial. *Curr Cardiol Rep*. 2016;18(1):11.

20. Benchimol EI, Smeeth L, Guttmann A, et al. The REporting of studies Conducted using Observational Routinely-collected health Data (RECORD) statement. *PLoS Med*. 2015;12(10):e1001885.

21. Hemkens LG, Benchimol EI, Langan SM, et al. The reporting of studies using routinely collected health data was often insufficient. *J Clin Epidemiol*. 2016;79:104-111.

22. Kwakkenbos L, Juszczak E, Hemkens LG, et al. Protocol for the development of a CONSORT extension for RCTs using cohorts and routinely collected health data. *Res Integr Peer Rev*. 2018;3:9.

23. Moher D, Schulz KF, Simera I, Altman DG. Guidance for developers of health research reporting guidelines. *PLoS Med*. 2010;7(2):e1000217.

24. Equator Network. http://www.equator-network.org/library/reporting-guidelines-under-development/. Accessed November 2, 2020.

25. Relton, C, Burbach, M, Collett, C, at al. The ethics of 'Trials within Cohorts'(TwiCs): 2nd international symposium. *Trials*. 2017; 18(2):1-10.

26. von Elm E, Altman DG, Egger M, et al. The Strengthening the Reporting of Observational Studies in Epidemiology (STROBE) Statement: guidelines for reporting observational studies. *BMJ*. 2007;335(7624):806-808.

For peer review only

For peer review only - http://bmjopen.bmj.com/site/about/guidelines.xhtml

27. Kwakkenbos L, Imran M, Mc Cord KA, et al. Protocol for a scoping review to support development of a CONSORT extension for randomised controlled trials using cohorts and routinely collected health data. *BMJ Open*. 2018;8(8):e025266.

28. Trevelyan, EG, & Robinson, N. Delphi methodology in health research: how to do it?. *Eur J Integr Med.* 2015; 7(4): 423-428.

29. Okoli C, Pawlowski SD. The Delphi method as a research tool: an example, design considerations and applications. *Inform Manage*. 2004;42:15-29.

30. Hasson F, Keeney S, McKenna H. Research guidelines for the Delphi survey technique. *J Adv Nurs*. 2000;32:1008-15.

31. McCall SJ, Imran M, Hemkens LG, et al. A review of randomised controlled trials conducted using electronic health records with an assessment of completenessand transparency of reporting: study protocol. 2019. Available from: https://osf.io/p6wa4/. Accessed November 2, 2020.

32. Mc Cord KA., Imran M, McCall SJ, et al. Systematic analysis of the reporting transparency and completeness of randomized controlled trials using registries: a protocol. 2019. Available from: https://osf.io/x75gf/. Accessed November 2, 2020.

33. Imran M, Mc Cord KA, McCall SJ, et al. Protocol for a Completeness and Transparency of Reporting Study of Randomised Controlled Trials Conducted using Administrative Databases. 2019. Available from: https://osf.io/dp23x/. Accessed November 2, 2020.

34. Agha RA, Fowler AJ, Rajmohan S, Barai I, Orgill DP; PROCESS Group. Preferred reporting of case series in surgery; the PROCESS guidelines. *Int J Surg*. 2016;36(Pt A):319-323.

35. Vohra S, Shamseer L, Sampson M, et al. CONSORT extension for reporting N-of-1 trials (CENT) 2015 Statement. *BMJ*. 2015;350:h1738.

For peer review only - http://bmjopen.bmj.com/site/about/guidelines.xhtml

Table 1. Consensus meeting discussions and advisory decisions for the checklist items.

Section/Topic	CONSORT 2010 Item	CON-SORT Ext. Item		CONSORT 2010 item	Suggested modified or additional extension items	Consensus Status (Delphi)	Summary of the discussion, decisions and suggestions made during the CONSORT-ROUTINE in-person consensus meeting	Final checklist item to be included in CONSORT-ROUTINE
**Title and abstract**								
	1a	1a		Identification as a randomised trial in the title	Identification as a randomised trial in the title, including that it was a trial conducted using a cohort or routinely collected source of data (Modified)	Not reached	Discussed the need for a modification to the original item. It was noted that multiple databases or types of databases could be used to conduct a trial and stating all would not be feasible as journals might have title length restrictions.<br><br><u>Decision</u>: Do not include the modification and retain the CONSORT 2010 item; expand the E&E text for clarification	*Identification as a randomised trial in the title*
	1b	1b		Structured summary of trial design, methods, results, and conclusions (for specific guidance see CONSORT for abstracts)			No suggested modification.<br><br><u>Decision</u>: Retain the CONSORT 2010 item.<br><br><u>Note:</u> Additional Item 1c was later merged with this item (see below)	*Structured summary of trial design, methods, results, and conclusions (for specific guidance see CONSORT for abstracts). Specify that a cohort or routinely collected data were used to conduct the trial and, if applicable, provide the name of the cohort or routinely collected database(s)*
					The source(s) of data used to conduct the trial should be specified in the abstract (Additional)	Reached for inclusion	Noted the importance of stating the cohort or routinely collected database(s) used to conduct the trial in the abstract, if not in the title.<br><br><u>Decision</u>: Include the suggested new item with revisions.<br><br><u>Note:</u> The item was later merged with Item 1b from CONSORT 2010	
					If linkage between multiple sources of data was conducted for the study, this should be clearly stated in the abstract (Additional)	Not reached	Mixed views on the necessity of reporting the suggested new item in the abstract. Agreed that linkage is important to report in the body of the paper, but not necessarily the abstract.<br><br><u>Decision</u>: Do not include the suggested new item.	
					The proportion of participants offered and the proportion that accepted the intervention should be reported (for trials conducted using the cohort multiple RCT design) (Additional)	Reached for inclusion	Mixed views on the necessity of reporting the suggested new item in the abstract. Agreement that the information is important to report but not essential for the abstract due to word count restrictions. In addition, this applies to one trial design used in cohorts, but not all cohort trials and not trials using other types of data. The item was merged with CONSORT 2010 item 13a (14a in the final extension checklist) pertaining to participant flow.<br><br><u>Decision</u>: Do not include the suggested new item in the abstract but include in item 14a in the final extension checklist.	
**Introduction**								
Background and objectives	2a	2a		Scientific background and explanation of rationale			Discussed the importance of reporting the rationale for conducting the trial using a cohort or routinely collected database but decided against modifying original CONSORT 2010 item.<br><br><u>Decision</u>: Retain the CONSORT 2010 item.	*Scientific background and explanation of rationale*
	2b	2b		Specific objectives or hypotheses			No suggested modification.<br><br><u>Decision</u>: Retain the CONSORT 2010 item.	*Specific objectives or hypotheses*
**Methods**								
Trial design	3a	3a		Description of trial design (such as parallel, factorial) including allocation ratio	Description of trial design (such as parallel, factorial) including allocation ratio, the source(s) of data used to conduct the trial (such as cohort, registry) and how the data are used within the trial (such as		Noted that key elements of the study design and cohort or database(s) used for the trial should be stated early in the methods section, as well as the extent to which the database was used in the trial.	*Description of trial design (such as parallel, factorial) including allocation ratio, that a cohort or routinely collected database(s) used to conduct the trial (such as electronic health record, registry) and how the data*

For peer review only

					identification of eligible trial participants, trial outcomes) (Modified)		Decision: Include the modified item.	*were used within the trial (such as identification of eligible trial participants, trial outcomes)*
	3b	3b		Important changes to methods after trial commencement (such as eligibility criteria), with reasons			No suggested modification.<br><br>Decision: Retain the CONSORT 2010 item.	*Important changes to methods after trial commencement (such as eligibility criteria), with reasons*
Cohort or routinely collected database (Additional header)		ROUTINE-1			Description of the source(s) of data used to conduct the trial, including the setting, locations, relevant dates, periods of recruitment, follow-up, and data collection (Additional)	Reached for inclusion	Agreed on the importance of reporting the item.<br><br>Decision: Include the suggested new item.	*Name, if applicable, and description of the cohort or routinely collected database(s) used to conduct the trial, including information on the setting (such as primary care), locations, and dates, (such as periods of recruitment, follow-up, and data collection)*
					Describe indicators of the quality of the source(s) of data used to conduct the trial including what types of quality checks have been performed and the entity responsible for the data (Additional)	Reached for inclusion	Mixed views on the necessity of the suggested new item. There were concerns that "quality" is vague and the term "accuracy and completeness" may better clarify the intent of the item. It was acknowledged that the accuracy and completeness of the cohort or database is important to report while (i) selecting participants and (ii) ascertaining outcomes.<br><br>Decision: Do not include the suggested new item as a stand-alone item. The item was merged with extension items 5a and 7b (pertaining to participant selection and outcome ascertainment) in the finalised checklist.	
					Describe modifications to the data collected in the source(s) of data used to conduct the trial, such as adding data items, if applicable (Additional)	Reached for inclusion	Agreed that the suggested item is not necessarily unique to trials conducted using cohorts and routinely collected data.<br><br>Decision: Do not include the suggested new item; expand the E&E text for clarification.	
					Describe additional sources of data used to conduct the trial, if any (Additional)	Reached for inclusion	Mixed views on the necessity of the suggested new item as it is not unique to trials conducted using cohorts and routinely collected data.<br><br>Decision: Do not include the suggested new item; expand the E&E text for clarification.	
		ROUTINE-2			Give the eligibility criteria, the sources and methods of selection of participants, and methods of follow-up (for trials conducted using cohorts or registries) (Additional)	Reached for inclusion	Discussed the importance of reporting the eligibility criteria for inclusion in the cohort or routinely collected database(s), but there was concern that elements related to follow-up are not specific to trials conducted using cohorts and routinely collected data.<br><br>Decision: Include the suggested new item with revisions; expand the E&E text for clarification of other aspects.	*Eligibility criteria for participants in the cohort or routinely collected database(s)*
		ROUTINE-3			Detail any use of record linkage across sources of data, the methods of linkage and methods of quality evaluation, if applicable (Additional)	Reached for inclusion	Suggestion to integrate wording from RECORD checklist for clarity.<br><br>Decision: Include the suggested new item adapted from RECORD.	*State whether the study included person-level, institutional-level, or other data linkage across two or more databases and, if so, linkage techniques and methods used to evaluate completeness and accuracy of linkage*
					Describe if (and how) participants were informed about the potential use of their data in randomised trials (Additional)	Not reached	Mixed views on the necessity of the item as some believed that ethics considerations are beyond the scope of CONSORT, and ethics does not appear in CONSORT 2010. The group agreed to include the item as consent is an important issue with unique aspects in these trials, but that this should be presented as part of trial participants section.<br><br>Decision: Include the suggested new item with revisions and move to section "Trial participants" as Item 5c.	
Trial Participants (Modified header)	4a	4a		Eligibility criteria for participants	Eligibility criteria for trial participants (Modified)		Agreed to merge with suggested new item (see next row).<br><br>Decision: Merge with suggested new item, "Provide details of how eligible clusters/participants were identified from the source(s) of data used to conduct the trial".	*Eligibility criteria for trial participants, including information on how to access the list of codes and algorithms used to identify eligible participants, information on accuracy and completeness of data used to ascertain eligibility, and methods used to validate accuracy and completeness (e.g., monitoring, adjudication), if applicable*
					Provide details of how eligible clusters/participants were identified from the source(s) of data used to conduct the trial (Additional)	Reached for inclusion	Suggested merging with CONSORT 2010 Item 4a (5a in final checklist) and address accuracy and completeness of data.	

For peer review only

							Decision: Merge the suggested new item with CONOSRT 2010 item 4a (5a in the final checklist).	
	4b	4b		Settings and locations where the data were collected	Settings and locations where the trial data were collected (Modified)	Reached for inclusion	The word "trial" was dropped as the header "Trial participants" clarifies the intent of the item.<br><br>Decision: Retain the CONSORT 2010 item; expand the E&E text for clarification.	*Settings and locations where the data were collected*
		ROUTINE-4			Details of information provided to participants from the source(s) of data who are selected for recruitment or inclusion in the trial, including any differences in information provided across trial arms (Additional)	Not reached	Extended discussions on the importance of the item as it might only be applicable to cmRCTs. Agreement to formulate as a general item on consent as Item 5c.<br><br>Decision: Do not include the suggested new item. The consent item was simplified and moved to this section. Expand the E&E text for clarification of consent issues.	*Describe whether and how consent was obtained*
Interventions	5	5		The interventions for each group with sufficient details to allow replication, including how and when they were actually administered			No suggested modification.<br><br>Decision: Retain the CONSORT 2010 item; expand the E&E text for clarification.	*The interventions for each group with sufficient details to allow replication, including how and when they were actually administered*
					Describe how the source(s) of data was used to implement the intervention, if applicable (e.g., for trials conducted using electronic health records) (Additional)	Reached for inclusion	Debated the necessity of the new item as it is only applicable to trials conducted using electronic health records that may be used as intervention tools.<br><br>Decision: Do not include the suggested new item; expand the E&E text for clarification.	
Outcomes	6a	6a		Completely defined pre-specified primary and secondary outcome measures, including how and when they were assessed			Suggestion to merge with proposed new item, "Provide source(s) of data for each outcome" (see below).<br><br>Decision: Item merged with suggested new item and included in the final checklist.	*Completely defined pre-specified primary and secondary outcome measures, including how and when they were ascertained and the cohort or routinely collected database(s) used to ascertain each outcome*
					Provide source(s) of data for each outcome (Additional)	Reached for inclusion	Suggestion to merge with CONSORT 2010 item 6a.<br><br>Decision: Item merged with CONSORT 2010 item 6a (7a in the final checklist).	
		ROUTINE-5			Provide a list of codes and algorithms used to define (and/or derive) the outcomes as supplementary information, including validation, if applicable (Additional)	Not reached	Acknowledged the importance of reporting the list of codes and algorithms for ascertaining outcomes along with the accuracy and completeness of data and validation.<br><br>Decision: Include the suggested new item with revisions.	*Information on how to access the list of codes and algorithms used to define or derive the outcomes from the cohort or routinely collected database(s) used to conduct the trial, information on accuracy and completeness of outcome variables, and methods used to validate accuracy and completeness (e.g., monitoring, adjudication), if applicable*
					Detail any adjudication or external validation of data items from the source(s) of data used to conduct the trial, if applicable (Additional)	Reached for inclusion	Acknowledged the importance of reporting the item. There was agreement that validation should be reported while selecting participants and ascertaining outcomes and included as part of items 5a and 7b of extension checklist.<br><br>Decision: Address elements of proposed item as part of items 5a and 7b in the final checklist.	
	6b	6b		Any changes to trial outcomes after the trial commenced, with reasons			No suggested modification.<br><br>Decision: Retain the CONSORT 2010 item.	*Any changes to trial outcomes after the trial commenced, with reasons*
Sample size	7a	7a		How sample size was determined			No suggested modification.<br><br>Decision: Retain the CONSORT 2010 item.	*How sample size was determined*
	7b	7b		When applicable, explanation of any interim analyses and stopping guidelines			No suggested modification.<br><br>Decision: Retain the CONSORT 2010 item.	*When applicable, explanation of any interim analyses and stopping guidelines*
Randomisation:								
Sequence generation	8a	8a		Method used to generate the random allocation sequence			No suggested modification.<br><br>Decision: Retain the CONSORT 2010 item; expand the E&E text for clarification.	*Method used to generate the random allocation sequence*
	8b	8b		Type of randomisation; details of any restriction (such as blocking and block size)			No suggested modification.<br><br>Decision: Retain the CONSORT 2010 item.	*Type of randomisation; details of any restriction (such as blocking and block size)*
Allocation concealment	9	9		Mechanism used to implement the random allocation sequence (such as sequentially numbered containers),	Mechanism used to implement the random allocation sequence, describing any steps	Reached for inclusion	Discussion to clarify wording of the item.	*Mechanism used to implement the random allocation sequence (such as embedding an automated randomiser*

For peer review only - http://bmjopen.bmj.com/site/about/guidelines.xhtml

mechanism				describing any steps taken to conceal the sequence until interventions were assigned	taken to conceal the sequence until interventions were assigned, such as using automated random sequence generation concealed within source(s) of data (Modified)		Decision: Include the modified item with revisions.	*within the cohort or routinely collected database(s)), describing any steps taken to conceal the sequence until interventions were assigned*
Implementation	10	10		Who generated the random allocation sequence, who enrolled participants, and who assigned participants to interventions			No suggested modification.<br><br>Decision: Retain the CONSORT 2010 item.	*Who generated the random allocation sequence, who enrolled participants, and who assigned participants to interventions*
Blinding	11a	11a		If done, who was blinded after assignment to interventions (for example, participants, care providers, those assessing outcomes) and how			No suggested modification.<br><br>Decision: Retain the CONSORT 2010 item.	*If done, who was blinded after assignment to interventions (for example, participants, care providers, those assessing outcomes) and how*
	11b	11b		If relevant, description of the similarity of interventions			No suggested modification.<br><br>Decision: Retain the CONSORT 2010 item.	*If relevant, description of the similarity of interventions*
Statistical methods	12a	12a		Statistical methods used to compare groups for primary and secondary outcomes			No suggested modification.<br><br>Decision: Retain the CONSORT 2010 item.	*Statistical methods used to compare groups for primary and secondary outcomes*
	12b	12b		Methods for additional analyses, such as subgroup analyses and adjusted analyses			No suggested modification.<br><br>Decision: Retain the CONSORT 2010 item.	*Methods for additional analyses, such as subgroup analyses and adjusted analyses*
**Results**								
Participant flow (a diagram is strongly recommended)	13a	13a		For each group, the numbers of participants who were randomly assigned, received intended treatment, and were analysed for the primary outcome	Describe in detail the numbers of clusters/participants in the source(s) of data used to conduct the trial, number screened for eligibility, randomly assigned, offered and accepted interventions (e.g., cohort multiple RCTs), received intended treatment, and analysed for the primary outcome (Modified)	Reached for inclusion	Suggestion to form a committee to draft example flow diagram and oversee the E&E.<br><br>Decision: Include the modified item; committee to oversee the E&E development.	*For each group, the number of participants in the cohort or routinely collected database(s) used to conduct the trial and the numbers screened for eligibility, randomly assigned, offered and accepted interventions (e.g., cohort multiple RCTs), received intended treatment, and analysed for the primary outcome*
					Describe any linkage of multiple sources of data, including the number of clusters/participants successfully linked (Additional)	Reached for inclusion	Debated the necessity of the item as a stand-alone item as linkage was addressed in item 4c. Suggested to include the number of clusters/participants successfully linked as part of the flow diagram.<br><br>Decision: Do not include the suggested new item; expand the E&E text for clarification.	
	13b	13b		For each group, losses and exclusions after randomisation, together with reasons			No suggested modification. Discussed that the item should be tied to data accuracy and completeness, and linkage.<br><br>Decision: Retain the CONSORT 2010 item; expand the E&E text for clarification.	*For each group, losses and exclusions after randomisation, together with reasons*
Recruitment	14a	14a		Dates defining the periods of recruitment and follow-up			No suggested modification.<br><br>Decision: Retain the CONSORT 2010 item.	*Dates defining the periods of recruitment and follow-up*
	14b	14b		Why the trial ended or was stopped			No suggested modification.<br><br>Decision: Retain the CONSORT 2010 item.	*Why the trial ended or was stopped*
Baseline data	15	15		A table showing baseline demographic and clinical characteristics for each group			No suggested modification.<br><br>Decision: Retain the CONSORT 2010 item.	*A table showing baseline demographic and clinical characteristics for each group*
					A table showing baseline demographic and clinical characteristics for eligible participants who participated in the trial and those who did not (Additional)	Reached for inclusion	Agreement to not include the suggested new item as a stand-alone item. The information should be reported if possible, but not necessary, and implications should be addressed as part of "Generalisability" (Item 21).<br><br>Decision: Do not include the suggested new item.	
Numbers analysed	16	16		For each group, number of participants (denominator) included in each analysis and whether the analysis was by original assigned groups			No suggested modification.<br><br>Decision: Retain the CONSORT 2010 item.	*For each group, number of participants (denominator) included in each analysis and whether the analysis was by original assigned groups*
Outcomes and estimation	17a	17a		For each primary and secondary outcome, results for each group, and the estimated effect size and its precision (such as 95% confidence interval)			No suggested modification.<br><br>Decision: Retain the CONSORT 2010 item.	*For each primary and secondary outcome, results for each group, and the estimated effect size and its precision (such as 95% confidence interval)*
	17b	17b		For binary outcomes, presentation of both absolute and relative effect sizes is recommended			No suggested modification.<br><br>Decision: Retain the CONSORT 2010 item.	*For binary outcomes, presentation of both absolute and relative effect sizes is recommended*

For peer review only - http://bmjopen.bmj.com/site/about/guidelines.xhtml

Ancillary analyses	18	18		Results of any other analyses performed, including subgroup analyses and adjusted analyses, distinguishing pre-specified from exploratory			No suggested modification.<br><br>Decision: Retain the CONSORT 2010 item.	*Results of any other analyses performed, including subgroup analyses and adjusted analyses, distinguishing pre-specified from exploratory*
					If outcomes for eligible patients in the existing source(s) of data who were not included in the trial are known, they should be reported (Additional)	Not reached	Agreement to not include the suggested new item as a stand-alone item. The information should be reported if possible, but not necessary, and implications should be addressed as part of “Generalisability”.<br><br>Decision: Do not include the suggested new item; expand the E&E text for clarification in the “Generalisability” section.	
Harms	19	19		All important harms or unintended effects in each group (for specific guidance see CONSORT for harms)			No suggested modification.<br><br>Decision: Retain the CONSORT 2010 item.	*All important harms or unintended effects in each group (for specific guidance see CONSORT for harms)*
**Discussion**								
Limitations	20	20		Trial limitations, addressing sources of potential bias, imprecision, and, if relevant, multiplicity of analyses			No suggested modification.<br><br>Decision: Retain the CONSORT 2010 item.	*Trial limitations, addressing sources of potential bias, imprecision, and, if relevant, multiplicity of analyses*
					Discuss the implications of using data that were not created or collected to answer the specific research question(s) (Additional)	Reached for inclusion	Discussed that using routinely collected data is not necessarily a limitation, and the content of this item should be addressed in the “Interpretation” section.<br><br>Decision: Do not include the suggested new item; merge with CONSORT 2010 item 22 (23 in the final checklist); expand the E&E text for clarification in the “Generalisability” section.	
Generalisability	21	21		Generalisability (external validity, applicability) of the trial findings			No suggested modification. Agreement to elaborate on the representativeness of the cohort or routinely collected database(s) used for the trial, including issues related to characteristics of eligible cohort or database participants who do not agree to participate in trial.<br><br>Decision: Retain the CONSORT 2010 item; expand the E&E text for clarification.	*Generalisability (external validity, applicability) of the trial findings*
Interpretation	22	22		Interpretation consistent with results, balancing benefits and harms, and considering other relevant evidence			Item merged with the proposed new item “Discuss the implications of using data that were not created or collected to answer the specific research question(s)”.<br><br>Decision: Include the modified item.	*Interpretation consistent with results, balancing benefits and harms, and considering other relevant evidence, including the implications of using data that were not collected to answer the trial research questions*
**Other information**								
Registration	23	23		Registration number and name of trial registry			No suggested modification.<br><br>Decision: Retain the CONSORT 2010 item.	*Registration number and name of trial registry*
Protocol	24	24		Where the full trial protocol can be accessed, if available			No suggested modification.<br><br>Decision: Retain the CONSORT 2010 item; expand the E&E text for clarification.	*Where the full trial protocol can be accessed, if available*
Funding	25	25		Sources of funding and other support (such as supply of drugs), role of funders	Sources of funding and other support for the trial and the existing source(s) of data, role of funders (Modified)	Reached for inclusion	Suggested minor revision to the item.<br><br>Decision: Include the modified item with revision.	*Sources of funding and other support for both the trial and the cohort or routinely collected database(s), role of funders*

For peer review only

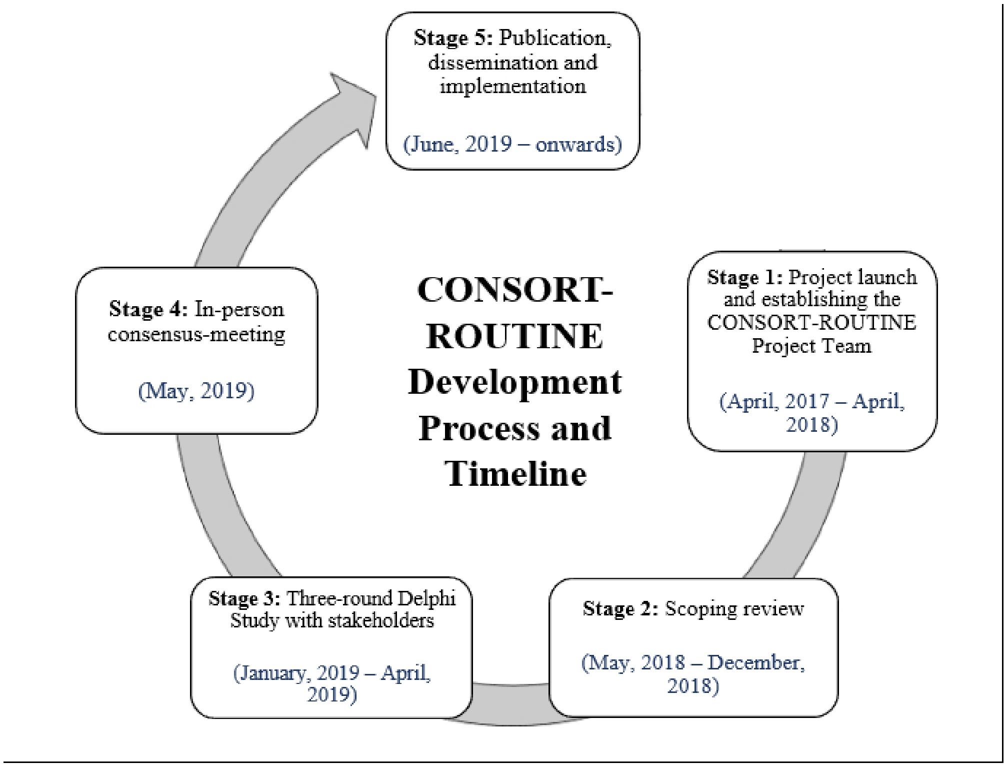

Figure 1: Development process of the CONSORT Extension for Trials Conducted Using Cohorts and Routinely Collected Data (CONSORT-ROUTINE)

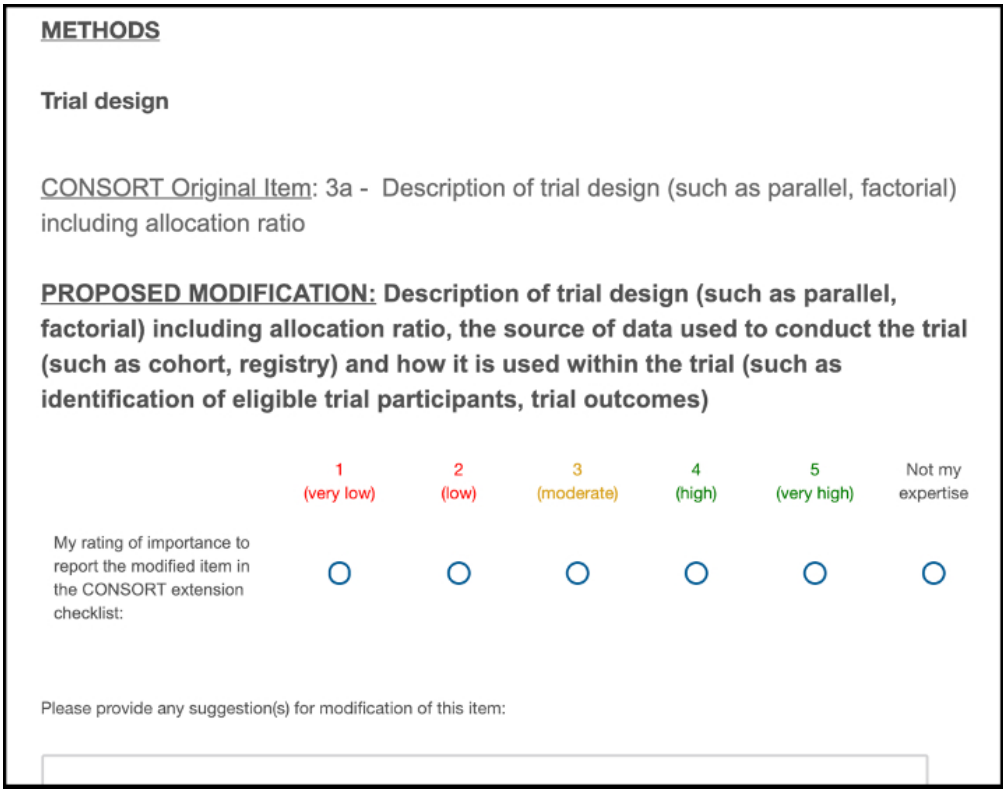

METHODS

Trial design

CONSORT Original Item: 3a - Description of trial design (such as parallel, factorial) including allocation ratio

PROPOSED MODIFICATION: Description of trial design (such as parallel, factorial) including allocation ratio, the source of data used to conduct the trial (such as cohort, registry) and how it is used within the trial (such as identification of eligible trial participants, trial outcomes)

	1 (very low)	2 (low)	3 (moderate)	4 (high)	5 (very high)	Not my expertise
My rating of importance to report the modified item in the CONSORT extension checklist:	○	○	○	○	○	○

Please provide any suggestion(s) for modification of this item:

Figure 2

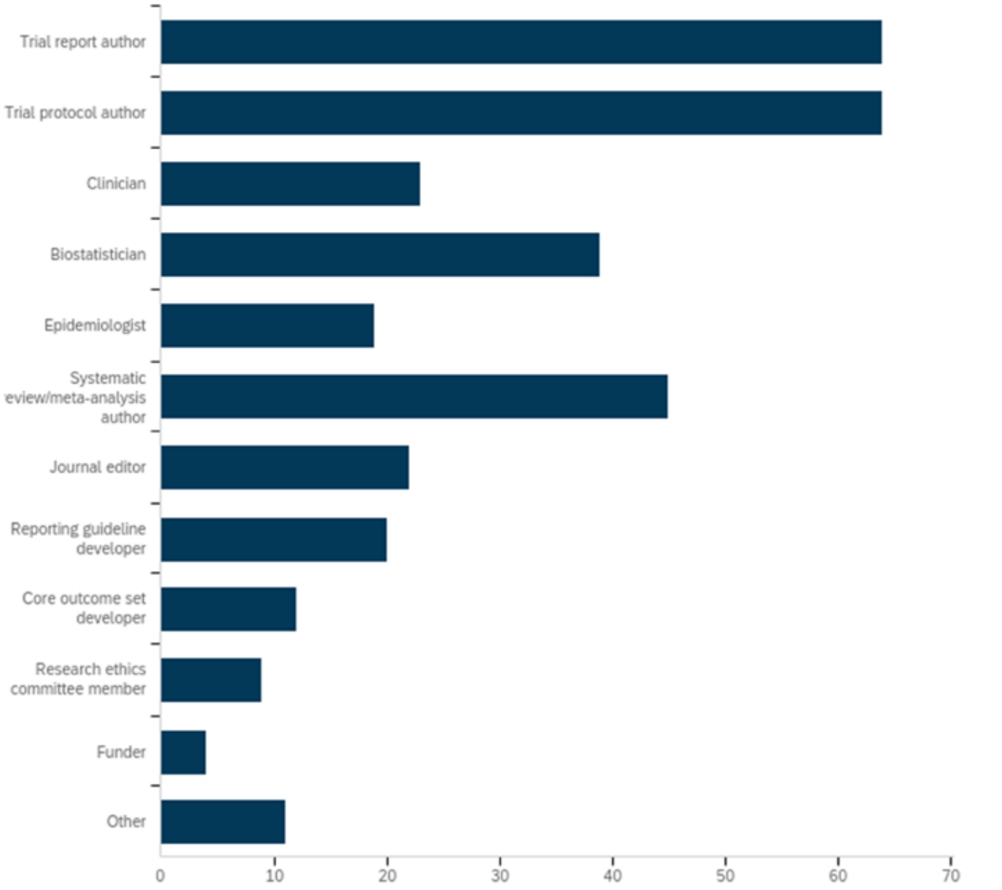
Trial report author
Trial protocol author
Clinician
Biostatistician
Epidemiologist
Systematic
eview/meta-analysis
author
Journal editor
Reporting guideline
developer
Core outcome set
developer
Research ethics
committee member
Funder
Other
0
10
20
30
40
50
60
70

Figure 3

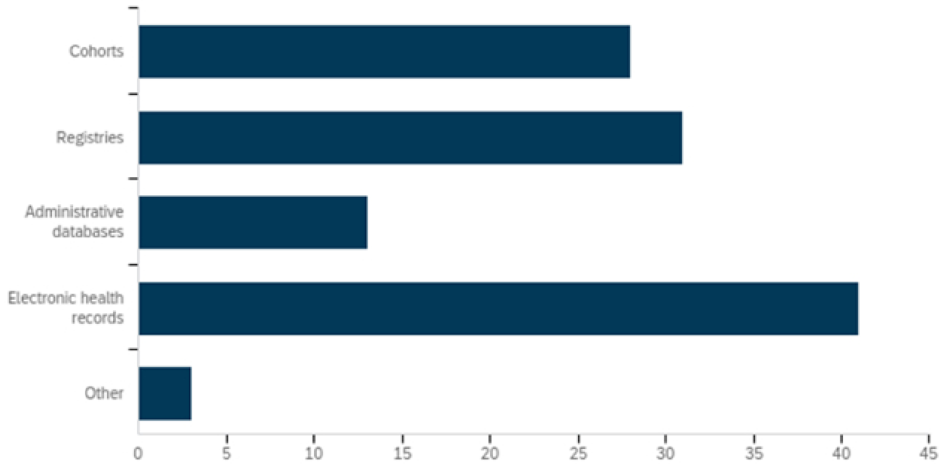
Cohorts
Registries
Administrative databases
Electronic health records
Other
0
5
10
15
20
25
30
35
40
45

Figure 4

Supplementary File 1 – Electronic Search Strategies

Searches were run in both MEDLINE and Cochrane Methodology Register simultaneously. As an example, in the registries search, lines 1-11 are the MEDLINE search and lines 12-15 are tailored for the Cochrane Methodology Register. The final lines of each search isolate the records from each database, combine them so duplicate records can be removed, then isolate the remaining records so they can be downloaded and imported into Reference Manager using customized import filters.

Searches for RCTs embedded in Registries

1. ((registry or registries) adj5 randomi#ed).ab,kf,ti.
2. ((registry or registries) adj5 RCT*).ab,kf,ti.)
3. ((registry or registries) adj5 controlled trial*).ab,kf,ti.
4. ((registry or registries) adj5 (RRCT* or R RCT*)).ab,kf,ti.
5. or/1-4
6. (meta analy* or metaanaly* or metanaly* or systematic review*).af.
7. 5 not 6
8. Registries/
9. limit 8 to randomized controlled trial
10. 7 or 9
11. limit 10 to yr="2007 - 2018"
12. (registry or registries).ab,kf,ti.
13. (random* or RCT).ti,ab,kw.
14. 12 and 13
15. limit 14 to yr="2007 - 2018"
16. 11 use medall
17. 15 use clcmr
18. 16 or 17 (1240)
19. remove duplicates from 18
20. 19 use medall
21. 19 use clcmr

Searches for RCTs embedded in Cohorts

1. (cohort adj5 (randomi#ed adj5 trial*)).ab,kf,ti.
2. (cohort adj5 RCT*).ab,kf,ti.
3. (cohort adj5 controlled trial*).ab,kf,ti.
4. (cmRCT or Cohort Multiple Randomised Controlled Trial*).ab,kf,ti.
5. or/1-4
6. cohort.af.
7. (embed* adj8 randomi#ed).ab,kf,ti.
8. (embed* adj8 RCT*).ab,kf,ti.
9. (embed* adj8 controlled trial*).ab,kf,ti.
10. or/7-9
11. 6 and 10
12. (pragmatic adj5 RCT*).ab,kf,ti.
13. (pragmatic adj5 randomi#ed).ab,kf,ti.

For peer review only

14. (pragmatic adj5 controlled trial*).ab,kf,ti.
15. or/12-14
16. 6 and 15
17. 5 or 11 or 16
18. (meta analy* or metaanaly* or metanaly* or systematic review*).af.
19. 17 not 18
20. limit 19 to yr="2007 - 2018"
21. ((Cohort* and (random* or RCT)) or cmRCT).ti,ab,kw.
22. limit 21 to yr="2007 - 2018"
23. 20 use medall
24. 22 use clcmr
25. 23 or 24
26. remove duplicates from 25
27. 26 use medall
28. 26 use clcmr

Searches for RCTs embedded in Electronic Health Records

1. randomized controlled trial.pt.
2. controlled clinical trial.pt.
3. randomi?ed.ab.
4. placebo.ab.
5. randomly.ab.
6. clinical trials as topic.sh.
7. trial.ti.
8. or/1-7
9. exp animals/ not humans.sh.
10. 8 not 9
11. exp Electronic Health Records/
12. (EHR or electronic health record*).ab,kf,ti.
13. (EMR or electronic medical record*).ab,kf,ti.
14. (PHR or personal health record*).ab,kf,ti.
15. (EPR or electronic patient record*).ab,kf,ti.
16. exp Health Records, Personal/
17. or/11-16
18. 10 and 17
19. limit 18 to yr="2007 - 2018"
20. (Electronic health record or electronic health records or EHR).ti,ab,kw.
21. (Electronic medical record or electronic medical records or EMR).ti,ab,kw.
22. (Electronic patient record or electronic patient records or EPR).ti,ab,kw.
23. or/20-22
24. limit 23 to yr="2007 - 2018"
25. 19 use medall
26. 24 use clcmr
27. 25 or 26
28. remove duplicates from 27
29. 28 use medall
30. 28 use clcmr

For peer review only

Searches for RCTs embedded in Administrative Databases

1. randomized controlled trial.pt.
2. controlled clinical trial.pt.
3. randomi?ed.ab.
4. placebo.ab.
5. randomly.ab.
6. clinical trials as topic.sh.
7. trial.ti.
8. or/1-7
9. exp animals/ not humans.sh.
10. 8 not 9
11. administrative data*.ab,kf,ti.
12. healthcare data*.ab,kf,ti.
13. health care data*.ab,kf,ti.
14. or/11-13
15. 10 and 14
16. (administrative adj5 data*).ti,ab,kw.
17. health care data*.ti,ab,kw.
18. healthcare data*.ti,ab,kw.
19. or/16-18
20. (random* or RCT).ti,ab,kw.
21. 19 and 20
22. limit 15 to yr="2007 - 2018"
23. 22 use medall
24. limit 21 to yr="2007 - 2018"
25. 22 use clcmr

For peer review only
